# Mutation of MUC16 Is Associated With Tumor Mutational Burden and Lymph Node Metastasis in Patients With Gastric Cancer

**DOI:** 10.3389/fmed.2022.836892

**Published:** 2022-02-08

**Authors:** Fengxiang Zhang, Xianzhe Li, Huaxian Chen, Jianping Guo, Zhizhong Xiong, Shi Yin, Longyang Jin, Xijie Chen, Dandong Luo, Haijie Tang, Chaobin Mao, Lei Lian

**Affiliations:** ^1^Department of Gastrointestinal Surgery, The Sixth Affiliated Hospital, Sun Yat-sen University, Guangzhou, China; ^2^Guangdong Provincial Key Laboratory of Colorectal and Pelvic Floor Diseases, The Sixth Affiliated Hospital, Guangdong Institute of Gastroenterology, Sun Yat-sen University, Guangzhou, China

**Keywords:** gastric cancer, lymph node metastasis, tumor mutational burden, MUC16 mutation, tumor microenvironment, immune cells

## Abstract

**Background:**

Lymph node metastasis (LNM) is a critical factor in determining the prognosis of gastric cancer (GC), but its underlying mechanism remains unclear. The tumor mutational burden (TMB) has recently been recognized as a biomarker for predicting prognosis and response to immune checkpoint inhibitors, while mucin 16, cell surface associated (MUC16) is frequently mutated in GC. This study explored whether MUC16 mutation status is associated with TMB, LNM, and prognosis in patients with GC.

**Methods:**

Somatic mutation data were downloaded from three GC cohorts. TMB values were calculated and associations between the TMB and clinical characteristics were analyzed. The mutational landscapes of these three GC cohorts were individually explored and visualized using waterfall diagrams. Univariate logistic regression and Kaplan-Meier survival analysis were performed to screen for mutated genes associated with LNM and overall survival (OS). Associations between MUC16 mutations and TMB, microsatellite instability (MSI), LNM, and tumor microenvironment signatures were explored.

**Results:**

TMB was associated with LNM and OS in patients with GC. Analyzing the three GC cohorts (The Cancer Genome Atlas-Stomach Adenocarcinoma, International Cancer Genome Consortium [ICGC]-China, and ICGC-Japan) revealed that MUC16 was one of the most frequently mutated genes in patients with GC. MUC16 mutations were associated with better prognosis, including lower LNM rates and improved OS rates. In addition, MUC16 mutation status was associated with TMB and MSI statuses. Fifteen upregulated and 222 downregulated genes were identified in patients with MUC16 mutations, compared to in those in patients with wild-type MUC16. An altered tumor microenvironment signature was also identified in GC samples with MUC16 mutations; it was characterized by significantly decreased infiltration regarding stromal cells, CD4+ T cells, and macrophages.

**Conclusion:**

MUC16 mutation status was associated with TMB, microsatellite status, LNM, and survival in patients with GC. These findings may provide new insights into the mechanism of LNM and could act as a signpost for prognostic predictions and immunotherapy guidance for patients with GC.

## Introduction

Gastric cancer (GC) is the fifth most common cancer and the third most common cause of cancer-related deaths globally; it is a global public health problem that seriously threatens human health, especially in East Asia (1–3). The therapeutic effects of traditional treatments for GC, including surgery, chemotherapy, and targeted therapy, remain unsatisfactory; this is especially true in advanced GC cases, which have a short median overall survival (OS) of <1 year (4, 5). Lymph node metastasis (LNM) is one of the most important factors that influences the prognosis and determines the treatment strategy for GC (1, 6). Therefore, there is an urgent need to elucidate the underlying molecular mechanisms of metastasis for GC, and to explore new therapies.

In recent years, the use of immune checkpoint inhibitors (ICIs) has improved the prognoses of various types of cancers, including advanced GC (7). However, not all patients achieve clinical benefits from ICIs, and biomarkers for predicting the effectiveness of immunotherapy in GC are still lacking. The tumor mutational burden (TMB) is an emerging tumor characteristic that refers to the number of somatic mutations per 1 million bases; it has been reported to be associated with microsatellite instability (MSI) (8, 9). Moreover, the TMB appears to be a potential biomarker for predicting prognosis and response to ICIs, as previous studies have identified an association between patients with higher TMB and significantly better responses to ICIs and improved survival (10, 11). Patients with GC have genomic heterogeneity and exhibit various TMBs, and previous studies have recognized that the TMB is a critical determinant in the molecular subtyping of GC (12, 13).

Mucin 16, cell surface associated (MUC16), which is also called CA125, is a type I transmembrane mucin protein. It has been widely used as a tumor-associated marker in ovarian cancer (14). Previous study found that MUC16 was one of the most frequently mutated genes in GC (15). Here, therefore, the relationships between MUC16 mutations and TMB and LNM were investigated in patients with GC.

## Materials and Methods

### Data Collection

“Simple Nucleotide Variation” data from the Stomach Adenocarcinoma (STAD) cohort of The Cancer Genome Atlas (TCGA) (https://gdc.cancer.gov) were downloaded and “Masked Somatic Mutation” data processed with VarScan2 software were selected; they contained 433 cancer tissue samples. Simple somatic mutation data of GC from a Chinese cohort containing 120 samples were downloaded from the International Cancer Genome Consortium (ICGC) (https://dcc.icgc.org/), as were data from a Japanese cohort containing 586 samples (16). The gene expression and clinicopathological profiles of the STAD cohort were also downloaded from TCGA. The MSI states of TCGA-STAD cohort were downloaded from The Cancer Immunome Atlas (https://tcia.at/).

### TMB Calculation

The TMB is defined as the number of somatic, coding, base substitution, and indel mutations per megabyte of tumor tissue (8, 9). Here, the TMB values of each sample were calculated as the number of all mutations/exon length (38 million). In this study, only nonsynonymous mutations were defined as mutation phenotype. Mutation profiles were analyzed using the programming language Perl and were visualized using the “GenVisR” package (https://bioconductor.org/packages/GenVisR/).

### Differential Analysis, Enrichment Analyses, and Protein-Protein Interaction

Differential analysis was performed by comparing patients with MUC16 mutations to those with wild-type MUC16 using the “LIMMA” R package; *p*-values were adjusted using the Benjamini-Hochberg False Discovery Rate (FDR) method (17). Differentially expressed genes (DEGs) were defined as those genes with an adjusted *p* < 0.05 and a log_2_-fold change of < -1 or >1. Next, Gene ontology (GO) and Kyoto Encyclopedia of Genes and Genomes (KEGG) pathway enrichment analyses were performed using the “clusterProfiler” R package (18). The protein-protein interaction (PPI) network of the identified DEGs was also analyzed using the Search Tool for the Retrieval of Interacting Genes (19) (STRING, https://string-db.org/) online database to demonstrate the interaction relationships between DEGs. This PPI network was visualized using Cytoscape software (20).

### Gene Set Enrichment Analyses (GSEA)

To demonstrate the differences in the underlying molecular mechanisms when stratified by MUC16 mutation status, GSEA software version 4.1.0 was used to identify significantly enriched terms in the C5 GO BP, C2 KEGG, and Hallmarks gene set collections from the Molecular Signature Database (21). Gene sets with |NES|>1, NOM *p* < 0.05, and FDR *q* < 0.25 were considered to be significantly enriched; results were visualized using the “ggplot2” R package.

### Tumor Microenvironment and Immune Infiltrates

Infiltrating stromal and immune cells form the major fraction of the tumor microenvironment which plays a critical role in tumorigenesis and progression (22). Here, the Estimation of Stromal and Immune cells in Malignant Tumor tissues using Expression data (ESTIMATE) algorithm was utilized to calculate stromal and immune scores from gene expression data, using the “estimate” R package (23). Tumor Immune Estimation Resource (24) (TIMER, https://cistrome.shinyapps.io/timer/) database, which includes data on 32 types of cancer from TCGA, is an open resource for estimating the abundance of tumor-infiltrating immune cells. Here, TIMER was used to perform correlation analysis between gene mutations and tumor-infiltrating immune cell signatures in TCGA-STAD cohort.

### Statistical Analysis

All statistical analyses were performed using Strawberry Perl (version 5.32.1.1) and R software (version 4.0.3). The Wilcoxon test was used to compare pairs of two independent nonparametric samples. The chi-square test was used to explore the correlations between the categorical variables. Differences in OS between groups were assessed using Kaplan–Meier curves and log-rank tests. Univariate logistic regression analysis was used to identify potential prognostic factors for LNMs. Univariate and multivariate Cox regression analyses were performed to identify the independent prognostic factors for GC. Forest plots were constructed using the “forestplot” R package. Statistical significance was set at *p* <0.05.

## Results

### Associations Between TMB and Clinical Characteristics

**Figure 1:**
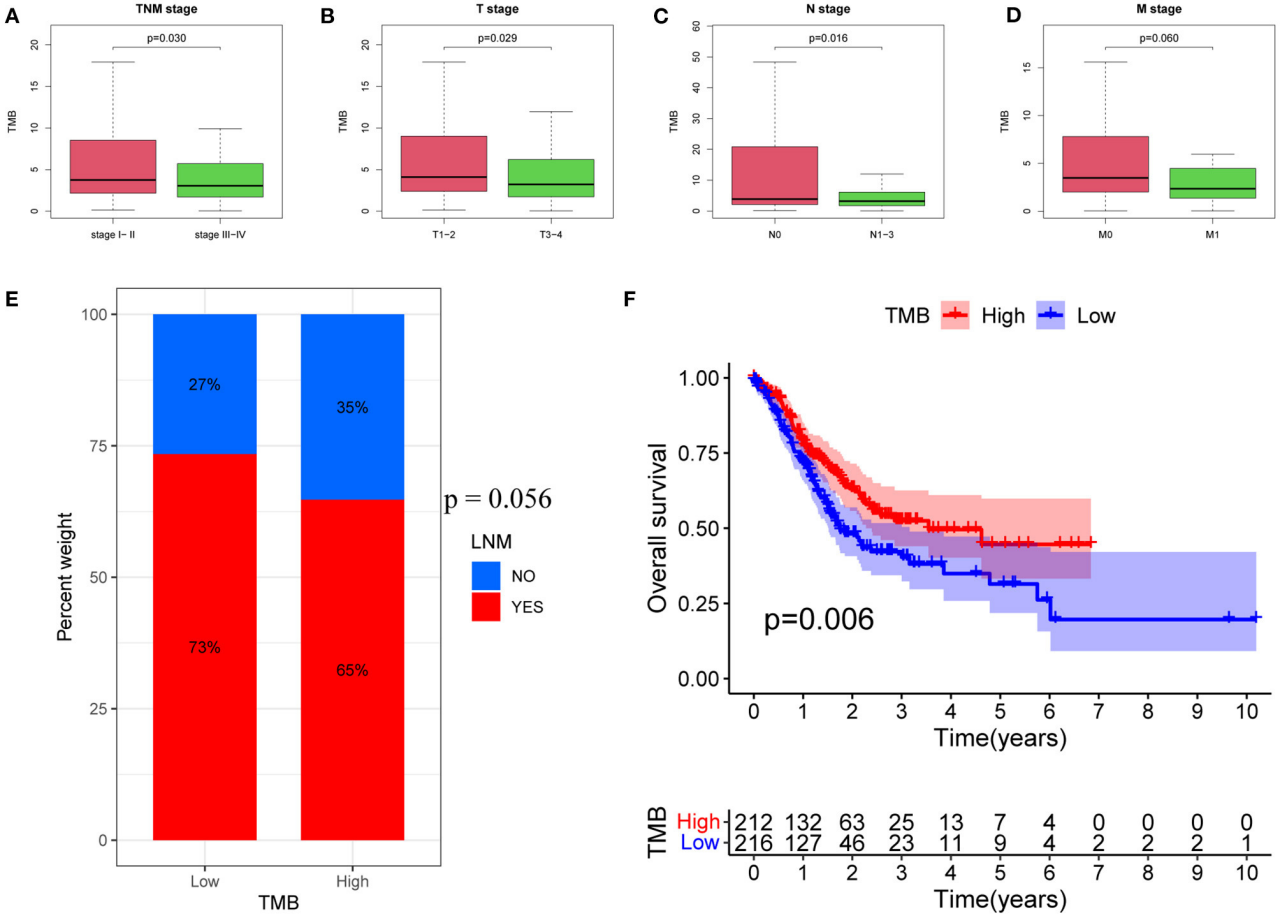
Associations between TMB and clinical characteristics. **(A)** Association between TMB and TNM stage. **(B)** Association between TMB and T stage. **(C)** Association between TMB and N stage. **(D)** Association between TMB and M stage. **(E)** Proportions of patients with LNMs in high- and low-TMB groups. **(F)** Kaplan–Meier curves of OS for high- and low-TMB groups. TMB, tumor mutational burden; LNM, lymph node metastasis; OS, Overall Survival.

Here, analysis of the TMB and tumor characteristics in TCGA-STAD cohort showed that the TMB was significantly lower in patients with advanced GC than in those with early GC (*p* = 0.030; Figure 1A). Specifically, a larger primary tumor size was associated with a lower TMB value (*p* = 0.029; Figure 1B). In addition, GC patients with LNMs had significantly lower TMB values (*p* = 0.016; Figure 1C). Moreover, TMB values tended to be lower in patients with distant metastases (*p* = 0.060; Figure 1D).

Next, patients were divided into low and high TMB groups, according to the median TMB value. The proportion of patients with LNMs tended to be lower in the high TMB group (*p* = 0.056; Figure 1E). Kaplan–Meier analysis further revealed that a higher TMB value was correlated with a better OS (*p* = 0.006; Figure 1F).

### Mutational Landscape of GC

**Figure 2:**
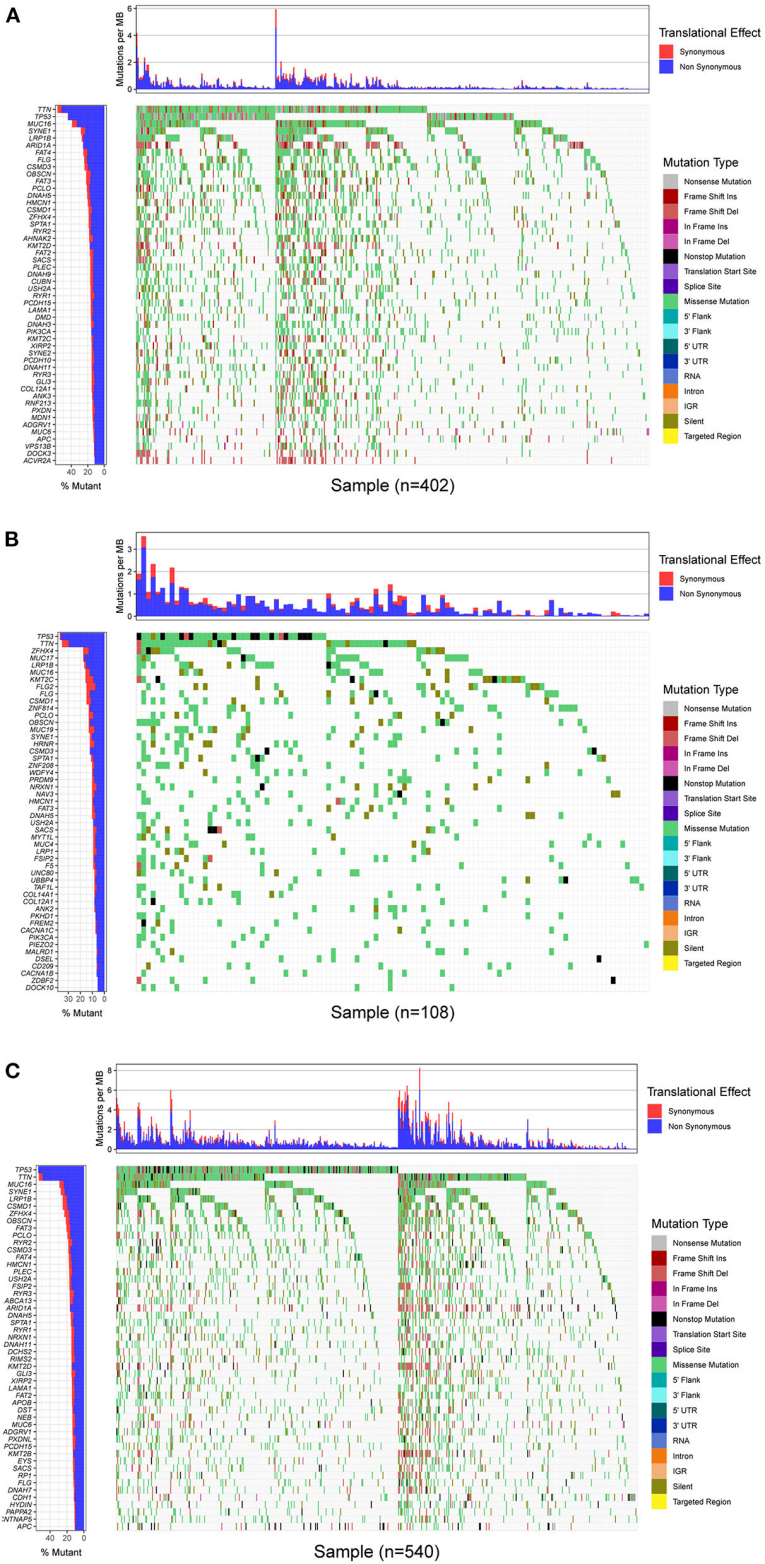
Mutational landscape of GC. **(A)** Waterfall plot of TCGA-STAD cohort. **(B)** Waterfall plot of ICGC-China cohort. **(C)** Waterfall plot of ICGC-China cohort. Annotations on right-hand side represent mutation types. Bar plot on left-hand side shows distribution of mutation types among top 50 genes. GC, gastric cancer; TCGA, The Cancer Genome Atlas; STAD, stomach adenocarcinoma; ICGC, International Cancer Genome Consortium.

In this study, mutational landscapes of three gastric cancer cohorts were analyzed and visualized as the waterfall diagram individually (Figure 2). There was heterogeneity between the three cohorts. The top 10 mutated genes in TCGA-STAD cohort were titin (TTN), tumor protein 53 (TP53), MUC16, AT-rich interaction domain 1A (ARID1A), low-density lipoprotein receptor-related protein 1B (LRP1B), spectrin repeat containing nuclear envelope protein 1 (SYNE1), filaggrin (FLG), FAT atypical cadherin 4 (FAT4), CUB and sushi multiple domains 3 (CSMD3), and piccolo presynaptic cytomatrix protein (PCLO). The top ten mutated genes in the ICGC-China cohort were TP53; TTN; MUC17; LRP1B, zinc finger homeobox 4 (ZFHX4); CSMD3; FLG; zinc finger protein 814 (ZNF814); obscurin, cytoskeletal calmodulin and titin-interacting rhoGEF (OBSCN); and MUC16. The top ten mutated genes in the ICGC-Japan cohort were TP53, TTN, MUC16, SYNE1, LRP1B, CSMD1, ZFHX4, OBSCN, FAT3, and ARID1A.

### Mutated Genes Associated With LNM and Outcomes

**Figure 3:**
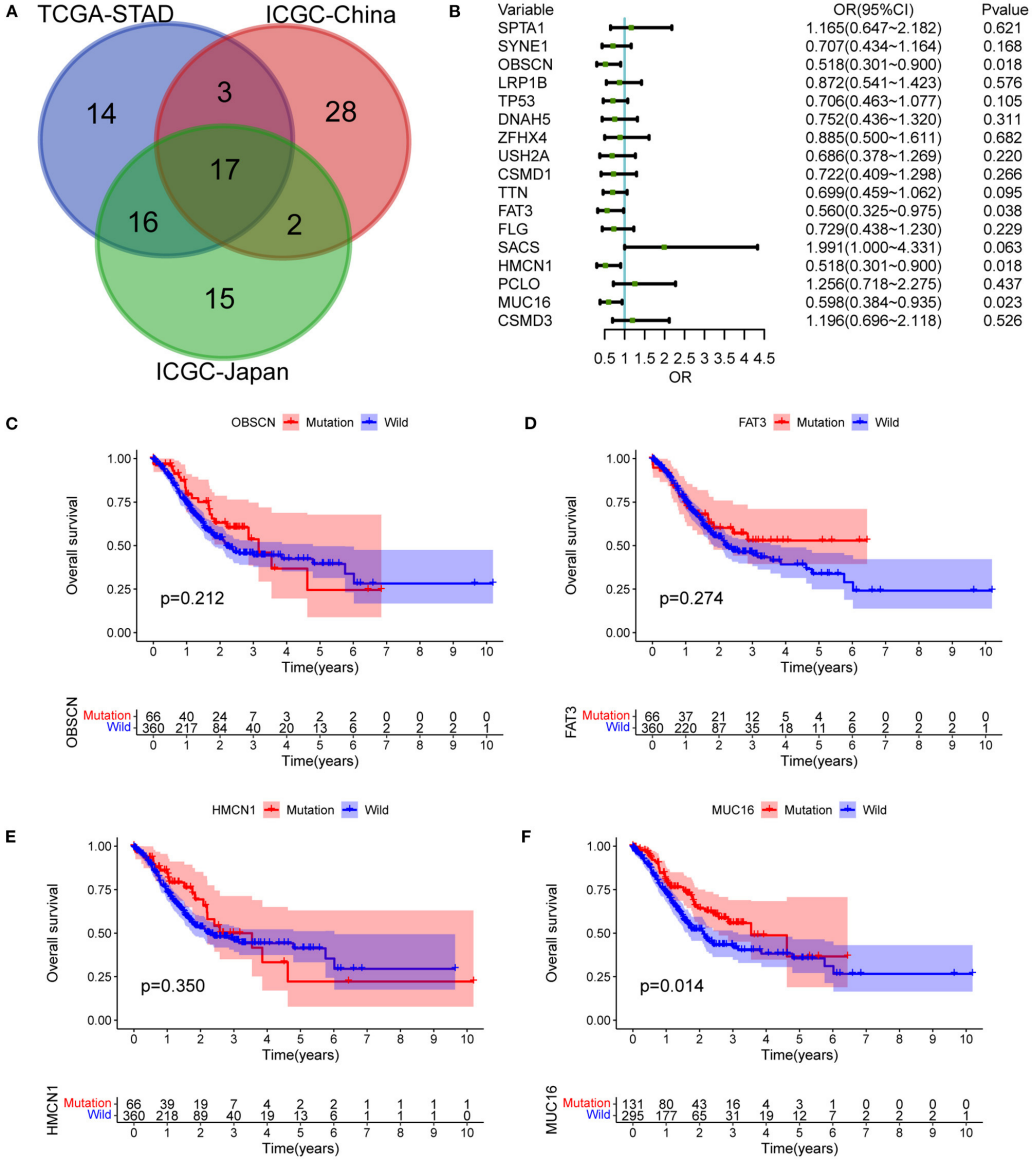
Mutant genes associated with LNMs and outcomes. **(A)** Venn diagram demonstrating intersections of most common mutated genes between three cohorts. **(B)** Univariate logistic regression analysis showing influencing factors for LNMs in TCGA-STAD cohort. **(C)** OS stratified by OBSCN mutation status. **(D)** OS stratified by FAT3 mutation status. **(E)** OS stratified by HMCN1 mutation status. **(F)** OS stratified by MUC16 mutation status. OBSCN, Obscurin, Cytoskeletal Calmodulin And Titin-Interacting RhoGEF; HMCN1, Hemicentin 1; FAT3, FAT Atypical Cadherin 3; MUC16, Mucin 16, Cell Surface Associated.

**Figure 4:**
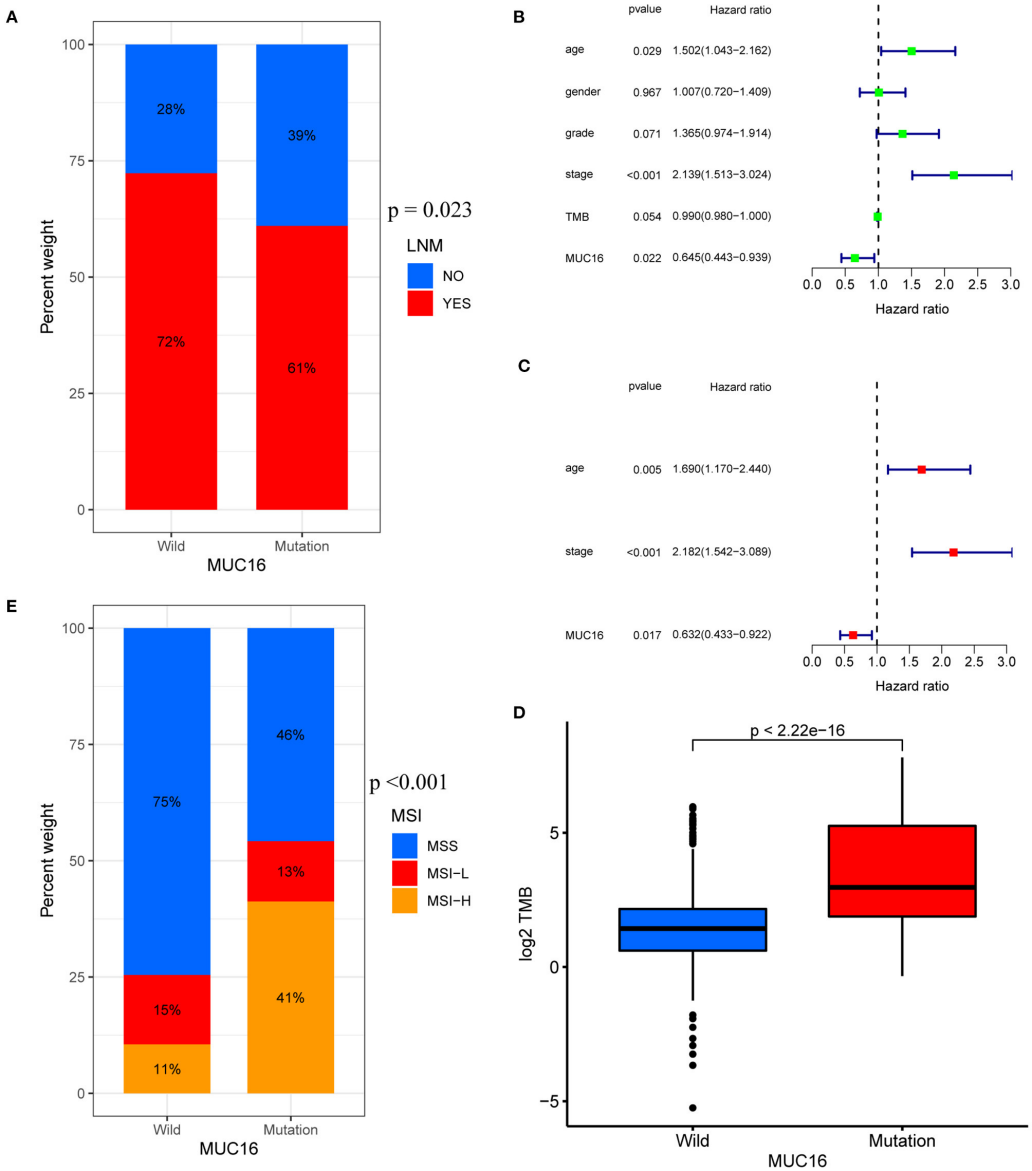
Associations between MUC16 mutation status and clinicopathological characteristics in TCGA-STAD cohort. **(A)** Proportions of patients with LNMs in MUC16 mutations. **(B,C)** Univariate and multivariate Cox regression analyses of independent risk factors. **(D)** Relationship between TMB and MUC16 mutation. **(E)** Association between MUC16 mutation status and MSI. MSI, microsatellite instability.

The top 50 mutated genes in the individual cohort (Supplementary Table 1) were selected and a Venn diagram was plotted to demonstrate the 17 most common mutated genes (Figure 3A and Supplementary Table 2). Univariate logistic regression analysis showed that mutations in OBSCN, FAT3, HMCN1, and MUC16 were influencing factors for LNM in GC (Figure 3B). Among these four genes, Kaplan-Meier survival analysis stratified by MUC16 mutation status showed that MUC16 mutations were significantly associated with a better OS in TCGA cohort (Figures 3C–F). Therefore, we further explored the associations between MUC16 mutation and clinicopathological characteristics. The proportion of patients with LNMs was higher in the MUC16 mutation group than in the wild type MUC16 group (*p* = 0.023; Figure 4A). The Cox regression analysis model revealed that MUC16 mutation was an independent risk factor for OS in TCGA-STAD cohort (HR = 0.632, 95% CI: 0.433-0.922, *p* = 0.017; Figures 4B,C). The TMB was significantly higher in patients with MUC16 mutations than in those with wild-type MUC16 (*p* <0.001; Figure 4D). In addition, the MUC16 mutation status was significantly associated with MSI (*p* <0.001; Figure 4E).

### DEGs, Enrichment Analyses, and PPI

Conducting differential analysis by comparing patients with MUC16 mutations to those with wild-type MUC16 in TCGA-STAD cohort revealed fifteen upregulated and 222 downregulated genes (Supplementary Figures 1A,B, Supplementary Table 3); the PPI network of these DEGs is shown in Supplementary Figure 1C. Next, GO and KEGG enrichment analysis were performed to elucidate the functional and biological pathways of DEGs (Supplementary Figure 2, Supplementary Tables 4, 5). Enriched KEGG pathways were involved in neuroactive ligand-receptor interaction; cardiac muscle contraction; vascular smooth muscle contraction; the cyclic guanosine monophosphate (cGMP)-protein kinase G (PKG) signaling pathway; the cyclic adenosine 3', 5'-monophosphate (cAMP) signaling pathway; adrenergic signaling in cardiomyocytes; cardiac muscle contraction; dilated cardiomyopathy; insulin secretion; and salivary secretion.

### GSEA

**Figure 5:**
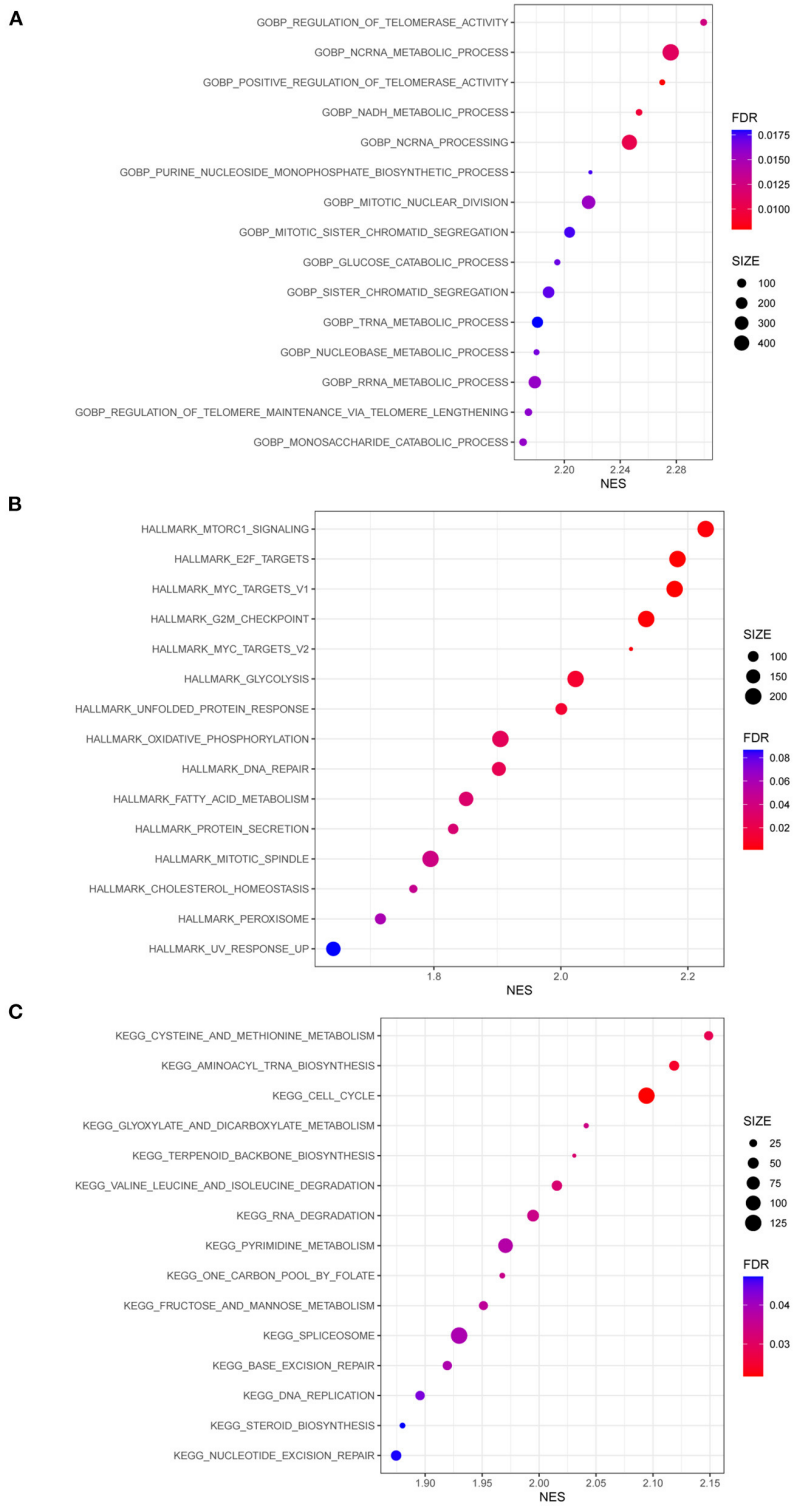
GSEA results of patients with MUC16 mutation in TCGA-STAD cohort. **(A)** Enriched gene sets in GOBP collection. **(B)** Enriched gene sets in HALLMARK collection. **(C)** Enriched gene sets in KEGG collection. GSEA, Gene Set Enrichment Analyses; GO, gene ontology; BP, biological process; KEGG, Kyoto Encyclopedia of Genes and Genomes.

GSEA was conducted to identify differentially regulated pathways stratified by MUC16 mutation status in the TCGA-STAD cohort (Figure 5 and Supplementary Table 6). Among them, we found that the most enriched KEGG pathways in patients with MUC16 mutation were cysteine and methionine metabolism, aminoacyl tRNA biosynthesis, cell cycle, glyoxylate and dicarboxylate metabolism, terpenoid backbone biosynthesis, valine leucine and isoleucine degradation, RNA degradation, pyrimidine metabolism, one carbon pool by folate, fructose and mannose metabolism, spliceosome, base excision repair, DNA replication, steroid biosynthesis and nucleotide excision repair.

### Tumor Microenvironment and Immune Infiltrate Signatures

**Figure 6:**
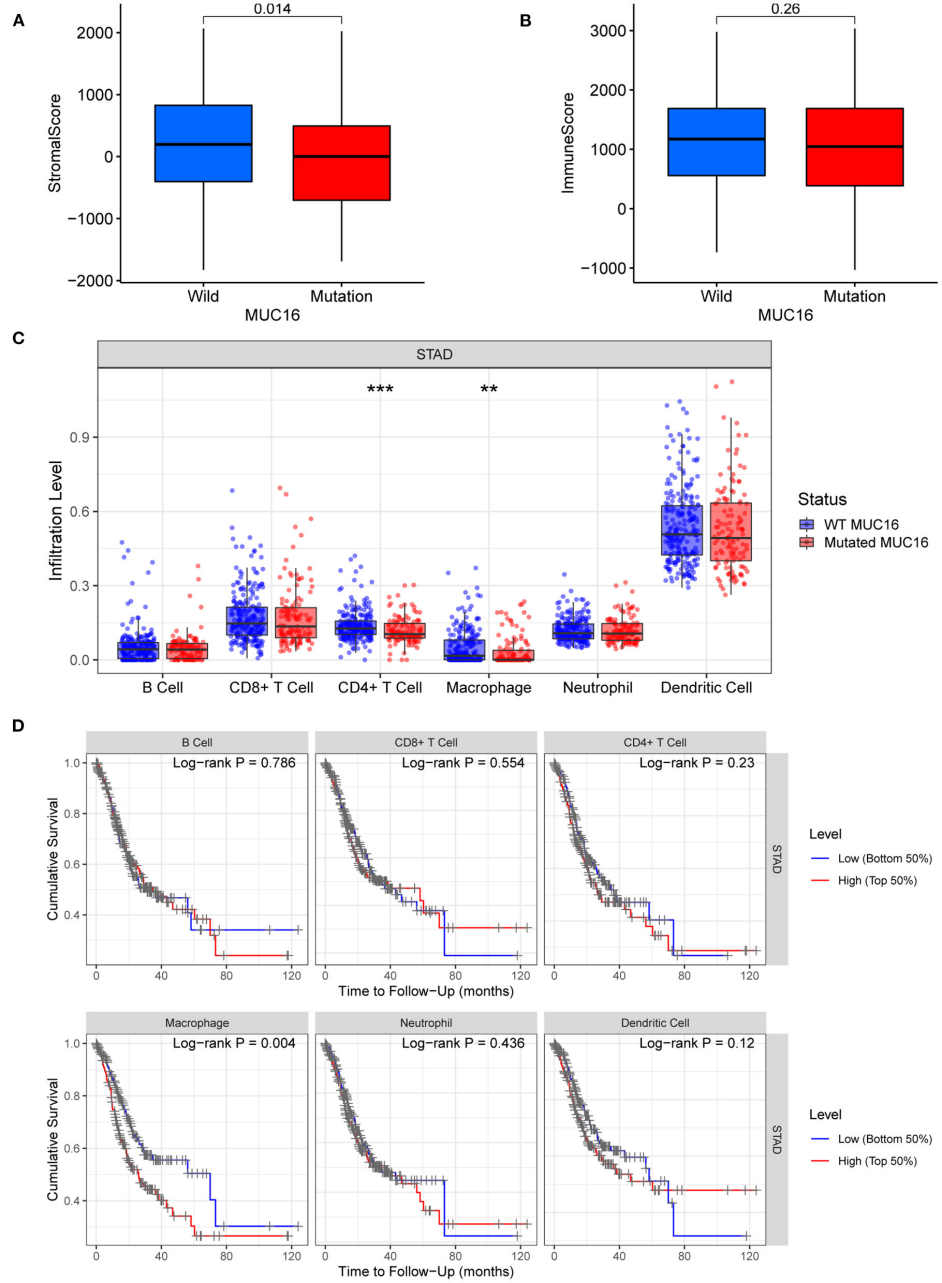
Tumor microenvironment and immune infiltrate signatures of patients with MUC16 mutation in TCGA-STAD cohort. **(A)** Relationships between MUC16 mutation status and stromal scores. **(B)** Relationships between MUC16 mutation status and immune scores. **(C)** Immune infiltrate signatures stratified by MUC16 mutation status. *p < 0.05; **p < 0.01; ***p < 0.001. **(D)** Association between tumor-infiltrating immune cells and survival.

Stromal and immune cells infiltrating in the cancer tissue form the major fraction of the tumor microenvironment which plays an important role in tumor growth, disease progression, drug resistance and anti-tumor immunity (22, 23). In our study, ESTIMATE algorithm was used to calculate stromal scores and immune scores of 375 cancer tissue samples from the TCGA-STAD cohort. Our study showed that stromal scores were significantly lower in patients with MUC16 mutations than in those with wild-type MUC16 (*p* = 0.014; Figure 6A), while immune scores showed no association with MUC16 mutation status (Figure 6B). To ensure a more comprehensive, deeper investigation of the immune infiltrate signatures of GC, here TIMER was used to compare the abundances of six types of tumor-infiltrating immune cells, including B cells, CD4+ T cells, CD8+ T cells, neutrophils, macrophages, and dendritic cells, in patients with different MUC16 mutation statuses. The distributions of CD4+ T cells and macrophage infiltration levels were significantly lower in patients with MUC16 mutations than in those with wild-type MUC16 (*p* < 0.001 and *p* < 0.01, respectively; Figure 6C). Moreover, lower macrophage infiltration levels in GC were associated with a better prognosis (*p* = 0.004; Figure 6D).

## Discussion

By analyzing three cohorts (TCGA-STAD, ICGC-China, and ICGC-Japan), here MUC16 was revealed to be frequently mutated in patients with GC. Moreover, this mutation predicted better prognosis, including lower LNMs and improved survival rates. In addition, MUC16 mutation status was associated with the TMB and the microsatellite status. An altered tumor microenvironment signature was also identified in GC samples with MUC16 mutations, as characterized by decreased infiltration of stromal cells, CD4+ T cells, and macrophages.

GC remains a serious global public health issue, despite recent development and progress regarding diagnosis and treatment (1–3). Most patients (>70%) diagnosed at an advanced stage, while the prognosis of advanced GC is particularly unsatisfactory, with a short median OS of 10–12 months (4, 5, 25). LNM, as the primary pathway for metastasis, has been widely recognized as one of the critical factors in determining the treatment strategy and prognosis of GC (1, 6, 26). Previous studies have shown that LNM is a complicated process that may be involved in changes in cell adhesion, extracellular matrix degradation, new vessel formation, and lymphatic channel permeation (27). However, the underlying molecular mechanism of lymph node metastasis is not completely clear yet, despite its clinical importance.

The distributions of exonic missense mutations display considerable variability among various cancers. On the one hand, driver mutations impart tumor growth and adaption advantages; on the other hand, somatic missense mutations may strongly contribute to the generation of novel tumor epitopes and thus display more neoantigens, which makes them susceptible to immune cells (28). The TMB can represent the neoantigen load to a certain extent. Previous studies have reported that the TMB can be a biomarker for predicting the prognosis and response to ICIs (10, 11). Patients with GC have various TMBs, with this being recognized as a critical determinant in the molecular subtyping of GC (12, 13). Here, the TMB was found to be associated with LNM and outcomes in GC. Furthermore, this study demonstrated that MUC16 was frequently mutated in patients with GC, and that MUC16 mutation status was significantly associated with the TMB; these findings may support the use of MUC16 mutations as a potential and effective surrogate for the TMB, to identify GC patients who might benefit from immune checkpoint blockade. Moreover, here the MUC16 mutation status was also found to be significantly associated with LNM and prognosis.

This study also attempted to uncover the underlying molecular mechanism of MUC16 mutations in GC. Functional enrichment analysis suggested that samples with MUC16 mutations were characterized by upregulated pathways involved in metabolism, cell cycle, and DNA replication and repair. Furthermore, the tumor microenvironment signature was found to be different in GC samples with various MUC16 mutation statuses. Stromal cells and immune cells form the major fraction of the tumor microenvironment of malignant solid tumor tissues and play a crucial role in tumor growth, invasion and metastasis, drug resistance and anti-tumor immunity (22, 23). Specifically, here the infiltration of stromal cells, CD4+ T cells, and macrophages were all observed to decrease significantly in patients with MUC16 mutations. Moreover, lower macrophage infiltration levels were associated with a higher survival rate. The role of tumor-associated macrophages (TAMs) in GC has attracted increasing attention in recent years. TAMs can be divided into two main types: M1 and M2. In GC, M2 TAMs are characterized by an immunosuppressive and pro-angiogenic phenotype that promotes tumor growth, invasiveness, and drug resistance (29–31). The mechanism by which MUC16 mutations influence the microenvironment and LNM in GC warrants further study.

This study has some limitations that should be considered when interpreting the presented results. First, there was heterogeneity among the three cohorts. Second, the findings were mainly based on the bioinformatics analysis of publicly available data; they need to be validated by basic laboratory experiments and clinical trials. Finally, the observed associations between the MUC16 mutation status and the TMB or microsatellite status do not necessarily imply causation, and a functional explanation is currently lacking. However, despite these limitations, the findings of this study suggest that the prognostic relevance of the MUC16 mutation status in GC is robust.

## Conclusion

In conclusion, we discovered that MUC16 mutations were frequently found in patients with GC, which were associated with lower LNMs and improved survival. In addition, MUC16 mutation status was associated with TMB and microsatellite status. We also identified an altered tumor microenvironment signature in GC samples with MUC16 mutations. Our findings may provide new insights into the mechanisms of LNM and a signpost for prognostic prediction and clinical guidance for patients with GC.

## Data Availability Statement

The datasets presented in this study can be found in online repositories. The names of the repository/repositories and accession number(s) can be found in the article/Supplementary Material.

## Author Contributions

FZ, XL, and HC analyzed the data, designed the study, and wrote the manuscript. JG, ZX, SY, and LJ collected the data and critically revised the manuscript. XC, DL, HT, and CM explained the results and critically revised the manuscript. LL participated in the conception of the study, designed the study, and revised the manuscript critically. All authors have read and approved the final submitted manuscript.

## Funding

This study was supported by the Guangdong Natural Science Fund for Outstanding Youth Scholars (Grant no. 2020B151502067) and the Bethune Aixikang Distinguished Surgical Fund project (Grant no. HZB-20190528-5).

## Conflict of Interest

The authors declare that the research was conducted in the absence of any commercial or financial relationships that could be construed as a potential conflict of interest.

## Publisher's Note

All claims expressed in this article are solely those of the authors and do not necessarily represent those of their affiliated organizations, or those of the publisher, the editors and the reviewers. Any product that may be evaluated in this article, or claim that may be made by its manufacturer, is not guaranteed or endorsed by the publisher.

## Abbreviations

GC: gastric cancer
LNM: lymph node metastasis
TMB: tumor mutational burden
ICI: immune checkpoint inhibitors
GO: Gene Ontology
KEGG: Kyoto Encyclopedia of Genes and Genomes
GSEA: gene set enrichment analysis
TCGA: The Cancer Genome Atlas
STAD: stomach adenocarcinoma
ICGC: International Cancer Genome Consortium
TIMER: Tumor Immune Estimation Resource
OS: Overall Survival
MSI: Microsatellite Instability
MUC16: gene mucin 16, cell surface associated.
