# Supplementary material for: Mutation of MUC16 Is Associated With Tumor Mutational Burden and Lymph Node Metastasis in Patients With Gastric Cancer
Source: Front Med (Lausanne). 2022 Feb 8;9:836892. doi: 10.3389/fmed.2022.836892 (PMC8863212; doi:10.3389/fmed.2022.836892)
Supplement: Supplementary file 1 [file Data_Sheet_1.docx]

Supplementary Material

# Supplementary Figures and Tables

## Supplementary Figures


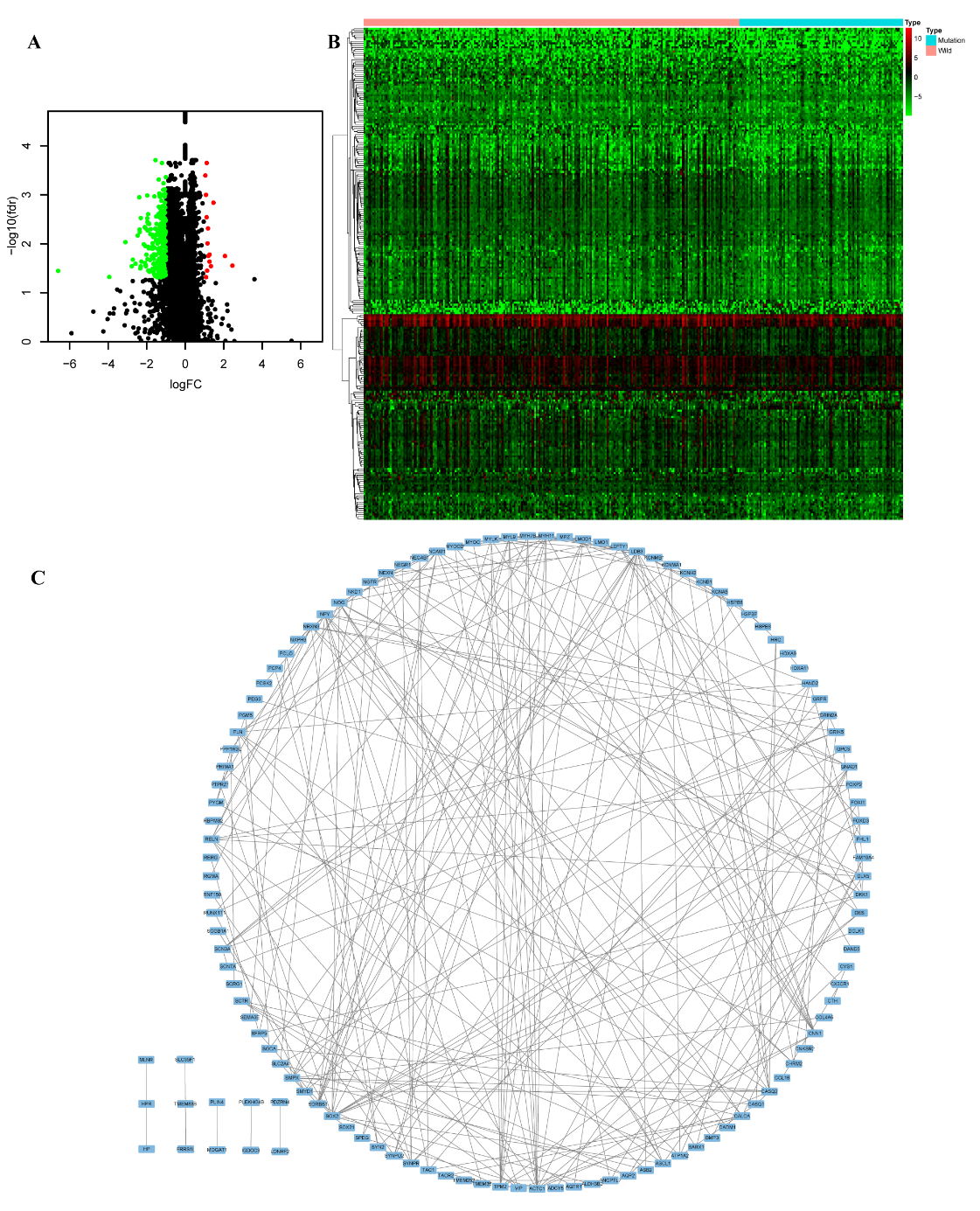


Supplementary Figure 1. Analysis of differential expression profiles between patients with various MUC16 mutation status in the TCGA-STAD cohort. (A) Volcano plots of differentially expressed genes (DEGs) between patients with various MUC16 mutation status. (B) Heatmap of DEGs between patients with various MUC16 mutation status. (C) Protein-protein interaction network.


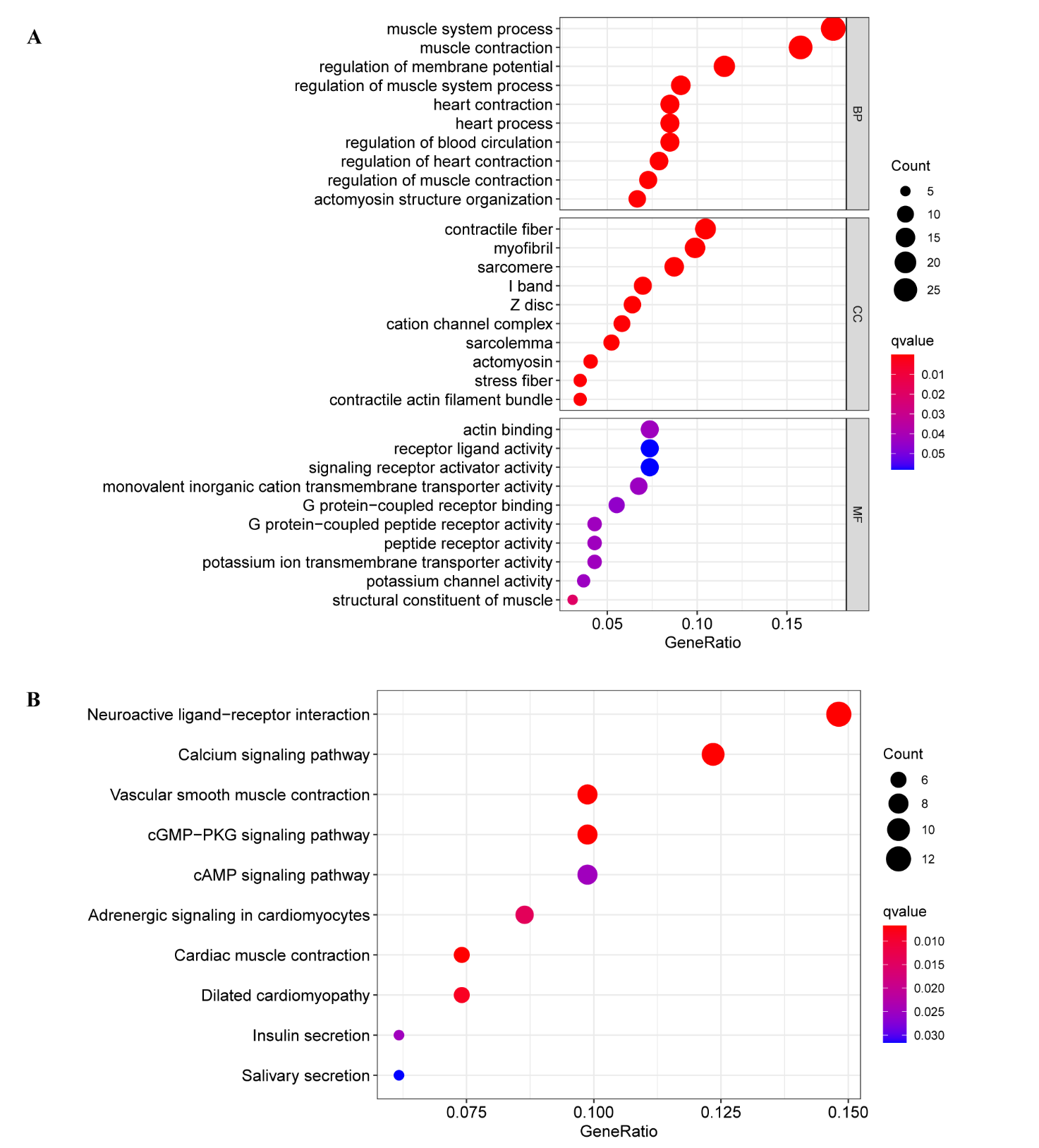


Supplementary Figure 2. GO terms and KEGG pathway analysis. (A) The most enriched GO BP terms. (B) The most enriched KEGG pathway. GO, gene ontology; KEGG, Kyoto Encyclopedia of Genes and Genomes

## Supplementary Tables

Supplementary Table 1. Top 50 mutated genes in the three gastric cancer cohorts

| Cohort | the top 50 mutated genes |
| --- | --- |
| TCGA-STAD | TTN, TP53, MUC16, ARID1A, LRP1B, SYNE1, FLG, FAT4, CSMD3, PCLO, DNAH5, KMT2D, FAT3, HMCN1, OBSCN, RYR2, ZFHX4, SPTA1, PIK3CA, CSMD1, PCDH15, DMD, USH2A, PLEC, AHNAK2, XIRP2, DNAH9, SACS, LAMA1, CUBN, KMT2C, ANK3, FAT2, DNAH11, DNAH3, COL12A1, SYNE2, RNF213, PCDH10, MDN1, RYR3, ADGRV1, VPS13B, RYR1, GLI3, ACVR2A, MUC6, DOCK3, PXDN, APC |
| ICGC-China | TP53, TTN, MUC17, LRP1B, ZFHX4, CSMD3, FLG, ZNF814, OBSCN, MUC16, CSMD1, SYNE1, SPTA1, HMCN1, USH2A, KMT2C, WDFY4, PCLO, FAT3, PRDM9, COL12A1, FSIP2, ZNF208, MUC19, NAV3, HRNR, DNAH5, MYT1L, COL14A1, MUC4, ANK2, FLG2, F5, PKHD1, UBBP4, PIK3CA, FREM2, CACNA1B, LRP1, DSEL, SACS, TAF1L, UNC80, PIEZO2, CACNA1C, NRXN1, DOCK10, MALRD1, ZDBF2, CD209 |
| ICGC-Japan | TP53, TTN, MUC16, SYNE1, LRP1B, CSMD1, ZFHX4, OBSCN, FAT3, ARID1A, PCLO, CSMD3, FSIP2, USH2A, FAT4, HMCN1, PLEC, RYR2, KMT2D, SPTA1, ABCA13, KMT2B, DST, DNAH5, RYR3, DNAH11, NRXN1, XIRP2, APOB, ADGRV1, DCHS2, RP1, RYR1, CDH1, LAMA1, EYS, FAT2, SACS, RIMS2, DNAH7, NEB, FLG, MUC6, PCDH15, GLI3, APC, HYDIN, CNTNAP5, PAPPA2, PXDNL |

TCGA, The Cancer Genome Atlas; STAD, stomach adenocarcinoma; ICGC, the International Cancer Genome Consortium.

Supplementary Table 2. The most common mutated genes among the three gastric cancer cohorts

| Number | the most common mutated genes |
| --- | --- |
| 17 | SPTA1, SYNE1, OBSCN, LRP1B, TP53, DNAH5, ZFHX4, USH2A, CSMD1, TTN, FAT3, FLG, SACS, HMCN1, PCLO, MUC16, CSMD3 |

Supplementary Table 3. Differentially expressed genes between patients with various MUC16 mutation status

| gene | conMean | treatMean | logFC | pValue | fdr |
| --- | --- | --- | --- | --- | --- |
| HP | 0.928814824 | 0.15537921 | -2.5796 | 0.003373 | 0.025549 |
| RELN | 0.701505622 | 0.164949258 | -2.08843 | 0.003175 | 0.024647 |
| LINC02600 | 0.479882428 | 0.17704605 | -1.43856 | 0.009573 | 0.048272 |
| GPR27 | 1.365076058 | 0.625541287 | -1.1258 | 1.64E-06 | 0.000579 |
| PRIMA1 | 2.435608404 | 0.949226505 | -1.35946 | 0.000272 | 0.006095 |
| ZNF385B | 0.55941222 | 0.240607502 | -1.21723 | 0.007786 | 0.042559 |
| FAM83C | 0.475344316 | 0.181557672 | -1.38855 | 0.006397 | 0.03772 |
| RNU6-946P | 0.387103495 | 0.180501155 | -1.10071 | 0.000153 | 0.004463 |
| RNF150 | 1.850391379 | 0.837046669 | -1.14445 | 0.000108 | 0.003717 |
| INSYN1 | 0.471461048 | 0.207065642 | -1.18705 | 5.51E-06 | 0.000993 |
| PYGM | 0.821981148 | 0.344855084 | -1.25312 | 0.000114 | 0.003832 |
| GFRA3 | 2.455332198 | 0.953662155 | -1.36437 | 2.00E-05 | 0.001744 |
| AC105460.1 | 8.818521017 | 19.73044485 | 1.161815 | 0.000648 | 0.009772 |
| H3P16 | 4.606169746 | 10.60260493 | 1.202779 | 0.001861 | 0.017701 |
| SYN2 | 0.8960801 | 0.296574221 | -1.59523 | 7.36E-06 | 0.001075 |
| DKK1 | 16.80378543 | 6.484108271 | -1.37381 | 1.02E-06 | 0.000485 |
| GRPR | 0.426340455 | 0.211751029 | -1.00964 | 0.008025 | 0.043455 |
| AC253536.4 | 1.361929952 | 0.408073512 | -1.73875 | 0.001002 | 0.012427 |
| CST2 | 7.166108419 | 3.572946085 | -1.00408 | 0.002001 | 0.018486 |
| TCEAL2 | 2.423249568 | 1.033786505 | -1.229 | 0.00136 | 0.014947 |
| CCL16 | 0.41471105 | 0.060594095 | -2.77486 | 0.004071 | 0.028718 |
| ZNF750 | 2.00056153 | 0.784858839 | -1.3499 | 0.00227 | 0.020013 |
| PDZRN4 | 0.8453481 | 0.301297188 | -1.48836 | 0.004962 | 0.032015 |
| KCNB1 | 0.350335363 | 0.133993276 | -1.38658 | 4.09E-05 | 0.002467 |
| BARX1 | 21.19981117 | 9.079122464 | -1.22343 | 0.001736 | 0.016891 |
| PTCH2 | 1.457716766 | 0.712484502 | -1.03278 | 5.24E-06 | 0.000956 |
| SYNM | 25.8977637 | 6.652227732 | -1.96092 | 0.0001 | 0.003559 |
| FOXD3 | 0.325317747 | 0.136449022 | -1.25349 | 5.72E-06 | 0.001 |
| ASB2 | 3.160585058 | 1.38485905 | -1.19045 | 0.000859 | 0.011431 |
| LONRF2 | 0.588532971 | 0.271546758 | -1.11592 | 6.76E-06 | 0.001037 |
| MYOCD | 2.429764844 | 1.015490747 | -1.25864 | 0.003838 | 0.027696 |
| NXPH3 | 0.914473396 | 0.41874269 | -1.12688 | 0.000416 | 0.007551 |
| SCTR | 1.254259751 | 0.442033532 | -1.50461 | 5.17E-05 | 0.002763 |
| NGFR | 3.376099118 | 1.391697417 | -1.27851 | 6.53E-05 | 0.002986 |
| FILIP1 | 1.927293335 | 0.951225336 | -1.01872 | 8.94E-05 | 0.003401 |
| POPDC2 | 2.701213298 | 1.134676095 | -1.25133 | 0.000263 | 0.005946 |
| CYP17A1 | 3.478382459 | 0.404836702 | -3.103 | 0.000588 | 0.009227 |
| NPTX1 | 1.159217515 | 0.455737335 | -1.34688 | 4.45E-06 | 0.000938 |
| AC105460.2 | 0.190116091 | 0.432031553 | 1.184256 | 0.000179 | 0.004848 |
| PEG3 | 0.635113791 | 0.256964386 | -1.30545 | 0.000143 | 0.004359 |
| AL022313.2 | 0.739725885 | 0.235890095 | -1.64888 | 0.003395 | 0.02565 |
| AL049870.3 | 0.525444118 | 0.250689248 | -1.06764 | 0.000334 | 0.00673 |
| METTL24 | 1.202251421 | 0.532528341 | -1.17481 | 0.000158 | 0.004506 |
| SLC35F1 | 0.394752246 | 0.179050713 | -1.14058 | 3.09E-06 | 0.000788 |
| MPZ | 2.305171301 | 0.714194479 | -1.69049 | 0.005028 | 0.032253 |
| ALDH3B2 | 3.805217404 | 1.848293764 | -1.04178 | 0.007164 | 0.040414 |
| RUNX1T1 | 0.912864623 | 0.435959413 | -1.06621 | 8.02E-06 | 0.001094 |
| AC053503.5 | 0.722922318 | 0.149680186 | -2.27196 | 0.002843 | 0.023032 |
| MYLK | 19.49808527 | 7.675683187 | -1.34497 | 0.000585 | 0.0092 |
| AC016813.1 | 0.522911891 | 0.211198446 | -1.30797 | 0.000441 | 0.007804 |
| ANXA8L1 | 0.478436125 | 0.199145389 | -1.2645 | 0.00118 | 0.013757 |
| HSPB8 | 14.04865373 | 5.497145283 | -1.35368 | 0.000481 | 0.00822 |
| TMEM35A | 0.87539178 | 0.338682291 | -1.37 | 5.29E-05 | 0.002783 |
| PLIN4 | 4.449817037 | 1.632709862 | -1.44648 | 0.004563 | 0.030503 |
| NRXN3 | 1.097686829 | 0.413290564 | -1.40924 | 0.001065 | 0.012872 |
| MYOC | 1.811433619 | 0.795915989 | -1.18644 | 0.005167 | 0.032851 |
| CX3CR1 | 1.013832961 | 0.501092186 | -1.01667 | 0.000226 | 0.005529 |
| AL512324.1 | 0.294047102 | 0.707672783 | 1.267035 | 0.001682 | 0.016593 |
| NKD1 | 6.938606451 | 1.837101986 | -1.91721 | 0.005722 | 0.035186 |
| SCN3A | 0.288212963 | 0.134266629 | -1.10203 | 0.001296 | 0.014552 |
| AL032819.2 | 0.757066867 | 0.152792974 | -2.30884 | 0.004066 | 0.028695 |
| AF001548.1 | 1.376727451 | 0.490664421 | -1.48843 | 0.00012 | 0.003926 |
| NOG | 0.465276915 | 0.213851891 | -1.12148 | 0.002858 | 0.023059 |
| AP001107.5 | 0.395395631 | 0.176293301 | -1.16532 | 0.000224 | 0.005515 |
| IVL | 3.188099042 | 1.519681079 | -1.06893 | 0.004757 | 0.031227 |
| AL590004.3 | 0.671639162 | 0.313663424 | -1.09847 | 3.00E-05 | 0.002117 |
| KCNMA1 | 3.341987837 | 1.325807664 | -1.33383 | 0.002429 | 0.02082 |
| C8orf88 | 1.938091677 | 0.801688578 | -1.27352 | 3.39E-05 | 0.002245 |
| SCRG1 | 1.632797444 | 0.584922755 | -1.48103 | 0.000517 | 0.008569 |
| HRC | 0.41145809 | 0.205247697 | -1.00338 | 6.03E-07 | 0.000434 |
| GRIN2A | 0.331600392 | 0.132930675 | -1.31877 | 6.18E-06 | 0.001025 |
| DES | 434.3178692 | 119.2622622 | -1.86461 | 0.005741 | 0.035249 |
| FOXD3-AS1 | 0.465081851 | 0.187354041 | -1.31172 | 7.88E-06 | 0.001094 |
| AC120036.3 | 0.463437971 | 0.207282367 | -1.16078 | 4.63E-05 | 0.002643 |
| MOGAT1 | 0.355808282 | 0.133328512 | -1.41611 | 0.002758 | 0.022558 |
| KCNMB1 | 4.64170678 | 1.975008994 | -1.2328 | 0.002286 | 0.020097 |
| AC004637.1 | 0.262208207 | 0.117808897 | -1.15426 | 0.000675 | 0.01004 |
| PPP1R3C | 3.421377815 | 1.652058684 | -1.05031 | 3.62E-05 | 0.002298 |
| DLX5 | 0.929107079 | 0.350181113 | -1.40774 | 0.000236 | 0.005648 |
| FOXI1 | 0.556900842 | 0.084489758 | -2.72057 | 0.002476 | 0.021044 |
| AQP2 | 0.780173163 | 4.266530396 | 2.451197 | 0.003869 | 0.027847 |
| SYNPR | 0.561721384 | 0.109560435 | -2.35813 | 0.000244 | 0.005722 |
| ACTA2-AS1 | 1.577609822 | 0.779860932 | -1.01645 | 0.000541 | 0.008787 |
| SMYD1 | 1.476331007 | 0.365881101 | -2.01257 | 0.001677 | 0.016591 |
| TAC1 | 0.599757244 | 0.155519027 | -1.94729 | 0.000213 | 0.005331 |
| PCSK2 | 0.347273436 | 0.062902487 | -2.46488 | 0.000337 | 0.006759 |
| AF001548.3 | 0.914629653 | 0.241366921 | -1.92196 | 0.000862 | 0.011463 |
| PCLO | 0.894786608 | 0.414391187 | -1.11055 | 0.000115 | 0.003864 |
| FHL1 | 22.70340955 | 8.840830285 | -1.36066 | 0.001407 | 0.015209 |
| AC012531.6 | 0.210085926 | 0.454196334 | 1.112337 | 5.64E-05 | 0.002868 |
| AP005018.2 | 2.359751674 | 0.822553295 | -1.52045 | 0.000311 | 0.006535 |
| ADCY5 | 3.344281485 | 1.423640879 | -1.23211 | 1.15E-05 | 0.001324 |
| GPR87 | 1.262331017 | 0.432334796 | -1.54587 | 0.003292 | 0.025175 |
| GPC3 | 19.10069914 | 7.832908111 | -1.28601 | 0.000336 | 0.006749 |
| PGM5-AS1 | 1.270142018 | 0.29633951 | -2.09967 | 0.000161 | 0.004548 |
| LINC02106 | 0.304804256 | 0.134928738 | -1.17569 | 0.001796 | 0.017264 |
| CNKSR2 | 0.281436892 | 0.111455573 | -1.33634 | 0.000334 | 0.00673 |
| HSPB6 | 99.13237252 | 26.12606671 | -1.92387 | 4.13E-05 | 0.002476 |
| KCNA5 | 0.553994927 | 0.250854701 | -1.14302 | 8.59E-05 | 0.003341 |
| VIP | 2.647649035 | 0.902312981 | -1.55301 | 0.008755 | 0.045799 |
| BARX1-DT | 0.448558534 | 0.18355379 | -1.28909 | 0.001484 | 0.015623 |
| SGCA | 2.57220741 | 1.099964521 | -1.22555 | 0.00013 | 0.004142 |
| LINC02677 | 0.61937152 | 0.040323569 | -3.94111 | 0.009326 | 0.047563 |
| DACT3 | 3.286856987 | 1.602474958 | -1.03641 | 0.000295 | 0.006339 |
| PLN | 12.73846373 | 5.538609491 | -1.2016 | 0.004382 | 0.029906 |
| AC104336.1 | 0.807849827 | 0.384310522 | -1.07181 | 0.000205 | 0.005238 |
| MYH11 | 132.574248 | 37.43234684 | -1.82444 | 0.002517 | 0.021311 |
| HOXA11-AS | 0.952631309 | 1.967965719 | 1.046715 | 4.28E-07 | 0.000403 |
| OGN | 14.25214388 | 5.899348025 | -1.27255 | 2.25E-05 | 0.00181 |
| CCDC154 | 0.455126754 | 0.145698192 | -1.64329 | 0.006051 | 0.036459 |
| IGDCC3 | 0.383118753 | 0.077478236 | -2.30593 | 6.18E-05 | 0.002968 |
| FBXL22 | 1.531729795 | 0.722442931 | -1.08421 | 0.000144 | 0.004359 |
| AP003071.4 | 1.060315212 | 0.474400516 | -1.16032 | 0.004449 | 0.030137 |
| MLNR | 0.324883412 | 0.140987188 | -1.20436 | 0.000175 | 0.004803 |
| RPL35AP4 | 0.50022263 | 0.233193314 | -1.10104 | 6.09E-05 | 0.002955 |
| NPY | 0.973097995 | 0.343324098 | -1.50301 | 0.000524 | 0.008623 |
| SLC2A4 | 2.090780524 | 0.845102838 | -1.30684 | 0.00111 | 0.013177 |
| MRGPRF | 10.52035793 | 5.159068377 | -1.028 | 0.00611 | 0.03666 |
| AL035425.4 | 0.484551228 | 0.092711293 | -2.38583 | 8.42E-06 | 0.001121 |
| AGTR1 | 0.801745735 | 0.322399701 | -1.31429 | 0.003461 | 0.025998 |
| AC009549.1 | 0.323220651 | 0.136791572 | -1.24054 | 0.00015 | 0.004436 |
| HOXA9 | 1.126129206 | 3.117643265 | 1.469083 | 1.34E-05 | 0.001446 |
| SCN7A | 0.554956009 | 0.257961468 | -1.10522 | 0.002294 | 0.020144 |
| NCAM1 | 1.040822914 | 0.411667753 | -1.33817 | 0.000212 | 0.00531 |
| AVIL | 1.783369097 | 0.764566784 | -1.22189 | 0.000164 | 0.004601 |
| SEMA3E | 1.286667481 | 0.611420394 | -1.0734 | 0.000483 | 0.008233 |
| CASQ2 | 2.74519905 | 0.86944267 | -1.65875 | 0.00039 | 0.007264 |
| LINC02700 | 0.221418587 | 0.532088878 | 1.264891 | 0.002866 | 0.023095 |
| CLDN19 | 0.266894001 | 0.129858399 | -1.03933 | 0.002458 | 0.020945 |
| LEFTY1 | 3.599008002 | 15.08373693 | 2.067323 | 0.001875 | 0.017749 |
| AC109486.1 | 0.258753976 | 0.112466546 | -1.20209 | 0.00392 | 0.028054 |
| REEP2 | 1.561793869 | 0.74345865 | -1.07088 | 0.000814 | 0.011147 |
| PDZD4 | 1.012526164 | 0.456412806 | -1.14955 | 3.59E-05 | 0.002295 |
| RPRM | 0.492259344 | 0.19771341 | -1.31601 | 0.000265 | 0.00598 |
| LINC01671 | 0.755489081 | 0.315049576 | -1.26183 | 0.009583 | 0.048306 |
| BBOX1 | 0.398235193 | 0.194577258 | -1.03328 | 0.000578 | 0.009112 |
| RN7SKP255 | 0.643659466 | 0.275435615 | -1.22458 | 0.000203 | 0.00521 |
| SYNPO2 | 27.68188516 | 9.170933261 | -1.5938 | 0.000925 | 0.011957 |
| MYH7B | 0.923089752 | 0.337251476 | -1.45265 | 0.000189 | 0.005026 |
| CHRM2 | 0.981279169 | 0.307140506 | -1.67576 | 0.001013 | 0.012496 |
| SFRP5 | 2.992641297 | 1.028735621 | -1.54055 | 2.68E-08 | 0.000195 |
| SHISA3 | 3.383607877 | 1.391439926 | -1.28198 | 0.005952 | 0.036135 |
| AC078993.1 | 0.44436203 | 0.213017602 | -1.06076 | 0.002356 | 0.020509 |
| PCP4 | 2.775222911 | 1.013119755 | -1.4538 | 0.009281 | 0.047427 |
| RGMA | 4.480647317 | 1.717682754 | -1.38324 | 5.08E-05 | 0.002734 |
| SOX21 | 3.387170276 | 1.688334765 | -1.00448 | 0.002512 | 0.021282 |
| AC092071.1 | 0.26525785 | 0.124172868 | -1.09505 | 0.008195 | 0.044 |
| SMPX | 1.97361309 | 0.476994575 | -2.04879 | 0.003668 | 0.026898 |
| MIR1-1HG-AS1 | 0.32574409 | 0.089025698 | -1.87145 | 0.000117 | 0.003875 |
| TAFA4 | 0.515244353 | 0.099713303 | -2.3694 | 0.000197 | 0.005118 |
| RNU7-47P | 0.359796651 | 0.155191847 | -1.21313 | 0.00013 | 0.00413 |
| NEXN | 10.29338 | 5.103863384 | -1.01206 | 0.007836 | 0.042765 |
| CYS1 | 3.095901795 | 1.230616329 | -1.33098 | 0.001014 | 0.012496 |
| TACR2 | 7.562360931 | 1.949612732 | -1.95565 | 0.002378 | 0.020599 |
| AC008808.2 | 0.904908112 | 0.441839896 | -1.03425 | 2.22E-05 | 0.0018 |
| TMEM252 | 0.858112557 | 0.373768283 | -1.19902 | 0.006232 | 0.037093 |
| SPEG | 1.693131262 | 0.773778274 | -1.1297 | 0.008565 | 0.045127 |
| CASQ1 | 0.306749737 | 0.121644296 | -1.33439 | 0.001184 | 0.013794 |
| LINC02520 | 0.364406158 | 0.082200266 | -2.14833 | 0.0007 | 0.010214 |
| CTH | 2.367802256 | 5.004872008 | 1.079785 | 5.89E-06 | 0.001 |
| AC005180.1 | 0.571600308 | 0.245693259 | -1.21815 | 0.008453 | 0.044852 |
| GLDC | 2.038325484 | 0.943327086 | -1.11155 | 0.008839 | 0.046026 |
| ECRG4 | 6.594390386 | 1.684554929 | -1.96887 | 6.85E-05 | 0.003016 |
| AC244502.1 | 0.276330609 | 0.083640598 | -1.72412 | 0.000305 | 0.006479 |
| RBPMS2 | 9.630557324 | 3.797054802 | -1.34274 | 0.000204 | 0.005233 |
| CNN1 | 108.6220925 | 30.03764535 | -1.85447 | 0.006231 | 0.037093 |
| EPHA7 | 1.264108851 | 0.346201217 | -1.86844 | 0.00012 | 0.003934 |
| SEMG2 | 0.200908499 | 0.426405435 | 1.085687 | 0.00945 | 0.04795 |
| AL138930.1 | 0.839015464 | 0.315367833 | -1.41166 | 0.003171 | 0.024647 |
| RERG | 2.848681375 | 1.36672993 | -1.05957 | 2.93E-06 | 0.00077 |
| NECAB1 | 0.539172422 | 0.212960111 | -1.34016 | 4.57E-05 | 0.002635 |
| PRG4 | 0.704631453 | 0.322307191 | -1.12843 | 0.00293 | 0.023388 |
| AC005180.2 | 0.643163316 | 0.282585707 | -1.1865 | 0.000183 | 0.00493 |
| SERTM2 | 0.263815836 | 0.064224363 | -2.03834 | 0.004075 | 0.028731 |
| AC023105.1 | 0.373180914 | 0.165615513 | -1.17204 | 1.86E-05 | 0.001705 |
| CST5 | 0.357633326 | 0.116383625 | -1.61959 | 0.00163 | 0.016342 |
| RNU6-977P | 0.697998993 | 0.303912098 | -1.19957 | 0.002561 | 0.021584 |
| LMO1 | 0.43327386 | 0.11050225 | -1.9712 | 6.41E-06 | 0.00103 |
| NPY6R | 0.441305282 | 0.144784887 | -1.60787 | 0.007607 | 0.041925 |
| GRIK5 | 0.962606663 | 0.445493875 | -1.11154 | 0.000426 | 0.007684 |
| DAND5 | 0.276111298 | 0.11144788 | -1.30888 | 0.006416 | 0.037751 |
| SLC13A2 | 4.201951746 | 1.573067502 | -1.41748 | 0.007857 | 0.042834 |
| LINC00702 | 0.750823334 | 0.278398872 | -1.43132 | 0.000657 | 0.009837 |
| AC005006.1 | 0.712152079 | 0.268157841 | -1.4091 | 0.00455 | 0.03047 |
| TEX19 | 0.160223922 | 0.40522523 | 1.338634 | 0.004046 | 0.028589 |
| PLEKHG4B | 0.327811772 | 0.1237929 | -1.40494 | 0.004687 | 0.030958 |
| DCLK1 | 0.774839308 | 0.331302848 | -1.22575 | 6.71E-05 | 0.003 |
| ASCL1 | 5.795093361 | 1.152181643 | -2.33046 | 0.004903 | 0.031802 |
| GNAO1 | 2.323494199 | 1.022138253 | -1.18471 | 3.25E-05 | 0.002211 |
| LINC01579 | 0.265926325 | 0.09259411 | -1.52203 | 0.000331 | 0.006723 |
| FOXP2 | 0.765842446 | 0.316332407 | -1.27561 | 5.77E-05 | 0.002891 |
| SHISA2 | 3.704960493 | 1.601020763 | -1.21047 | 0.001841 | 0.017564 |
| HAND2-AS1 | 0.992860976 | 0.267916399 | -1.88981 | 0.000981 | 0.01233 |
| CARMN | 1.194575248 | 0.465355644 | -1.36009 | 0.000976 | 0.012267 |
| NELL1 | 0.449839743 | 0.108852546 | -2.04704 | 0.000177 | 0.004837 |
| LDB3 | 1.587316942 | 0.492723356 | -1.68774 | 0.000824 | 0.011175 |
| PGM5 | 5.598040837 | 1.953976126 | -1.51851 | 0.000495 | 0.008352 |
| COL4A6 | 0.96866463 | 0.404484879 | -1.25991 | 0.000264 | 0.005971 |
| ADAMTS18 | 0.36511849 | 0.124294654 | -1.5546 | 0.000181 | 0.004903 |
| ANGPTL7 | 0.414176696 | 0.206053943 | -1.00722 | 0.000243 | 0.005706 |
| HOXA11 | 1.575596198 | 3.423550909 | 1.119596 | 1.33E-07 | 0.000223 |
| COX7B2 | 1.008657677 | 0.428846999 | -1.2339 | 0.006449 | 0.037909 |
| SLCO1B3 | 0.544915939 | 1.20230617 | 1.141699 | 0.005742 | 0.035249 |
| AC002398.2 | 0.445309917 | 0.137900445 | -1.69118 | 0.000638 | 0.00969 |
| PART1 | 0.461942 | 0.205921076 | -1.16562 | 0.002482 | 0.021079 |
| TPM2 | 75.46898229 | 35.38463587 | -1.09276 | 0.003608 | 0.026683 |
| PI16 | 3.21558443 | 1.598465182 | -1.00839 | 0.001207 | 0.013916 |
| CADM1 | 2.588064861 | 1.278491147 | -1.01743 | 0.0009 | 0.011807 |
| LMOD1 | 27.29033124 | 9.71035902 | -1.49079 | 0.001668 | 0.016547 |
| MYL9 | 216.8466502 | 92.48390714 | -1.2294 | 0.001631 | 0.016342 |
| ATP1A2 | 1.756994046 | 0.508092434 | -1.78995 | 0.000188 | 0.005019 |
| ACTC1 | 1.813292056 | 0.88057027 | -1.0421 | 0.007095 | 0.040145 |
| STUM | 0.686363709 | 0.315589158 | -1.12093 | 8.00E-05 | 0.003243 |
| RN7SKP203 | 1.07483518 | 0.5053864 | -1.08866 | 0.001672 | 0.016577 |
| PTPRZ1 | 1.00974954 | 0.464028259 | -1.12171 | 0.000621 | 0.009548 |
| CA4 | 2.114434004 | 0.670967213 | -1.65596 | 0.006354 | 0.037586 |
| FRRS1L | 0.47812188 | 0.136283834 | -1.81076 | 0.000295 | 0.006339 |
| HPR | 0.26688259 | 0.064022988 | -2.05954 | 0.000825 | 0.011175 |
| BMP3 | 1.045356371 | 0.27575968 | -1.92251 | 0.000115 | 0.003869 |
| CALCA | 5.723341145 | 0.059158037 | -6.59614 | 0.005848 | 0.035684 |
| SCGB1A1 | 1.603427562 | 0.536807277 | -1.57868 | 0.004395 | 0.029949 |
| HSPB7 | 10.6715338 | 3.731212314 | -1.51605 | 0.001736 | 0.016891 |
| SORBS1 | 16.59909663 | 7.022746259 | -1.241 | 0.00056 | 0.008963 |
| AC036108.3 | 0.633870329 | 0.231047786 | -1.456 | 0.000207 | 0.005249 |
| HLF | 1.128099622 | 0.54741064 | -1.0432 | 0.006011 | 0.036325 |
| KCNH2 | 4.622679588 | 2.210325916 | -1.06447 | 6.38E-05 | 0.002982 |
| TMEM88B | 0.463637818 | 0.182452818 | -1.34547 | 0.004909 | 0.031811 |
| HAND2 | 4.994602027 | 1.616428662 | -1.62756 | 0.002315 | 0.020282 |
| SOX2 | 8.812883157 | 3.572005296 | -1.30288 | 2.39E-05 | 0.001873 |
| LINC01996 | 0.927288475 | 0.421299364 | -1.13817 | 0.001243 | 0.0142 |
| MORN5 | 0.955970722 | 0.286758403 | -1.73713 | 0.001888 | 0.017838 |
| DIO1 | 0.437457742 | 0.171928031 | -1.34734 | 0.003125 | 0.024381 |
| C5orf66-AS1 | 1.454313246 | 0.568843606 | -1.35423 | 0.001332 | 0.014756 |
| PGM5P4 | 0.78876595 | 0.34195262 | -1.2058 | 1.05E-07 | 0.000223 |
| NEGR1 | 1.177346116 | 0.585901311 | -1.00681 | 8.29E-05 | 0.003291 |
| ANGPTL1 | 2.765692927 | 1.091439642 | -1.34141 | 0.000238 | 0.005649 |
| AC138649.1 | 0.238671132 | 0.115403369 | -1.04834 | 1.33E-05 | 0.001446 |

Supplementary Table 4. Gene ontology annotation results of differentially expressed genes between patients with various MUC16 mutation status

| Ontology | ID | Description | Count | p.adjust |
| --- | --- | --- | --- | --- |
| BP | GO:0003012 | muscle system process | 29 | 1.82E-13 |
| BP | GO:0006936 | muscle contraction | 26 | 1.82E-13 |
| BP | GO:0090257 | regulation of muscle system process | 15 | 5.94E-06 |
| BP | GO:0042391 | regulation of membrane potential | 19 | 5.94E-06 |
| BP | GO:0006937 | regulation of muscle contraction | 12 | 1.82E-05 |
| BP | GO:0060047 | heart contraction | 14 | 8.37E-05 |
| BP | GO:0003015 | heart process | 14 | 0.000105 |
| BP | GO:0008016 | regulation of heart contraction | 13 | 0.000105 |
| BP | GO:1903522 | regulation of blood circulation | 14 | 0.000105 |
| BP | GO:0031032 | actomyosin structure organization | 11 | 0.00036 |
| BP | GO:0042692 | muscle cell differentiation | 15 | 0.00036 |
| BP | GO:0007611 | learning or memory | 12 | 0.000571 |
| BP | GO:0060537 | muscle tissue development | 15 | 0.000571 |
| BP | GO:0030239 | myofibril assembly | 7 | 0.000571 |
| BP | GO:0007517 | muscle organ development | 15 | 0.000571 |
| BP | GO:0006941 | striated muscle contraction | 10 | 0.000573 |
| BP | GO:0006939 | smooth muscle contraction | 8 | 0.000807 |
| BP | GO:0010880 | regulation of release of sequestered calcium ion into cytosol by sarcoplasmic reticulum | 5 | 0.001072 |
| BP | GO:0014706 | striated muscle tissue development | 14 | 0.001193 |
| BP | GO:0051146 | striated muscle cell differentiation | 12 | 0.001362 |
| BP | GO:0050890 | cognition | 12 | 0.001437 |
| BP | GO:0035637 | multicellular organismal signaling | 10 | 0.001455 |
| BP | GO:0014808 | release of sequestered calcium ion into cytosol by sarcoplasmic reticulum | 5 | 0.001526 |
| BP | GO:1903514 | release of sequestered calcium ion into cytosol by endoplasmic reticulum | 5 | 0.001679 |
| BP | GO:0006940 | regulation of smooth muscle contraction | 6 | 0.002125 |
| BP | GO:0001508 | action potential | 8 | 0.002125 |
| BP | GO:0034765 | regulation of ion transmembrane transport | 15 | 0.002213 |
| BP | GO:0007200 | phospholipase C-activating G protein-coupled receptor signaling pathway | 7 | 0.002436 |
| BP | GO:0002027 | regulation of heart rate | 7 | 0.002508 |
| BP | GO:0070296 | sarcoplasmic reticulum calcium ion transport | 5 | 0.002842 |
| BP | GO:0007612 | learning | 8 | 0.003312 |
| BP | GO:0010881 | regulation of cardiac muscle contraction by regulation of the release of sequestered calcium ion | 4 | 0.003896 |
| BP | GO:0010927 | cellular component assembly involved in morphogenesis | 7 | 0.005064 |
| BP | GO:0030509 | BMP signaling pathway | 8 | 0.005297 |
| BP | GO:0051480 | regulation of cytosolic calcium ion concentration | 12 | 0.005345 |
| BP | GO:0007519 | skeletal muscle tissue development | 8 | 0.005712 |
| BP | GO:0070252 | actin-mediated cell contraction | 7 | 0.006227 |
| BP | GO:0030178 | negative regulation of Wnt signaling pathway | 9 | 0.006447 |
| BP | GO:0010882 | regulation of cardiac muscle contraction by calcium ion signaling | 4 | 0.006727 |
| BP | GO:0055075 | potassium ion homeostasis | 4 | 0.006727 |
| BP | GO:0030326 | embryonic limb morphogenesis | 7 | 0.006727 |
| BP | GO:0035113 | embryonic appendage morphogenesis | 7 | 0.006727 |
| BP | GO:0007204 | positive regulation of cytosolic calcium ion concentration | 11 | 0.006812 |
| BP | GO:0060538 | skeletal muscle organ development | 8 | 0.006812 |
| BP | GO:0071772 | response to BMP | 8 | 0.006812 |
| BP | GO:0071773 | cellular response to BMP stimulus | 8 | 0.006812 |
| BP | GO:0019932 | second-messenger-mediated signaling | 13 | 0.007003 |
| BP | GO:0090075 | relaxation of muscle | 4 | 0.007003 |
| BP | GO:0055002 | striated muscle cell development | 8 | 0.007003 |
| BP | GO:0030510 | regulation of BMP signaling pathway | 6 | 0.007003 |
| BP | GO:0051899 | membrane depolarization | 6 | 0.007003 |
| BP | GO:2000052 | positive regulation of non-canonical Wnt signaling pathway | 3 | 0.007999 |
| BP | GO:0010959 | regulation of metal ion transport | 12 | 0.008863 |
| BP | GO:0060048 | cardiac muscle contraction | 7 | 0.01054 |
| BP | GO:0055001 | muscle cell development | 8 | 0.01054 |
| BP | GO:0007409 | axonogenesis | 13 | 0.011071 |
| BP | GO:0007178 | transmembrane receptor protein serine/threonine kinase signaling pathway | 11 | 0.011071 |
| BP | GO:0030048 | actin filament-based movement | 7 | 0.011071 |
| BP | GO:2000095 | regulation of Wnt signaling pathway, planar cell polarity pathway | 3 | 0.011071 |
| BP | GO:0006813 | potassium ion transport | 9 | 0.011089 |
| BP | GO:0090092 | regulation of transmembrane receptor protein serine/threonine kinase signaling pathway | 9 | 0.011246 |
| BP | GO:0086010 | membrane depolarization during action potential | 4 | 0.01143 |
| BP | GO:0061337 | cardiac conduction | 7 | 0.011684 |
| BP | GO:0043266 | regulation of potassium ion transport | 6 | 0.011932 |
| BP | GO:0055119 | relaxation of cardiac muscle | 3 | 0.011932 |
| BP | GO:0035107 | appendage morphogenesis | 7 | 0.011932 |
| BP | GO:0035108 | limb morphogenesis | 7 | 0.011932 |
| BP | GO:0045926 | negative regulation of growth | 9 | 0.012812 |
| BP | GO:0055067 | monovalent inorganic cation homeostasis | 7 | 0.014726 |
| BP | GO:0050804 | modulation of chemical synaptic transmission | 12 | 0.016736 |
| BP | GO:0099177 | regulation of trans-synaptic signaling | 12 | 0.016838 |
| BP | GO:0007389 | pattern specification process | 12 | 0.019871 |
| BP | GO:0071805 | potassium ion transmembrane transport | 8 | 0.022139 |
| BP | GO:0019722 | calcium-mediated signaling | 8 | 0.022505 |
| BP | GO:0110110 | positive regulation of animal organ morphogenesis | 5 | 0.0231 |
| BP | GO:0006874 | cellular calcium ion homeostasis | 12 | 0.023245 |
| BP | GO:0007411 | axon guidance | 9 | 0.023245 |
| BP | GO:0051279 | regulation of release of sequestered calcium ion into cytosol | 5 | 0.023245 |
| BP | GO:0048485 | sympathetic nervous system development | 3 | 0.023245 |
| BP | GO:0097485 | neuron projection guidance | 9 | 0.023399 |
| BP | GO:0001503 | ossification | 11 | 0.023895 |
| BP | GO:0060065 | uterus development | 3 | 0.025763 |
| BP | GO:0003018 | vascular process in circulatory system | 7 | 0.025912 |
| BP | GO:1904062 | regulation of cation transmembrane transport | 10 | 0.025912 |
| BP | GO:0010171 | body morphogenesis | 4 | 0.026284 |
| BP | GO:0086009 | membrane repolarization | 4 | 0.026284 |
| BP | GO:0055074 | calcium ion homeostasis | 12 | 0.0264 |
| BP | GO:0090090 | negative regulation of canonical Wnt signaling pathway | 7 | 0.027121 |
| BP | GO:2000050 | regulation of non-canonical Wnt signaling pathway | 3 | 0.027121 |
| BP | GO:0048736 | appendage development | 7 | 0.02717 |
| BP | GO:0060173 | limb development | 7 | 0.02717 |
| BP | GO:0030514 | negative regulation of BMP signaling pathway | 4 | 0.028183 |
| BP | GO:0055078 | sodium ion homeostasis | 4 | 0.028183 |
| BP | GO:1901019 | regulation of calcium ion transmembrane transporter activity | 5 | 0.028217 |
| BP | GO:0001505 | regulation of neurotransmitter levels | 10 | 0.030329 |
| BP | GO:0072503 | cellular divalent inorganic cation homeostasis | 12 | 0.035057 |
| BP | GO:0030111 | regulation of Wnt signaling pathway | 10 | 0.035057 |
| BP | GO:0086065 | cell communication involved in cardiac conduction | 4 | 0.035106 |
| BP | GO:0060314 | regulation of ryanodine-sensitive calcium-release channel activity | 3 | 0.035106 |
| BP | GO:0097553 | calcium ion transmembrane import into cytosol | 6 | 0.035271 |
| BP | GO:0006942 | regulation of striated muscle contraction | 5 | 0.037779 |
| BP | GO:0046879 | hormone secretion | 9 | 0.037779 |
| BP | GO:0035296 | regulation of tube diameter | 6 | 0.037779 |
| BP | GO:0097746 | regulation of blood vessel diameter | 6 | 0.037779 |
| BP | GO:0035150 | regulation of tube size | 6 | 0.038766 |
| BP | GO:0045843 | negative regulation of striated muscle tissue development | 4 | 0.039628 |
| BP | GO:0001708 | cell fate specification | 5 | 0.039804 |
| BP | GO:0055123 | digestive system development | 6 | 0.040414 |
| BP | GO:0007215 | glutamate receptor signaling pathway | 5 | 0.040636 |
| BP | GO:0048635 | negative regulation of muscle organ development | 4 | 0.040636 |
| BP | GO:0009914 | hormone transport | 9 | 0.042226 |
| BP | GO:0010644 | cell communication by electrical coupling | 3 | 0.042226 |
| BP | GO:0045822 | negative regulation of heart contraction | 3 | 0.042226 |
| BP | GO:0030900 | forebrain development | 10 | 0.042226 |
| BP | GO:0010522 | regulation of calcium ion transport into cytosol | 5 | 0.042226 |
| BP | GO:0035567 | non-canonical Wnt signaling pathway | 6 | 0.042748 |
| BP | GO:0060393 | regulation of pathway-restricted SMAD protein phosphorylation | 4 | 0.042748 |
| BP | GO:1901862 | negative regulation of muscle tissue development | 4 | 0.042748 |
| BP | GO:0032412 | regulation of ion transmembrane transporter activity | 8 | 0.042894 |
| BP | GO:0007218 | neuropeptide signaling pathway | 5 | 0.044054 |
| BP | GO:0007588 | excretion | 4 | 0.044212 |
| BP | GO:0048645 | animal organ formation | 4 | 0.046455 |
| BP | GO:0046677 | response to antibiotic | 9 | 0.047642 |
| BP | GO:0051148 | negative regulation of muscle cell differentiation | 4 | 0.047985 |
| BP | GO:0060389 | pathway-restricted SMAD protein phosphorylation | 4 | 0.047985 |
| BP | GO:0022898 | regulation of transmembrane transporter activity | 8 | 0.048825 |
| BP | GO:0043271 | negative regulation of ion transport | 6 | 0.049062 |
| BP | GO:0051926 | negative regulation of calcium ion transport | 4 | 0.049062 |
| BP | GO:0060325 | face morphogenesis | 3 | 0.049062 |
| BP | GO:0061311 | cell surface receptor signaling pathway involved in heart development | 3 | 0.049062 |
| BP | GO:0060402 | calcium ion transport into cytosol | 6 | 0.049487 |
| BP | GO:0048662 | negative regulation of smooth muscle cell proliferation | 4 | 0.050747 |
| BP | GO:0035914 | skeletal muscle cell differentiation | 4 | 0.05315 |
| BP | GO:0048640 | negative regulation of developmental growth | 5 | 0.055739 |
| BP | GO:0060795 | cell fate commitment involved in formation of primary germ layer | 3 | 0.055739 |
| BP | GO:0014877 | response to muscle inactivity involved in regulation of muscle adaptation | 2 | 0.055739 |
| BP | GO:0014894 | response to denervation involved in regulation of muscle adaptation | 2 | 0.055739 |
| BP | GO:0009952 | anterior/posterior pattern specification | 7 | 0.056101 |
| BP | GO:0010721 | negative regulation of cell development | 9 | 0.05895 |
| BP | GO:0051954 | positive regulation of amine transport | 3 | 0.058964 |
| CC | GO:0043292 | contractile fiber | 18 | 5.91E-10 |
| CC | GO:0030016 | myofibril | 17 | 1.54E-09 |
| CC | GO:0030017 | sarcomere | 15 | 2.71E-08 |
| CC | GO:0031674 | I band | 12 | 2.85E-07 |
| CC | GO:0030018 | Z disc | 11 | 1.10E-06 |
| CC | GO:0042383 | sarcolemma | 9 | 0.000125 |
| CC | GO:0042641 | actomyosin | 7 | 0.000203 |
| CC | GO:0034703 | cation channel complex | 10 | 0.000633 |
| CC | GO:0001725 | stress fiber | 6 | 0.000633 |
| CC | GO:0097517 | contractile actin filament bundle | 6 | 0.000633 |
| CC | GO:1902495 | transmembrane transporter complex | 12 | 0.000635 |
| CC | GO:1990351 | transporter complex | 12 | 0.000739 |
| CC | GO:0032432 | actin filament bundle | 6 | 0.000928 |
| CC | GO:0034705 | potassium channel complex | 6 | 0.00341 |
| CC | GO:0034702 | ion channel complex | 10 | 0.005237 |
| CC | GO:0016529 | sarcoplasmic reticulum | 5 | 0.005944 |
| CC | GO:0043034 | costamere | 3 | 0.008186 |
| CC | GO:0016528 | sarcoplasm | 5 | 0.00916 |
| CC | GO:0014704 | intercalated disc | 4 | 0.011806 |
| CC | GO:0008076 | voltage-gated potassium channel complex | 5 | 0.011806 |
| CC | GO:0034706 | sodium channel complex | 3 | 0.011806 |
| CC | GO:0032279 | asymmetric synapse | 9 | 0.026686 |
| CC | GO:0044291 | cell-cell contact zone | 4 | 0.036594 |
| CC | GO:0098984 | neuron to neuron synapse | 9 | 0.037268 |
| CC | GO:0031225 | anchored component of membrane | 6 | 0.037268 |
| CC | GO:0098839 | postsynaptic density membrane | 4 | 0.03757 |
| CC | GO:0043025 | neuronal cell body | 11 | 0.039227 |
| CC | GO:0033017 | sarcoplasmic reticulum membrane | 3 | 0.041258 |
| CC | GO:0001518 | voltage-gated sodium channel complex | 2 | 0.053223 |
| CC | GO:0005791 | rough endoplasmic reticulum | 4 | 0.053223 |
| CC | GO:0005859 | muscle myosin complex | 2 | 0.05528 |
| CC | GO:0090533 | cation-transporting ATPase complex | 2 | 0.05528 |
| CC | GO:0045211 | postsynaptic membrane | 8 | 0.05528 |
| CC | GO:0014069 | postsynaptic density | 8 | 0.05528 |
| MF | GO:0008307 | structural constituent of muscle | 5 | 0.021797 |
| MF | GO:0008528 | G protein-coupled peptide receptor activity | 7 | 0.047332 |
| MF | GO:0001653 | peptide receptor activity | 7 | 0.047332 |
| MF | GO:0003779 | actin binding | 12 | 0.047332 |
| MF | GO:0015079 | potassium ion transmembrane transporter activity | 7 | 0.047332 |
| MF | GO:0015077 | monovalent inorganic cation transmembrane transporter activity | 11 | 0.048177 |
| MF | GO:0005267 | potassium channel activity | 6 | 0.048177 |
| MF | GO:0001664 | G protein-coupled receptor binding | 9 | 0.05074 |

Supplementary Table 5. KEGG pathway analysis results of differentially expressed genes between patients with various MUC16 mutation status

| ID | Description | Count | p.adjust |
| --- | --- | --- | --- |
| hsa04270 | Vascular smooth muscle contraction | 8 | 0.007381 |
| hsa04020 | Calcium signaling pathway | 10 | 0.007381 |
| hsa04080 | Neuroactive ligand-receptor interaction | 12 | 0.007381 |
| hsa04260 | Cardiac muscle contraction | 6 | 0.007381 |
| hsa04022 | cGMP-PKG signaling pathway | 8 | 0.007381 |
| hsa05414 | Dilated cardiomyopathy | 6 | 0.009376 |
| hsa04261 | Adrenergic signaling in cardiomyocytes | 7 | 0.015342 |
| hsa04024 | cAMP signaling pathway | 8 | 0.027206 |
| hsa04911 | Insulin secretion | 5 | 0.027206 |
| hsa04970 | Salivary secretion | 5 | 0.03461 |
| hsa04514 | Cell adhesion molecules | 6 | 0.049845 |

Supplementary Table 6. GSEA results of patients with MUC16 mutation

| Ontology | Description | SIZE | NES | NOM p value | FDR q value |
| --- | --- | --- | --- | --- | --- |
| GOBP | REGULATION_OF_TELOMERASE_ACTIVITY | 47 | 2.299577 | 0 | 0.0125536 |
| GOBP | NCRNA_METABOLIC_PROCESS | 492 | 2.276037 | 0 | 0.0110933 |
| GOBP | POSITIVE_REGULATION_OF_TELOMERASE_ACTIVITY | 33 | 2.270006 | 0 | 0.007943 |
| GOBP | NADH_METABOLIC_PROCESS | 43 | 2.253434 | 0 | 0.0095339 |
| GOBP | NCRNA_PROCESSING | 400 | 2.246543 | 0.001968504 | 0.0103355 |
| GOBP | PURINE_NUCLEOSIDE_MONOPHOSPHATE_BIOSYNTHETIC_PROCESS | 23 | 2.218687 | 0 | 0.0175463 |
| GOBP | MITOTIC_NUCLEAR_DIVISION | 296 | 2.217387 | 0 | 0.0154755 |
| GOBP | MITOTIC_SISTER_CHROMATID_SEGREGATION | 164 | 2.203819 | 0 | 0.0175728 |
| GOBP | GLUCOSE_CATABOLIC_PROCESS | 36 | 2.195031 | 0 | 0.016945 |
| GOBP | SISTER_CHROMATID_SEGREGATION | 199 | 2.188817 | 0 | 0.0172042 |
| GOBP | TRNA_METABOLIC_PROCESS | 179 | 2.180866 | 0.001988072 | 0.0180122 |
| GOBP | NUCLEOBASE_METABOLIC_PROCESS | 34 | 2.180186 | 0 | 0.0166618 |
| GOBP | RRNA_METABOLIC_PROCESS | 238 | 2.17897 | 0.003976143 | 0.0156617 |
| GOBP | REGULATION_OF_TELOMERE_MAINTENANCE_VIA_TELOMERE_LENGTHENING | 60 | 2.174411 | 0.002118644 | 0.0159419 |
| GOBP | MONOSACCHARIDE_CATABOLIC_PROCESS | 62 | 2.170612 | 0 | 0.0157318 |
| GOBP | MATURATION_OF_5_8S_RRNA | 35 | 2.158869 | 0 | 0.0165078 |
| GOBP | NEGATIVE_REGULATION_OF_NUCLEAR_DIVISION | 54 | 2.153709 | 0.00209205 | 0.0170453 |
| GOBP | ATTACHMENT_OF_SPINDLE_MICROTUBULES_TO_KINETOCHORE | 35 | 2.150986 | 0 | 0.0169099 |
| GOBP | MITOTIC_METAPHASE_PLATE_CONGRESSION | 50 | 2.146382 | 0 | 0.0170943 |
| GOBP | RIBOSOME_BIOGENESIS | 307 | 2.1396 | 0.005952381 | 0.0180616 |
| GOBP | POSITIVE_REGULATION_OF_TELOMERE_MAINTENANCE_VIA_TELOMERE_LENGTHENING | 36 | 2.138935 | 0.002079002 | 0.0173405 |
| GOBP | NUCLEOBASE_BIOSYNTHETIC_PROCESS | 18 | 2.138127 | 0 | 0.0168651 |
| GOBP | PYRUVATE_METABOLIC_PROCESS | 150 | 2.133891 | 0 | 0.0172953 |
| GOBP | REGULATION_OF_CHROMOSOME_SEGREGATION | 88 | 2.133088 | 0 | 0.0167738 |
| GOBP | NUCLEIC_ACID_PHOSPHODIESTER_BOND_HYDROLYSIS | 296 | 2.130005 | 0 | 0.0167112 |
| GOBP | TELOMERE_ORGANIZATION | 172 | 2.129754 | 0 | 0.0161296 |
| GOBP | RNA_MODIFICATION | 169 | 2.129353 | 0.003976143 | 0.0156693 |
| GOBP | REGULATION_OF_CHROMOSOME_SEPARATION | 71 | 2.128726 | 0.002066116 | 0.0152913 |
| GOBP | MULTI_ORGANISM_LOCALIZATION | 66 | 2.12756 | 0 | 0.0149451 |
| GOBP | NAD_METABOLIC_PROCESS | 51 | 2.125765 | 0 | 0.0147617 |
| GOBP | REGULATION_OF_MITOTIC_SISTER_CHROMATID_SEGREGATION | 45 | 2.124781 | 0 | 0.0144583 |
| GOBP | SISTER_CHROMATID_COHESION | 62 | 2.122342 | 0 | 0.0144758 |
| GOBP | REGULATION_OF_SPINDLE_ASSEMBLY | 25 | 2.120006 | 0.001988072 | 0.0145347 |
| GOBP | CHROMOSOME_SEGREGATION | 335 | 2.120002 | 0 | 0.0141072 |
| GOBP | CHROMOSOME_SEPARATION | 95 | 2.119871 | 0.002070393 | 0.0137303 |
| GOBP | TRNA_MODIFICATION | 89 | 2.117459 | 0.004040404 | 0.0138174 |
| GOBP | RNA_PHOSPHODIESTER_BOND_HYDROLYSIS | 154 | 2.111457 | 0 | 0.0149425 |
| GOBP | DNA_DEPENDENT_DNA_REPLICATION | 157 | 2.108688 | 0 | 0.0148531 |
| GOBP | REGULATION_OF_MITOTIC_NUCLEAR_DIVISION | 109 | 2.10744 | 0.004149378 | 0.0148633 |
| GOBP | REGULATION_OF_NUCLEAR_DIVISION | 134 | 2.105332 | 0.002123142 | 0.0150814 |
| GOBP | RNA_DEPENDENT_DNA_BIOSYNTHETIC_PROCESS | 70 | 2.104902 | 0.002087683 | 0.0147816 |
| GOBP | DNA_REPLICATION | 280 | 2.104785 | 0 | 0.0144296 |
| GOBP | GLYCOLYTIC_PROCESS_THROUGH_FRUCTOSE_6_PHOSPHATE | 29 | 2.104202 | 0 | 0.0141609 |
| GOBP | TRNA_PROCESSING | 130 | 2.102793 | 0.003913894 | 0.0140645 |
| GOBP | NUCLEAR_CHROMOSOME_SEGREGATION | 271 | 2.100113 | 0.002141328 | 0.0142904 |
| GOBP | AMINO_ACID_ACTIVATION | 49 | 2.099896 | 0 | 0.0139797 |
| GOBP | NUCLEOSIDE_MONOPHOSPHATE_BIOSYNTHETIC_PROCESS | 43 | 2.097955 | 0 | 0.0139615 |
| GOBP | RNA_EXPORT_FROM_NUCLEUS | 142 | 2.096196 | 0.002053388 | 0.0138747 |
| GOBP | ORGANELLE_FISSION | 482 | 2.093729 | 0 | 0.014037 |
| GOBP | METAPHASE_ANAPHASE_TRANSITION_OF_CELL_CYCLE | 64 | 2.091727 | 0.002079002 | 0.0141043 |
| GOBP | TELOMERE_MAINTENANCE_VIA_TELOMERE_LENGTHENING | 80 | 2.090481 | 0.00210084 | 0.0141776 |
| GOBP | CHROMOSOME_LOCALIZATION | 80 | 2.086215 | 0.002118644 | 0.015006 |
| GOBP | CELLULAR_COMPONENT_DISASSEMBLY_INVOLVED_IN_EXECUTION_PHASE_OF_APOPTOSIS | 34 | 2.086109 | 0 | 0.0147394 |
| GOBP | RNA_LOCALIZATION | 233 | 2.084577 | 0.00203666 | 0.0147192 |
| GOBP | INTERSTRAND_CROSS_LINK_REPAIR | 57 | 2.083574 | 0 | 0.0146284 |
| GOBP | RNA_PHOSPHODIESTER_BOND_HYDROLYSIS_ENDONUCLEOLYTIC | 78 | 2.08143 | 0.001992032 | 0.0150052 |
| GOBP | DNA_BIOSYNTHETIC_PROCESS | 189 | 2.079797 | 0 | 0.0150226 |
| GOBP | NEGATIVE_REGULATION_OF_DNA_REPAIR | 36 | 2.079058 | 0 | 0.014828 |
| GOBP | REGULATION_OF_TELOMERE_MAINTENANCE | 79 | 2.078257 | 0.002096436 | 0.0147287 |
| GOBP | RIBONUCLEOPROTEIN_COMPLEX_BIOGENESIS | 470 | 2.076659 | 0.007905139 | 0.0146479 |
| GOBP | MATURATION_OF_SSU_RRNA | 50 | 2.070731 | 0.00203252 | 0.0154453 |
| GOBP | POSITIVE_REGULATION_OF_TELOMERE_MAINTENANCE | 51 | 2.070456 | 0.002087683 | 0.0152197 |
| GOBP | GLYCOLIPID_CATABOLIC_PROCESS | 16 | 2.070308 | 0 | 0.0150066 |
| GOBP | PIGMENT_BIOSYNTHETIC_PROCESS | 57 | 2.070082 | 0.00203252 | 0.0148448 |
| GOBP | METAPHASE_PLATE_CONGRESSION | 64 | 2.069768 | 0.00204499 | 0.0147257 |
| GOBP | MICROTUBULE_CYTOSKELETON_ORGANIZATION_INVOLVED_IN_MITOSIS | 142 | 2.069719 | 0.004024145 | 0.0145026 |
| GOBP | MITOCHONDRIAL_RNA_METABOLIC_PROCESS | 47 | 2.068195 | 0.003898636 | 0.0144798 |
| GOBP | MATURATION_OF_LSU_RRNA | 28 | 2.061136 | 0 | 0.0154645 |
| GOBP | NUCLEAR_EXPORT | 201 | 2.059156 | 0 | 0.0157065 |
| GOBP | MITOTIC_SPINDLE_ASSEMBLY | 65 | 2.058682 | 0.004032258 | 0.0155576 |
| GOBP | CARBOHYDRATE_CATABOLIC_PROCESS | 197 | 2.058552 | 0 | 0.0153698 |
| GOBP | RIBONUCLEOSIDE_MONOPHOSPHATE_BIOSYNTHETIC_PROCESS | 34 | 2.052123 | 0 | 0.0164072 |
| GOBP | ESTABLISHMENT_OF_MITOTIC_SPINDLE_LOCALIZATION | 33 | 2.05088 | 0.002061856 | 0.0164206 |
| GOBP | MATURATION_OF_5_8S_RRNA_FROM_TRICISTRONIC_RRNA_TRANSCRIPT_SSU_RRNA_5_8S_RRNA_LSU_RRNA | 24 | 2.049462 | 0 | 0.0164988 |
| GOBP | RNA_PHOSPHODIESTER_BOND_HYDROLYSIS_EXONUCLEOLYTIC | 43 | 2.047385 | 0.002028398 | 0.0168804 |
| GOBP | LONG_CHAIN_FATTY_ACID_IMPORT_INTO_CELL | 17 | 2.047271 | 0.002028398 | 0.0166678 |
| GOBP | ESTABLISHMENT_OF_RNA_LOCALIZATION | 200 | 2.043419 | 0.004132231 | 0.0172841 |
| GOBP | CENTROMERE_COMPLEX_ASSEMBLY | 56 | 2.041739 | 0.004065041 | 0.0174998 |
| GOBP | MICROTUBULE_ORGANIZING_CENTER_LOCALIZATION | 30 | 2.034666 | 0 | 0.0191173 |
| GOBP | CYTOPLASMIC_TRANSLATIONAL_INITIATION | 34 | 2.031773 | 0 | 0.0196093 |
| GOBP | PROTEIN_LOCALIZATION_TO_CHROMOSOME_CENTROMERIC_REGION | 25 | 2.031453 | 0 | 0.0194233 |
| GOBP | MRNA_EXPORT_FROM_NUCLEUS | 113 | 2.029689 | 0.002061856 | 0.019608 |
| GOBP | TRANSLESION_SYNTHESIS | 42 | 2.028596 | 0 | 0.019659 |
| GOBP | BRANCHED_CHAIN_AMINO_ACID_METABOLIC_PROCESS | 23 | 2.028149 | 0.002004008 | 0.0195639 |
| GOBP | ESTABLISHMENT_OF_MITOTIC_SPINDLE_ORIENTATION | 28 | 2.027726 | 0.002087683 | 0.0194126 |
| GOBP | TRNA_WOBBLE_BASE_MODIFICATION | 21 | 2.027391 | 0 | 0.0192659 |
| GOBP | TETRAPYRROLE_METABOLIC_PROCESS | 62 | 2.027214 | 0.002083333 | 0.0190947 |
| GOBP | CLEAVAGE_INVOLVED_IN_RRNA_PROCESSING | 27 | 2.025821 | 0 | 0.019222 |
| GOBP | KINETOCHORE_ORGANIZATION | 22 | 2.025168 | 0 | 0.0191398 |
| GOBP | SPINDLE_ORGANIZATION | 180 | 2.024519 | 0.00617284 | 0.0190379 |
| GOBP | WATER_SOLUBLE_VITAMIN_METABOLIC_PROCESS | 84 | 2.024415 | 0 | 0.0188366 |
| GOBP | POSITIVE_REGULATION_OF_VIRAL_GENOME_REPLICATION | 31 | 2.024218 | 0 | 0.0186887 |
| GOBP | REGULATION_OF_TRANSLATIONAL_FIDELITY | 20 | 2.023818 | 0.001984127 | 0.0185921 |
| GOBP | NCRNA_CATABOLIC_PROCESS | 37 | 2.019398 | 0 | 0.0192022 |
| GOBP | NEGATIVE_REGULATION_OF_CHROMOSOME_ORGANIZATION | 91 | 2.017638 | 0.006382979 | 0.0194828 |
| GOBP | NCRNA_3_END_PROCESSING | 50 | 2.017607 | 0.005988024 | 0.0192798 |
| GOBP | SPINDLE_ASSEMBLY | 113 | 2.015181 | 0.006289308 | 0.0196488 |
| GOBP | REGULATION_OF_DNA_BIOSYNTHETIC_PROCESS | 107 | 2.014579 | 0.002083333 | 0.019522 |
| GOBP | RNA_3_END_PROCESSING | 153 | 2.013971 | 0 | 0.0194386 |
| GOBP | REGULATION_OF_SPINDLE_CHECKPOINT | 15 | 2.011561 | 0.002008032 | 0.0196496 |
| GOBP | RNA_METHYLATION | 84 | 2.011484 | 0.008097166 | 0.0194706 |
| GOBP | SPINDLE_LOCALIZATION | 52 | 2.011413 | 0 | 0.0193087 |
| GOBP | PROTEIN_LOCALIZATION_TO_CHROMOSOME | 91 | 2.009935 | 0.004149378 | 0.0194097 |
| GOBP | ORGANELLE_INHERITANCE | 15 | 2.007072 | 0.003913894 | 0.0199522 |
| GOBP | NEGATIVE_REGULATION_OF_DOUBLE_STRAND_BREAK_REPAIR_VIA_HOMOLOGOUS_RECOMBINATION | 19 | 2.005587 | 0 | 0.0201069 |
| GOBP | CARBOHYDRATE_PHOSPHORYLATION | 24 | 2.004512 | 0.00204918 | 0.0201864 |
| GOBP | DNA_SYNTHESIS_INVOLVED_IN_DNA_REPAIR | 53 | 2.00407 | 0 | 0.0200912 |
| GOBP | MITOCHONDRIAL_RNA_PROCESSING | 17 | 2.002499 | 0.00203252 | 0.0202808 |
| GOBP | ATTACHMENT_OF_MITOTIC_SPINDLE_MICROTUBULES_TO_KINETOCHORE | 15 | 2.00138 | 0 | 0.0203787 |
| GOBP | NEGATIVE_REGULATION_OF_METAPHASE_ANAPHASE_TRANSITION_OF_CELL_CYCLE | 41 | 2.001325 | 0.006198347 | 0.0202179 |
| GOBP | MRNA_TRANSPORT | 151 | 2.000819 | 0.002083333 | 0.0202349 |
| GOBP | MITOTIC_SPINDLE_ORGANIZATION | 117 | 2.000795 | 0.006198347 | 0.0200542 |
| GOBP | NUCLEOBASE_CONTAINING_COMPOUND_TRANSPORT | 252 | 1.999862 | 0.004166667 | 0.0201623 |
| GOBP | CHROMOSOME_CONDENSATION | 46 | 1.99951 | 0.004338395 | 0.0200698 |
| GOBP | DNA_REPLICATION_CHECKPOINT | 17 | 1.998586 | 0 | 0.0203333 |
| GOBP | RECOMBINATIONAL_REPAIR | 139 | 1.997947 | 0.004115226 | 0.0203188 |
| GOBP | TRNA_TRANSPORT | 37 | 1.996944 | 0 | 0.0204121 |
| GOBP | SIGNAL_TRANSDUCTION_INVOLVED_IN_CELL_CYCLE_CHECKPOINT | 75 | 1.996788 | 0.00204918 | 0.0202849 |
| GOBP | DNA_LIGATION | 15 | 1.995705 | 0.002004008 | 0.0204037 |
| GOBP | MITOTIC_CYTOKINESIS | 70 | 1.995145 | 0 | 0.0203351 |
| GOBP | CELL_CYCLE_G2_M_PHASE_TRANSITION | 272 | 1.993449 | 0.004024145 | 0.0206417 |
| GOBP | ATP_DEPENDENT_CHROMATIN_REMODELING | 89 | 1.992375 | 0.006198347 | 0.0207567 |
| GOBP | PROTEIN_TARGETING_TO_MITOCHONDRION | 100 | 1.990752 | 0.003853565 | 0.021022 |
| GOBP | NCRNA_EXPORT_FROM_NUCLEUS | 38 | 1.990187 | 0.002105263 | 0.0209763 |
| GOBP | UBIQUINONE_METABOLIC_PROCESS | 18 | 1.988732 | 0 | 0.0211322 |
| GOBP | NUCLEOTIDE_PHOSPHORYLATION | 133 | 1.988587 | 0 | 0.0209813 |
| GOBP | RIBOSOMAL_SMALL_SUBUNIT_BIOGENESIS | 75 | 1.98612 | 0.005905512 | 0.0213849 |
| GOBP | POSITIVE_REGULATION_OF_TRANSCRIPTION_BY_RNA_POLYMERASE_I | 23 | 1.986044 | 0 | 0.0212331 |
| GOBP | DOUBLE_STRAND_BREAK_REPAIR | 266 | 1.980177 | 0.006185567 | 0.0225312 |
| GOBP | RESPONSE_TO_STEROL_DEPLETION | 17 | 1.979734 | 0 | 0.022521 |
| GOBP | CERAMIDE_CATABOLIC_PROCESS | 21 | 1.978833 | 0 | 0.0225836 |
| GOBP | DIOL_BIOSYNTHETIC_PROCESS | 22 | 1.976683 | 0.006036217 | 0.0228789 |
| GOBP | MITOCHONDRIAL_GENE_EXPRESSION | 165 | 1.976298 | 0.00967118 | 0.0228162 |
| GOBP | REGULATION_OF_NUCLEASE_ACTIVITY | 22 | 1.976252 | 0 | 0.0226459 |
| GOBP | PTERIDINE_CONTAINING_COMPOUND_METABOLIC_PROCESS | 33 | 1.975179 | 0.002070393 | 0.0228429 |
| GOBP | RRNA_CONTAINING_RIBONUCLEOPROTEIN_COMPLEX_EXPORT_FROM_NUCLEUS | 15 | 1.974128 | 0 | 0.0229149 |
| GOBP | RETROGRADE_VESICLE_MEDIATED_TRANSPORT_GOLGI_TO_ENDOPLASMIC_RETICULUM | 87 | 1.973328 | 0.002079002 | 0.0229206 |
| GOBP | REGULATION_OF_MITOTIC_SPINDLE_ASSEMBLY | 18 | 1.972392 | 0.004056795 | 0.0229546 |
| GOBP | FORMATION_OF_EXTRACHROMOSOMAL_CIRCULAR_DNA | 15 | 1.97223 | 0.002136752 | 0.0228363 |
| GOBP | PURINE_NUCLEOBASE_METABOLIC_PROCESS | 19 | 1.970604 | 0.002118644 | 0.0230154 |
| GOBP | TETRAPYRROLE_BIOSYNTHETIC_PROCESS | 28 | 1.97049 | 0.002028398 | 0.022895 |
| GOBP | CHROMATIN_REMODELING_AT_CENTROMERE | 47 | 1.970101 | 0.01010101 | 0.0228812 |
| GOBP | SNRNA_METABOLIC_PROCESS | 47 | 1.969163 | 0.004024145 | 0.0229665 |
| GOBP | POSTREPLICATION_REPAIR | 52 | 1.968918 | 0 | 0.0228563 |
| GOBP | MITOTIC_CELL_CYCLE_CHECKPOINT | 158 | 1.968055 | 0.00408998 | 0.0229421 |
| GOBP | REGULATION_OF_DNA_REPLICATION | 107 | 1.967559 | 0 | 0.0229865 |
| GOBP | MEMBRANE_LIPID_CATABOLIC_PROCESS | 37 | 1.965898 | 0 | 0.0232972 |
| GOBP | EXIT_FROM_MITOSIS | 28 | 1.965063 | 0.00409836 | 0.0234029 |
| GOBP | REGULATION_OF_EXIT_FROM_MITOSIS | 16 | 1.964031 | 0.003976143 | 0.0235031 |
| GOBP | ERROR_FREE_TRANSLESION_SYNTHESIS | 22 | 1.963416 | 0.001980198 | 0.02352 |
| GOBP | CENTROSOME_SEPARATION | 15 | 1.962606 | 0.003960396 | 0.023571 |
| GOBP | PROTEIN_LOCALIZATION_TO_KINETOCHORE | 19 | 1.961063 | 0 | 0.0239283 |
| GOBP | REGULATION_OF_CHROMOSOME_ORGANIZATION | 273 | 1.961025 | 0.008368201 | 0.0237719 |
| GOBP | PYRIMIDINE_NUCLEOBASE_METABOLIC_PROCESS | 16 | 1.958163 | 0.002114165 | 0.0244673 |
| GOBP | CELL_CYCLE_CHECKPOINT | 208 | 1.95234 | 0.006198347 | 0.0259091 |
| GOBP | REGULATION_OF_CENTROSOME_CYCLE | 47 | 1.950982 | 0.004140787 | 0.0260989 |
| GOBP | POSITIVE_REGULATION_OF_DNA_BIOSYNTHETIC_PROCESS | 65 | 1.950973 | 0.00621118 | 0.0259327 |
| GOBP | MICROTUBULE_ORGANIZING_CENTER_ORGANIZATION | 139 | 1.950715 | 0.010351967 | 0.0258289 |
| GOBP | DNA_REPLICATION_INDEPENDENT_NUCLEOSOME_ORGANIZATION | 54 | 1.950667 | 0.01632653 | 0.0256819 |
| GOBP | REGULATION_OF_CELL_CYCLE_G2_M_PHASE_TRANSITION | 215 | 1.950614 | 0.008064516 | 0.0255318 |
| GOBP | CELL_CYCLE_DNA_REPLICATION | 64 | 1.949605 | 0.002053388 | 0.02565 |
| GOBP | RRNA_MODIFICATION | 40 | 1.946774 | 0.00814664 | 0.0263582 |
| GOBP | TELOMERE_MAINTENANCE_VIA_SEMI_CONSERVATIVE_REPLICATION | 27 | 1.946449 | 0 | 0.0263082 |
| GOBP | CELLULAR_RESPONSE_TO_ARSENIC_CONTAINING_SUBSTANCE | 18 | 1.942531 | 0.002016129 | 0.0272038 |
| GOBP | RNA_CATABOLIC_PROCESS | 414 | 1.939826 | 0.004065041 | 0.0277122 |
| GOBP | NEGATIVE_REGULATION_OF_RESPONSE_TO_DNA_DAMAGE_STIMULUS | 82 | 1.938612 | 0 | 0.0278512 |
| GOBP | MRNA_MODIFICATION | 25 | 1.938109 | 0.00210084 | 0.0278566 |
| GOBP | ESTABLISHMENT_OF_SPINDLE_ORIENTATION | 34 | 1.938068 | 0.004081633 | 0.0277345 |
| GOBP | REGULATION_OF_DOUBLE_STRAND_BREAK_REPAIR_VIA_HOMOLOGOUS_RECOMBINATION | 47 | 1.93802 | 0.010615711 | 0.0275837 |
| GOBP | RIG_I_SIGNALING_PATHWAY | 25 | 1.935878 | 0.00610998 | 0.0279325 |
| GOBP | MITOCHONDRIAL_CALCIUM_ION_TRANSMEMBRANE_TRANSPORT | 25 | 1.935857 | 0.0041841 | 0.0277741 |
| GOBP | RIBOSOMAL_LARGE_SUBUNIT_BIOGENESIS | 72 | 1.9322 | 0.011627907 | 0.0286791 |
| GOBP | REGULATION_OF_DNA_DEPENDENT_DNA_REPLICATION | 47 | 1.931937 | 0.00209205 | 0.0285834 |
| GOBP | CELLULAR_AMINO_ACID_METABOLIC_PROCESS | 330 | 1.930116 | 0.00409836 | 0.0289476 |
| GOBP | REGULATION_OF_ATP_METABOLIC_PROCESS | 118 | 1.929756 | 0.00203252 | 0.0289368 |
| GOBP | RRNA_CATABOLIC_PROCESS | 20 | 1.928636 | 0.003968254 | 0.0290774 |
| GOBP | NEGATIVE_REGULATION_OF_TELOMERE_MAINTENANCE_VIA_TELOMERE_LENGTHENING | 27 | 1.926946 | 0.014767933 | 0.0294826 |
| GOBP | KINETOCHORE_ASSEMBLY | 17 | 1.926447 | 0.004008016 | 0.0294039 |
| GOBP | POSITIVE_REGULATION_OF_DNA_METABOLIC_PROCESS | 198 | 1.926332 | 0.006276151 | 0.0292659 |
| GOBP | DNA_INTEGRITY_CHECKPOINT | 155 | 1.926254 | 0.012219959 | 0.0291154 |
| GOBP | REPLICATION_FORK_PROCESSING | 40 | 1.926122 | 0.010526316 | 0.0290083 |
| GOBP | INTRA_S_DNA_DAMAGE_CHECKPOINT | 17 | 1.925516 | 0 | 0.0289955 |
| GOBP | ORGANELLE_DISASSEMBLY | 107 | 1.923742 | 0.001972387 | 0.0294338 |
| GOBP | REGULATION_OF_DOUBLE_STRAND_BREAK_REPAIR | 86 | 1.923596 | 0.004282655 | 0.0293167 |
| GOBP | NEGATIVE_REGULATION_OF_TELOMERE_MAINTENANCE_VIA_TELOMERASE | 20 | 1.922561 | 0.004246285 | 0.0294743 |
| GOBP | ANAPHASE_PROMOTING_COMPLEX_DEPENDENT_CATABOLIC_PROCESS | 83 | 1.919563 | 0.017681729 | 0.0302313 |
| GOBP | POSITIVE_REGULATION_OF_TYPE_I_INTERFERON_PRODUCTION | 77 | 1.919277 | 0.004123712 | 0.0301667 |
| GOBP | CENTROSOME_DUPLICATION | 71 | 1.918927 | 0.008350731 | 0.0301397 |
| GOBP | MATURATION_OF_SSU_RRNA_FROM_TRICISTRONIC_RRNA_TRANSCRIPT_SSU_RRNA_5_8S_RRNA_LSU_RRNA | 37 | 1.917436 | 0.006072875 | 0.030395 |
| GOBP | DNA_DAMAGE_RESPONSE_DETECTION_OF_DNA_DAMAGE | 39 | 1.91701 | 0.00409836 | 0.0303513 |
| GOBP | PROTEIN_LOCALIZATION_TO_MITOCHONDRION | 146 | 1.915816 | 0.005825243 | 0.0305266 |
| GOBP | N_TERMINAL_PROTEIN_AMINO_ACID_MODIFICATION | 29 | 1.915623 | 0.002183406 | 0.0304341 |
| GOBP | TELOMERASE_RNA_LOCALIZATION | 19 | 1.914356 | 0.00407332 | 0.0306821 |
| GOBP | DNA_RECOMBINATION | 291 | 1.914117 | 0.014522822 | 0.0306183 |
| GOBP | ENDONUCLEOLYTIC_CLEAVAGE_INVOLVED_IN_RRNA_PROCESSING | 15 | 1.914115 | 0.003976143 | 0.0304613 |
| GOBP | RNA_DECAPPING | 16 | 1.913519 | 0.004040404 | 0.0305138 |
| GOBP | REGULATION_OF_DNA_REPAIR | 130 | 1.913294 | 0.010460251 | 0.0304554 |
| GOBP | APOPTOTIC_DNA_FRAGMENTATION | 23 | 1.912374 | 0.002028398 | 0.0305562 |
| GOBP | REGULATION_OF_UBIQUITIN_PROTEIN_TRANSFERASE_ACTIVITY | 53 | 1.911564 | 0 | 0.0307285 |
| GOBP | POSITIVE_REGULATION_OF_UBIQUITIN_PROTEIN_TRANSFERASE_ACTIVITY | 34 | 1.91008 | 0.004158004 | 0.0311037 |
| GOBP | MRNA_3_END_PROCESSING | 99 | 1.909496 | 0.008230452 | 0.0311569 |
| GOBP | REGULATION_OF_DNA_METABOLIC_PROCESS | 349 | 1.908801 | 0.004219409 | 0.0311994 |
| GOBP | VIRAL_TRANSLATION | 16 | 1.906715 | 0.003898636 | 0.031705 |
| GOBP | MITOCHONDRIAL_TRANSPORT | 270 | 1.90638 | 0.003898636 | 0.0317006 |
| GOBP | SNRNA_PROCESSING | 36 | 1.906254 | 0.001988072 | 0.0315883 |
| GOBP | TRNA_METHYLATION | 40 | 1.90539 | 0.008130081 | 0.0317005 |
| GOBP | DNA_CONFORMATION_CHANGE | 349 | 1.903698 | 0.026369167 | 0.0321757 |
| GOBP | TRANSLATIONAL_ELONGATION | 134 | 1.898605 | 0.015873017 | 0.0338456 |
| GOBP | CELLULAR_RESPONSE_TO_STEROL_DEPLETION | 15 | 1.896737 | 0 | 0.0343347 |
| GOBP | REGULATION_OF_TYPE_I_INTERFERON_MEDIATED_SIGNALING_PATHWAY | 34 | 1.896574 | 0.006072875 | 0.0342118 |
| GOBP | FOLIC_ACID_CONTAINING_COMPOUND_METABOLIC_PROCESS | 27 | 1.896077 | 0.004175365 | 0.0341889 |
| GOBP | POSITIVE_REGULATION_OF_TRANSLATION | 133 | 1.895478 | 0 | 0.0342512 |
| GOBP | MATURATION_OF_LSU_RRNA_FROM_TRICISTRONIC_RRNA_TRANSCRIPT_SSU_RRNA_5_8S_RRNA_LSU_RRNA | 15 | 1.894187 | 0.001968504 | 0.0344527 |
| GOBP | MITOTIC_CHROMOSOME_CONDENSATION | 15 | 1.894152 | 0 | 0.0342954 |
| GOBP | DNA_CATABOLIC_PROCESS | 39 | 1.892229 | 0.002074689 | 0.0348357 |
| GOBP | DNA_DEPENDENT_DNA_REPLICATION_MAINTENANCE_OF_FIDELITY | 49 | 1.891338 | 0.010638298 | 0.0349459 |
| GOBP | MEMBRANE_DISASSEMBLY | 15 | 1.889831 | 0.00610998 | 0.0353539 |
| GOBP | TETRAHYDROFOLATE_METABOLIC_PROCESS | 19 | 1.888541 | 0.00408998 | 0.0356004 |
| GOBP | NEGATIVE_REGULATION_OF_CELL_CYCLE_PHASE_TRANSITION | 257 | 1.887622 | 0.006160164 | 0.035757 |
| GOBP | REGULATION_OF_UBIQUITIN_PROTEIN_LIGASE_ACTIVITY | 23 | 1.887584 | 0.010141988 | 0.0356097 |
| GOBP | LIPID_IMPORT_INTO_CELL | 34 | 1.886019 | 0.004545454 | 0.0360638 |
| GOBP | POSITIVE_REGULATION_OF_CHROMOSOME_SEPARATION | 17 | 1.88273 | 0.002145923 | 0.0370918 |
| GOBP | TRICARBOXYLIC_ACID_CYCLE | 34 | 1.880703 | 0.00407332 | 0.0377503 |
| GOBP | POSITIVE_REGULATION_OF_CELL_CYCLE_PROCESS | 299 | 1.880286 | 0.004301075 | 0.03772 |
| GOBP | NUCLEOTIDE_EXCISION_REPAIR_DNA_INCISION | 39 | 1.880277 | 0.005976096 | 0.037556 |
| GOBP | BASE_EXCISION_REPAIR | 39 | 1.880196 | 0.010245902 | 0.0373992 |
| GOBP | SIGNAL_TRANSDUCTION_BY_P53_CLASS_MEDIATOR | 262 | 1.879847 | 0.004081633 | 0.0373482 |
| GOBP | DNA_GEOMETRIC_CHANGE | 114 | 1.878829 | 0.020876827 | 0.0375732 |
| GOBP | DNA_REPLICATION_INITIATION | 40 | 1.87812 | 0.004115226 | 0.0376045 |
| GOBP | NUCLEOTIDE_EXCISION_REPAIR_DNA_GAP_FILLING | 23 | 1.878055 | 0.005905512 | 0.0374646 |
| GOBP | REGULATION_OF_CYTOKINESIS | 90 | 1.876633 | 0.010288066 | 0.0378497 |
| GOBP | NCRNA_TRANSCRIPTION | 108 | 1.875889 | 0.016393442 | 0.0379242 |
| GOBP | COBALAMIN_METABOLIC_PROCESS | 22 | 1.874407 | 0.002141328 | 0.0383125 |
| GOBP | POSITIVE_REGULATION_OF_METAPHASE_ANAPHASE_TRANSITION_OF_CELL_CYCLE | 15 | 1.874376 | 0.004166667 | 0.0381563 |
| GOBP | CYTOPLASMIC_PATTERN_RECOGNITION_RECEPTOR_SIGNALING_PATHWAY_IN_RESPONSE_TO_VIRUS | 32 | 1.874073 | 0 | 0.0380991 |
| GOBP | CYTOKINESIS | 171 | 1.873298 | 0.006198347 | 0.0381842 |
| GOBP | REGULATION_OF_CARBOHYDRATE_CATABOLIC_PROCESS | 90 | 1.871321 | 0.002070393 | 0.0387501 |
| GOBP | PURINE_NUCLEOSIDE_MONOPHOSPHATE_METABOLIC_PROCESS | 43 | 1.871247 | 0 | 0.0386172 |
| GOBP | NEGATIVE_REGULATION_OF_CELL_CYCLE_G2_M_PHASE_TRANSITION | 108 | 1.870615 | 0.012170386 | 0.0387127 |
| GOBP | PIGMENT_METABOLIC_PROCESS | 72 | 1.869832 | 0.002114165 | 0.0388657 |
| GOBP | NUCLEOSIDE_DIPHOSPHATE_METABOLIC_PROCESS | 156 | 1.868804 | 0 | 0.0390761 |
| GOBP | G2_DNA_DAMAGE_CHECKPOINT | 35 | 1.867555 | 0.004065041 | 0.0393902 |
| GOBP | HISTONE_EXCHANGE | 58 | 1.866713 | 0.024691358 | 0.039596 |
| GOBP | NUCLEAR_TRANSPORT | 343 | 1.864808 | 0.010548524 | 0.0401956 |
| GOBP | REGULATION_OF_RIG_I_SIGNALING_PATHWAY | 19 | 1.864197 | 0.002074689 | 0.0402314 |
| GOBP | POSITIVE_REGULATION_OF_CELLULAR_AMIDE_METABOLIC_PROCESS | 163 | 1.863827 | 0 | 0.0402225 |
| GOBP | CENTRIOLE_ASSEMBLY | 45 | 1.862111 | 0.012658228 | 0.0406542 |
| GOBP | MITOTIC_SISTER_CHROMATID_COHESION | 27 | 1.861667 | 0.012903226 | 0.0407002 |
| GOBP | POSITIVE_REGULATION_OF_DNA_REPAIR | 74 | 1.861587 | 0.006451613 | 0.04056 |
| GOBP | REGULATION_OF_CELL_CYCLE_PHASE_TRANSITION | 465 | 1.861571 | 0.01039501 | 0.0404053 |
| GOBP | NEGATIVE_REGULATION_OF_MRNA_PROCESSING | 29 | 1.860126 | 0.006060606 | 0.0409494 |
| GOBP | ISOPRENOID_BIOSYNTHETIC_PROCESS | 28 | 1.859557 | 0.004210526 | 0.0410042 |
| GOBP | REGULATION_OF_MRNA_CATABOLIC_PROCESS | 210 | 1.858154 | 0.004201681 | 0.041415 |
| GOBP | LACTATE_METABOLIC_PROCESS | 15 | 1.858032 | 0.00209205 | 0.0413214 |
| GOBP | REGULATION_OF_SPINDLE_ORGANIZATION | 39 | 1.857918 | 0.004219409 | 0.041194 |
| GOBP | NUCLEOSIDE_MONOPHOSPHATE_METABOLIC_PROCESS | 75 | 1.857205 | 0.004166667 | 0.0413553 |
| GOBP | POSITIVE_REGULATION_OF_PROTEIN_EXPORT_FROM_NUCLEUS | 20 | 1.856417 | 0.004081633 | 0.0414758 |
| GOBP | RNA_POLYADENYLATION | 46 | 1.856369 | 0.010373444 | 0.0413413 |
| GOBP | MITOCHONDRIAL_TRANSLATION | 134 | 1.854742 | 0.027667984 | 0.0418086 |
| GOBP | CYTOKINETIC_PROCESS | 38 | 1.854345 | 0.003976143 | 0.0417776 |
| GOBP | FORMATION_OF_CYTOPLASMIC_TRANSLATION_INITIATION_COMPLEX | 16 | 1.852199 | 0.001926782 | 0.0425869 |
| GOBP | UBIQUITIN_DEPENDENT_ERAD_PATHWAY | 79 | 1.851874 | 0.004282655 | 0.0425646 |
| GOBP | HISTONE_MRNA_METABOLIC_PROCESS | 24 | 1.851215 | 0.012295082 | 0.0426028 |
| GOBP | NEGATIVE_REGULATION_OF_CELL_CYCLE_PROCESS | 346 | 1.850001 | 0.010438413 | 0.0428753 |
| GOBP | RNA_5_END_PROCESSING | 23 | 1.849521 | 0.011904762 | 0.0429218 |
| GOBP | GLUCOSE_6_PHOSPHATE_METABOLIC_PROCESS | 25 | 1.849512 | 0.004219409 | 0.0427634 |
| GOBP | RESPONSE_TO_INTERLEUKIN_12 | 50 | 1.849455 | 0.009784736 | 0.042631 |
| GOBP | ENDOPLASMIC_RETICULUM_ORGANIZATION | 86 | 1.849289 | 0.003992016 | 0.0425439 |
| GOBP | CELL_DIFFERENTIATION_INVOLVED_IN_EMBRYONIC_PLACENTA_DEVELOPMENT | 23 | 1.849278 | 0.006085193 | 0.0423858 |
| GOBP | REGULATION_OF_RESPONSE_TO_DNA_DAMAGE_STIMULUS | 220 | 1.849119 | 0.012552301 | 0.0422884 |
| GOBP | ATP_METABOLIC_PROCESS | 312 | 1.848648 | 0.015296367 | 0.0422756 |
| GOBP | POSITIVE_REGULATION_OF_TELOMERE_CAPPING | 17 | 1.847791 | 0.008298756 | 0.0424552 |
| GOBP | HEME_METABOLIC_PROCESS | 33 | 1.846924 | 0.014056225 | 0.0426192 |
| GOBP | POSITIVE_REGULATION_OF_DNA_TEMPLATED_TRANSCRIPTION_ELONGATION | 26 | 1.846472 | 0.02244898 | 0.0426191 |
| GOBP | REGULATION_OF_TELOMERE_CAPPING | 26 | 1.845894 | 0.020618556 | 0.0427344 |
| GOBP | ESTABLISHMENT_OF_ORGANELLE_LOCALIZATION | 429 | 1.845211 | 0 | 0.0428406 |
| GOBP | RESPONSE_TO_X_RAY | 32 | 1.838631 | 0.004264392 | 0.0454427 |
| GOBP | MEIOTIC_CELL_CYCLE | 246 | 1.837516 | 0.013363029 | 0.0457552 |
| GOBP | REGULATION_OF_TRANSLATION_IN_RESPONSE_TO_STRESS | 21 | 1.837227 | 0.005976096 | 0.0457169 |
| GOBP | NUCLEOTIDE_EXCISION_REPAIR | 107 | 1.837198 | 0.014736842 | 0.0455615 |
| GOBP | RIBONUCLEOPROTEIN_COMPLEX_SUBUNIT_ORGANIZATION | 228 | 1.836627 | 0.016161617 | 0.0456487 |
| GOBP | RESPONSE_TO_IONIZING_RADIATION | 142 | 1.833266 | 0.010460251 | 0.0468569 |
| GOBP | REGULATION_OF_PROTEIN_EXPORT_FROM_NUCLEUS | 32 | 1.831742 | 0.012422361 | 0.0473512 |
| GOBP | FOLIC_ACID_METABOLIC_PROCESS | 20 | 1.831133 | 0.006382979 | 0.0474232 |
| GOBP | DNA_UNWINDING_INVOLVED_IN_DNA_REPLICATION | 16 | 1.830365 | 0.012121212 | 0.0475355 |
| GOBP | MITOTIC_G1_S_TRANSITION_CHECKPOINT | 63 | 1.829733 | 0.006224067 | 0.047637 |
| GOBP | RNA_SURVEILLANCE | 15 | 1.828915 | 0.007874016 | 0.0478679 |
| GOBP | MAINTENANCE_OF_PROTEIN_LOCALIZATION_IN_ORGANELLE | 42 | 1.82831 | 0.01 | 0.0479591 |
| GOBP | HISTONE_PHOSPHORYLATION | 39 | 1.828288 | 0.008333334 | 0.0478021 |
| GOBP | NEGATIVE_REGULATION_OF_MITOTIC_CELL_CYCLE | 311 | 1.827849 | 0.014522822 | 0.0478638 |
| GOBP | CYTOSKELETON_DEPENDENT_CYTOKINESIS | 98 | 1.827422 | 0.010460251 | 0.0478463 |
| GOBP | PROTEIN_QUALITY_CONTROL_FOR_MISFOLDED_OR_INCOMPLETELY_SYNTHESIZED_PROTEINS | 28 | 1.826519 | 0.00996016 | 0.0480219 |
| GOBP | REGULATION_OF_VIRAL_INDUCED_CYTOPLASMIC_PATTERN_RECOGNITION_RECEPTOR_SIGNALING_PATHWAY | 23 | 1.826421 | 0.00210084 | 0.0478848 |
| GOBP | REGULATION_OF_CYCLIN_DEPENDENT_PROTEIN_KINASE_ACTIVITY | 102 | 1.825458 | 0.006302521 | 0.0481246 |
| GOBP | REGULATION_OF_CELLULAR_RESPIRATION | 22 | 1.824312 | 0.004291846 | 0.0484071 |
| GOBP | RIBOSOME_ASSEMBLY | 64 | 1.824252 | 0.02661597 | 0.0482576 |
| GOBP | REGULATION_OF_CELL_CYCLE_CHECKPOINT | 31 | 1.822922 | 0.021008404 | 0.048647 |
| GOBP | NUCLEAR_TRANSCRIBED_MRNA_CATABOLIC_PROCESS_EXONUCLEOLYTIC | 35 | 1.822435 | 0.016032064 | 0.0487024 |
| GOBP | MITOTIC_DNA_INTEGRITY_CHECKPOINT | 105 | 1.821353 | 0.018404908 | 0.0489297 |
| GOBP | MITOCHONDRIAL_TRANSMEMBRANE_TRANSPORT | 109 | 1.821135 | 0.012024048 | 0.0488535 |
| GOBP | NUCLEOSIDE_PHOSPHATE_BIOSYNTHETIC_PROCESS | 265 | 1.821059 | 0.002083333 | 0.0487304 |
| GOBP | NEURON_DEATH_IN_RESPONSE_TO_OXIDATIVE_STRESS | 26 | 1.820475 | 0.010638298 | 0.0487916 |
| GOBP | PROTEIN_MONOUBIQUITINATION | 66 | 1.817252 | 0.012711864 | 0.0500179 |
| GOBP | CELL_CYCLE_G1_S_PHASE_TRANSITION | 288 | 1.816946 | 0.004273505 | 0.0499756 |
| GOBP | ERROR_PRONE_TRANSLESION_SYNTHESIS | 21 | 1.812974 | 0.019379845 | 0.0515575 |
| GOBP | POSITIVE_REGULATION_OF_DOUBLE_STRAND_BREAK_REPAIR | 41 | 1.81218 | 0.014705882 | 0.0517527 |
| GOBP | SIGNAL_TRANSDUCTION_IN_RESPONSE_TO_DNA_DAMAGE | 130 | 1.811971 | 0.010162601 | 0.0516501 |
| GOBP | RESPONSE_TO_UV | 146 | 1.807277 | 0.006382979 | 0.0535643 |
| GOBP | TERMINATION_OF_RNA_POLYMERASE_II_TRANSCRIPTION | 35 | 1.80672 | 0.018072288 | 0.0537093 |
| GOBP | ERAD_PATHWAY | 101 | 1.805859 | 0.004201681 | 0.0538375 |
| GOBP | ESTABLISHMENT_OF_PROTEIN_LOCALIZATION_TO_TELOMERE | 18 | 1.805574 | 0.01004016 | 0.0538314 |
| GOBP | NUCLEUS_ORGANIZATION | 123 | 1.803839 | 0.016632017 | 0.0544021 |
| GOBP | RRNA_METHYLATION | 28 | 1.803256 | 0.018595042 | 0.0544951 |
| GOBP | VIRAL_GENE_EXPRESSION | 198 | 1.802448 | 0.028462999 | 0.0547126 |
| GOBP | REGULATION_OF_SIGNAL_TRANSDUCTION_BY_P53_CLASS_MEDIATOR | 176 | 1.802192 | 0.014285714 | 0.054629 |
| GOBP | ER_NUCLEUS_SIGNALING_PATHWAY | 52 | 1.801742 | 0.0041841 | 0.0546631 |
| GOBP | PORE_COMPLEX_ASSEMBLY | 20 | 1.801507 | 0.020325202 | 0.0546138 |
| GOBP | PROTEIN_DNA_COMPLEX_SUBUNIT_ORGANIZATION | 277 | 1.800017 | 0.03526971 | 0.0551578 |
| GOBP | REGULATION_OF_NUCLEOTIDE_METABOLIC_PROCESS | 120 | 1.799198 | 0.008333334 | 0.0553236 |
| GOBP | MACROMOLECULE_METHYLATION | 300 | 1.796696 | 0.010799136 | 0.0563093 |
| GOBP | TYPE_I_INTERFERON_PRODUCTION | 128 | 1.794153 | 0.016064256 | 0.0573259 |
| GOBP | REGULATION_OF_CHOLESTEROL_BIOSYNTHETIC_PROCESS | 48 | 1.793801 | 0.008791209 | 0.0573065 |
| GOBP | MEMBRANE_DOCKING | 180 | 1.79269 | 0.01039501 | 0.057675 |
| GOBP | REGULATION_OF_TRANSCRIPTION_INVOLVED_IN_G1_S_TRANSITION_OF_MITOTIC_CELL_CYCLE | 35 | 1.791974 | 0.010683761 | 0.0578636 |
| GOBP | CELL_DEATH_IN_RESPONSE_TO_OXIDATIVE_STRESS | 80 | 1.791061 | 0.00867679 | 0.0580912 |
| GOBP | SNRNA_TRANSCRIPTION | 75 | 1.790953 | 0.022727273 | 0.0579576 |
| GOBP | REGULATION_OF_TRANSLATIONAL_INITIATION_IN_RESPONSE_TO_STRESS | 15 | 1.789447 | 0.006097561 | 0.0585766 |
| GOBP | REGULATION_OF_CHOLESTEROL_METABOLIC_PROCESS | 61 | 1.789142 | 0.012631579 | 0.0585281 |
| GOBP | REGULATION_OF_MRNA_METABOLIC_PROCESS | 334 | 1.787852 | 0.014644352 | 0.0589989 |
| GOBP | TRANSCRIPTION_COUPLED_NUCLEOTIDE_EXCISION_REPAIR | 73 | 1.787834 | 0.018556701 | 0.0588248 |
| GOBP | GLUTAMINE_METABOLIC_PROCESS | 23 | 1.787414 | 0.01875 | 0.0588669 |
| GOBP | POSITIVE_REGULATION_OF_CHROMOSOME_ORGANIZATION | 164 | 1.786665 | 0.014736842 | 0.0591075 |
| GOBP | POSITIVE_REGULATION_OF_CELL_CYCLE | 391 | 1.78656 | 0.010706638 | 0.0589811 |
| GOBP | DNA_TEMPLATED_TRANSCRIPTION_ELONGATION | 111 | 1.785653 | 0.020325202 | 0.0592684 |
| GOBP | UBIQUITIN_DEPENDENT_PROTEIN_CATABOLIC_PROCESS_VIA_THE_MULTIVESICULAR_BODY_SORTING_PATHWAY | 23 | 1.785305 | 0.013888889 | 0.059246 |
| GOBP | NADP_METABOLIC_PROCESS | 35 | 1.783462 | 0.016771488 | 0.0599582 |
| GOBP | RIBONUCLEOSIDE_MONOPHOSPHATE_METABOLIC_PROCESS | 56 | 1.783405 | 0.00209205 | 0.0598154 |
| GOBP | MITOCHONDRIAL_MEMBRANE_ORGANIZATION | 139 | 1.782396 | 0.01372549 | 0.0601441 |
| GOBP | PROTEIN_AUTOPROCESSING | 24 | 1.782064 | 0.008438818 | 0.0601247 |
| GOBP | TELOMERE_MAINTENANCE_VIA_RECOMBINATION | 15 | 1.781482 | 0.020833334 | 0.060287 |
| GOBP | RIBOSOMAL_LARGE_SUBUNIT_ASSEMBLY | 28 | 1.780596 | 0.02661597 | 0.0606224 |
| GOBP | DNA_DAMAGE_RESPONSE_SIGNAL_TRANSDUCTION_BY_P53_CLASS_MEDIATOR | 106 | 1.780455 | 0.012048192 | 0.0604896 |
| GOBP | POSITIVE_REGULATION_OF_NUCLEAR_DIVISION | 56 | 1.779975 | 0.008658009 | 0.0605247 |
| GOBP | PSEUDOURIDINE_SYNTHESIS | 18 | 1.779401 | 0.021782178 | 0.0606324 |
| GOBP | VESICLE_DOCKING_INVOLVED_IN_EXOCYTOSIS | 44 | 1.777197 | 0.010351967 | 0.0614646 |
| GOBP | PROTEIN_CONTAINING_COMPLEX_LOCALIZATION | 278 | 1.777182 | 0.012847966 | 0.0612995 |
| GOBP | DNA_PACKAGING | 237 | 1.776182 | 0.03909465 | 0.0617063 |
| GOBP | PORPHYRIN_CONTAINING_COMPOUND_METABOLIC_PROCESS | 40 | 1.775945 | 0.018108651 | 0.0616441 |
| GOBP | NEGATIVE_REGULATION_OF_DNA_METABOLIC_PROCESS | 123 | 1.7759 | 0.019148936 | 0.0614794 |
| GOBP | ENDOPLASMIC_RETICULUM_TO_CYTOSOL_TRANSPORT | 28 | 1.775308 | 0.018907564 | 0.0616253 |
| GOBP | CHROMOSOME_ORGANIZATION_INVOLVED_IN_MEIOTIC_CELL_CYCLE | 64 | 1.775301 | 0.01754386 | 0.0614545 |
| GOBP | DNA_TEMPLATED_TRANSCRIPTION_TERMINATION | 72 | 1.775054 | 0.032128513 | 0.0614272 |
| GOBP | NEGATIVE_REGULATION_OF_MRNA_METABOLIC_PROCESS | 88 | 1.774809 | 0.015283843 | 0.0613727 |
| GOBP | REGULATION_OF_AUTOPHAGY_OF_MITOCHONDRION | 31 | 1.773414 | 0.00998004 | 0.0619285 |
| GOBP | PROTEIN_MODIFICATION_BY_SMALL_PROTEIN_REMOVAL | 300 | 1.772223 | 0.006342495 | 0.062392 |
| GOBP | COPII_COATED_VESICLE_BUDDING | 73 | 1.771974 | 0.010245902 | 0.0623505 |
| GOBP | ACROSOME_ASSEMBLY | 16 | 1.771646 | 0.006479482 | 0.0623371 |
| GOBP | RRNA_TRANSCRIPTION | 32 | 1.770206 | 0.014675053 | 0.0628408 |
| GOBP | POSITIVE_REGULATION_OF_TELOMERASE_RNA_LOCALIZATION_TO_CAJAL_BODY | 15 | 1.76847 | 0.018181818 | 0.0635752 |
| GOBP | NEGATIVE_REGULATION_OF_PROTEASOMAL_UBIQUITIN_DEPENDENT_PROTEIN_CATABOLIC_PROCESS | 35 | 1.768322 | 0.012631579 | 0.0634754 |
| GOBP | PROTEIN_PHOSPHOPANTETHEINYLATION | 331 | 1.767521 | 0.00212766 | 0.0637019 |
| GOBP | INTRINSIC_APOPTOTIC_SIGNALING_PATHWAY_IN_RESPONSE_TO_OXIDATIVE_STRESS | 44 | 1.766311 | 0.008333334 | 0.0641345 |
| GOBP | REGULATION_OF_TRANSCRIPTION_BY_RNA_POLYMERASE_I | 33 | 1.766255 | 0.01443299 | 0.0639753 |
| GOBP | POSITIVE_REGULATION_OF_VIRAL_PROCESS | 91 | 1.765636 | 0.004016064 | 0.0641197 |
| GOBP | DICARBOXYLIC_ACID_METABOLIC_PROCESS | 96 | 1.765085 | 0.00862069 | 0.0641867 |
| GOBP | NON_RECOMBINATIONAL_REPAIR | 106 | 1.764305 | 0.026804123 | 0.0644244 |
| GOBP | REGULATION_OF_PROTEIN_EXIT_FROM_ENDOPLASMIC_RETICULUM | 27 | 1.762855 | 0.01039501 | 0.0650284 |
| GOBP | NEGATIVE_REGULATION_OF_UBIQUITIN_DEPENDENT_PROTEIN_CATABOLIC_PROCESS | 48 | 1.761811 | 0.010416667 | 0.0654058 |
| GOBP | ACTIVATION_OF_NF_KAPPAB_INDUCING_KINASE_ACTIVITY | 18 | 1.760164 | 0.022821577 | 0.0660901 |
| GOBP | TRANSLATIONAL_TERMINATION | 105 | 1.759729 | 0.04819277 | 0.0661887 |
| GOBP | NEGATIVE_REGULATION_OF_MRNA_SPLICING_VIA_SPLICEOSOME | 20 | 1.758838 | 0.021868788 | 0.0664124 |
| GOBP | POSITIVE_REGULATION_OF_DNA_REPLICATION | 37 | 1.758183 | 0.006342495 | 0.0665747 |
| GOBP | TOXIN_TRANSPORT | 39 | 1.757046 | 0.012847966 | 0.0670091 |
| GOBP | CELLULAR_RESPONSE_TO_CHEMICAL_STRESS | 337 | 1.756704 | 0 | 0.0669963 |
| GOBP | MEIOTIC_CELL_CYCLE_PROCESS | 186 | 1.756344 | 0.019736841 | 0.0670708 |
| GOBP | TRANSCRIPTION_BY_RNA_POLYMERASE_I | 65 | 1.756186 | 0.034343433 | 0.0669517 |
| GOBP | REGULATION_OF_DNA_RECOMBINATION | 104 | 1.756106 | 0.035416666 | 0.0668173 |
| GOBP | REGULATION_OF_CELLULAR_RESPONSE_TO_HEAT | 79 | 1.754888 | 0.027659575 | 0.0673164 |
| GOBP | FATTY_ACID_CATABOLIC_PROCESS | 106 | 1.753982 | 0.020449897 | 0.0675912 |
| GOBP | NADPH_REGENERATION | 18 | 1.751859 | 0.020746889 | 0.0685653 |
| GOBP | AEROBIC_RESPIRATION | 86 | 1.749877 | 0.034548946 | 0.0694258 |
| GOBP | POSITIVE_REGULATION_OF_GLUCONEOGENESIS | 15 | 1.749715 | 0.015053763 | 0.06932 |
| GOBP | RESPONSE_TO_MISFOLDED_PROTEIN | 24 | 1.749333 | 0.01629328 | 0.0693935 |
| GOBP | ACTIVATION_OF_INNATE_IMMUNE_RESPONSE | 145 | 1.749021 | 0.016096579 | 0.069433 |
| GOBP | CELL_REDOX_HOMEOSTASIS | 43 | 1.748324 | 0.014056225 | 0.0696474 |
| GOBP | DNA_CATABOLIC_PROCESS_ENDONUCLEOLYTIC | 30 | 1.746112 | 0.008179959 | 0.0706522 |
| GOBP | POSITIVE_REGULATION_OF_CYTOKINESIS | 40 | 1.744515 | 0.013274336 | 0.0713577 |
| GOBP | METHYLATION | 353 | 1.74428 | 0.017167382 | 0.0713107 |
| GOBP | PROTEIN_IMPORT | 194 | 1.743675 | 0.008583691 | 0.0714657 |
| GOBP | NEGATIVE_REGULATION_OF_TELOMERE_MAINTENANCE | 35 | 1.743119 | 0.042283297 | 0.071635 |
| GOBP | REGULATION_OF_CELLULAR_AMIDE_METABOLIC_PROCESS | 478 | 1.742963 | 0.004310345 | 0.0715463 |
| GOBP | ENDOPLASMIC_RETICULUM_TO_GOLGI_VESICLE_MEDIATED_TRANSPORT | 206 | 1.74287 | 0.014522822 | 0.0714032 |
| GOBP | DEOXYRIBONUCLEOSIDE_TRIPHOSPHATE_METABOLIC_PROCESS | 16 | 1.742338 | 0.037475344 | 0.0715302 |
| GOBP | DEOXYRIBONUCLEOTIDE_METABOLIC_PROCESS | 40 | 1.741914 | 0.026262626 | 0.0715686 |
| GOBP | VITAMIN_METABOLIC_PROCESS | 130 | 1.738925 | 0.010964912 | 0.0730692 |
| GOBP | REGULATION_OF_CENTRIOLE_REPLICATION | 21 | 1.738853 | 0.016877636 | 0.0729184 |
| GOBP | REGULATION_OF_ENDOPLASMIC_RETICULUM_UNFOLDED_PROTEIN_RESPONSE | 29 | 1.73837 | 0.014736842 | 0.0730026 |
| GOBP | NUCLEOLAR_LARGE_RRNA_TRANSCRIPTION_BY_RNA_POLYMERASE_I | 17 | 1.737846 | 0.010080645 | 0.0730628 |
| GOBP | PROTEIN_LOCALIZATION_TO_CHROMATIN | 29 | 1.736216 | 0.016460905 | 0.0737717 |
| GOBP | RESPONSE_TO_ANTIBIOTIC | 43 | 1.734226 | 0.010309278 | 0.0746188 |
| GOBP | GOLGI_VESICLE_TRANSPORT | 372 | 1.734167 | 0.006276151 | 0.0744706 |
| GOBP | REGULATION_OF_TRANSLATIONAL_INITIATION | 80 | 1.733827 | 0.010504202 | 0.0744704 |
| GOBP | MITOCHONDRIAL_TRANSLATIONAL_TERMINATION | 89 | 1.733823 | 0.047904193 | 0.0742944 |
| GOBP | CRISTAE_FORMATION | 35 | 1.733182 | 0.03468208 | 0.0744636 |
| GOBP | ACETYL_COA_BIOSYNTHETIC_PROCESS | 22 | 1.732832 | 0.024793388 | 0.0744438 |
| GOBP | PEPTIDYL_ASPARAGINE_MODIFICATION | 31 | 1.732229 | 0.014893618 | 0.0745737 |
| GOBP | NEGATIVE_REGULATION_OF_HISTONE_ACETYLATION | 16 | 1.731757 | 0.012219959 | 0.0746767 |
| GOBP | NUCLEOTIDE_EXCISION_REPAIR_PREINCISION_COMPLEX_ASSEMBLY | 29 | 1.731437 | 0.038306452 | 0.0747126 |
| GOBP | POSITIVE_REGULATION_OF_GENE_EXPRESSION_EPIGENETIC | 55 | 1.730768 | 0.025641026 | 0.0749245 |
| GOBP | TRANSCRIPTION_ELONGATION_FROM_RNA_POLYMERASE_II_PROMOTER | 85 | 1.730317 | 0.020449897 | 0.0750445 |
| GOBP | NEGATIVE_REGULATION_OF_ATP_METABOLIC_PROCESS | 28 | 1.729094 | 0.004405286 | 0.0755306 |
| GOBP | ALCOHOL_BIOSYNTHETIC_PROCESS | 156 | 1.728967 | 0.010940919 | 0.0754545 |
| GOBP | PROTEIN_TRANSMEMBRANE_IMPORT_INTO_INTRACELLULAR_ORGANELLE | 36 | 1.728862 | 0.024242423 | 0.075312 |
| GOBP | PURINE_CONTAINING_COMPOUND_METABOLIC_PROCESS | 457 | 1.728193 | 0.00203666 | 0.0755977 |
| GOBP | REGULATION_OF_DNA_TEMPLATED_TRANSCRIPTION_IN_RESPONSE_TO_STRESS | 115 | 1.727807 | 0.016528925 | 0.0756573 |
| GOBP | DNA_STRAND_ELONGATION_INVOLVED_IN_DNA_REPLICATION | 19 | 1.726717 | 0.012269938 | 0.0760636 |
| GOBP | ASPARTATE_FAMILY_AMINO_ACID_METABOLIC_PROCESS | 50 | 1.72593 | 0.008602151 | 0.0763985 |
| GOBP | PROTEIN_FOLDING | 231 | 1.725382 | 0.010373444 | 0.0764616 |
| GOBP | MICROVILLUS_ASSEMBLY | 16 | 1.725302 | 0.023404256 | 0.0763248 |
| GOBP | INNATE_IMMUNE_RESPONSE_ACTIVATING_SIGNAL_TRANSDUCTION | 117 | 1.725281 | 0.020449897 | 0.07617 |
| GOBP | REGULATION_OF_TRANSCRIPTION_ELONGATION_FROM_RNA_POLYMERASE_II_PROMOTER | 30 | 1.724293 | 0.024539877 | 0.076557 |
| GOBP | PURINE_CONTAINING_COMPOUND_SALVAGE | 15 | 1.723883 | 0.01992032 | 0.0766257 |
| GOBP | POSITIVE_REGULATION_OF_MITOTIC_NUCLEAR_DIVISION | 43 | 1.723608 | 0.013129103 | 0.0765919 |
| GOBP | PROTEIN_TRANSMEMBRANE_TRANSPORT | 60 | 1.722413 | 0.014344262 | 0.077122 |
| GOBP | RNA_SPLICING_VIA_TRANSESTERIFICATION_REACTIONS | 383 | 1.721819 | 0.04024145 | 0.0772453 |
| GOBP | CILIARY_BASAL_BODY_PLASMA_MEMBRANE_DOCKING | 94 | 1.721516 | 0.049281314 | 0.0772894 |
| GOBP | OLIGOSACCHARIDE_LIPID_INTERMEDIATE_BIOSYNTHETIC_PROCESS | 21 | 1.720279 | 0.01863354 | 0.0778038 |
| GOBP | ANTIGEN_PROCESSING_AND_PRESENTATION_OF_PEPTIDE_ANTIGEN | 192 | 1.720253 | 0.032945737 | 0.0776311 |
| GOBP | REGULATION_OF_DOUBLE_STRAND_BREAK_REPAIR_VIA_NONHOMOLOGOUS_END_JOINING | 28 | 1.717707 | 0.01898734 | 0.078835 |
| GOBP | REGULATION_OF_MITOCHONDRIAL_GENE_EXPRESSION | 29 | 1.717577 | 0.034816246 | 0.0787299 |
| GOBP | POSITIVE_REGULATION_OF_CELL_CYCLE_ARREST | 81 | 1.717456 | 0.010438413 | 0.0786091 |
| GOBP | ANTIGEN_PROCESSING_AND_PRESENTATION_OF_PEPTIDE_ANTIGEN_VIA_MHC_CLASS_I | 99 | 1.717376 | 0.044921875 | 0.078482 |
| GOBP | SULFUR_AMINO_ACID_METABOLIC_PROCESS | 37 | 1.717325 | 0.015283843 | 0.0783159 |
| GOBP | CELLULAR_PROTEIN_COMPLEX_DISASSEMBLY | 221 | 1.717107 | 0.030927835 | 0.0782807 |
| GOBP | MONOSACCHARIDE_BIOSYNTHETIC_PROCESS | 97 | 1.716228 | 0.018828452 | 0.0786293 |
| GOBP | REGULATION_OF_RAC_PROTEIN_SIGNAL_TRANSDUCTION | 18 | 1.716094 | 0.014989293 | 0.078545 |
| GOBP | CELLULAR_METABOLIC_COMPOUND_SALVAGE | 34 | 1.715892 | 0.018442623 | 0.078489 |
| GOBP | PROTEASOMAL_PROTEIN_CATABOLIC_PROCESS | 481 | 1.715403 | 0.008247423 | 0.0786546 |
| GOBP | NUCLEOTIDE_EXCISION_REPAIR_PREINCISION_COMPLEX_STABILIZATION | 21 | 1.714913 | 0.028985508 | 0.0787941 |
| GOBP | MIRNA_METABOLIC_PROCESS | 29 | 1.714687 | 0.023913043 | 0.0787282 |
| GOBP | VESICLE_TARGETING_TO_FROM_OR_WITHIN_GOLGI | 74 | 1.714181 | 0.028455285 | 0.0788573 |
| GOBP | REGULATION_OF_GENERATION_OF_PRECURSOR_METABOLITES_AND_ENERGY | 160 | 1.714035 | 0.006263048 | 0.0787253 |
| GOBP | AMP_METABOLIC_PROCESS | 16 | 1.713224 | 0.02016129 | 0.0789747 |
| GOBP | REGULATION_OF_OXIDATIVE_STRESS_INDUCED_CELL_DEATH | 59 | 1.713069 | 0.017429193 | 0.0789047 |
| GOBP | REGULATION_OF_GENE_SILENCING_BY_RNA | 122 | 1.712748 | 0.036324788 | 0.0789131 |
| GOBP | POSITIVE_REGULATION_OF_TRANSLATIONAL_INITIATION | 30 | 1.71215 | 0.023305085 | 0.0790705 |
| GOBP | POSITIVE_REGULATION_OF_PROTEIN_LOCALIZATION_TO_NUCLEUS | 85 | 1.710095 | 0.012847966 | 0.0800642 |
| GOBP | POSITIVE_REGULATION_OF_MRNA_METABOLIC_PROCESS | 84 | 1.709969 | 0.030172413 | 0.0799701 |
| GOBP | NUCLEOSIDE_BISPHOSPHATE_BIOSYNTHETIC_PROCESS | 67 | 1.709696 | 0.01871102 | 0.0799315 |
| GOBP | PTERIDINE_CONTAINING_COMPOUND_BIOSYNTHETIC_PROCESS | 15 | 1.70952 | 0.023210831 | 0.0798776 |
| GOBP | NEGATIVE_REGULATION_OF_DNA_DEPENDENT_DNA_REPLICATION | 17 | 1.709314 | 0.01871102 | 0.0797997 |
| GOBP | TAIL_ANCHORED_MEMBRANE_PROTEIN_INSERTION_INTO_ER_MEMBRANE | 16 | 1.70694 | 0.029069768 | 0.08105 |
| GOBP | BLASTOCYST_GROWTH | 19 | 1.706589 | 0.030927835 | 0.0810835 |
| GOBP | NUCLEAR_TRANSCRIBED_MRNA_CATABOLIC_PROCESS | 208 | 1.706497 | 0.04109589 | 0.0809724 |
| GOBP | RESPONSE_TO_GAMMA_RADIATION | 53 | 1.706427 | 0.020661157 | 0.0808538 |
| GOBP | PROTEIN_EXIT_FROM_ENDOPLASMIC_RETICULUM | 47 | 1.7055 | 0.021097047 | 0.0812203 |
| GOBP | POSITIVE_REGULATION_OF_PROTEIN_TARGETING_TO_MITOCHONDRION | 32 | 1.701955 | 0.018556701 | 0.0831873 |
| GOBP | POSITIVE_REGULATION_OF_LYMPHOCYTE_CHEMOTAXIS | 20 | 1.701746 | 0.028282829 | 0.0831441 |
| GOBP | POSITIVE_REGULATION_OF_MITOCHONDRIAL_TRANSLATION | 16 | 1.701479 | 0.03076923 | 0.0831098 |
| GOBP | PURINE_CONTAINING_COMPOUND_BIOSYNTHETIC_PROCESS | 206 | 1.700873 | 0.014314928 | 0.0832861 |
| GOBP | REGULATION_OF_SISTER_CHROMATID_COHESION | 22 | 1.70076 | 0.04077253 | 0.083187 |
| GOBP | CELLULAR_PROTEIN_CONTAINING_COMPLEX_LOCALIZATION | 20 | 1.700697 | 0.021786492 | 0.0830596 |
| GOBP | ACETYL_COA_BIOSYNTHETIC_PROCESS_FROM_PYRUVATE | 15 | 1.698836 | 0.036734693 | 0.0839785 |
| GOBP | DIOL_METABOLIC_PROCESS | 29 | 1.698399 | 0.018036073 | 0.0840777 |
| GOBP | DNA_STRAND_ELONGATION | 26 | 1.698291 | 0.024691358 | 0.0839501 |
| GOBP | NEGATIVE_REGULATION_OF_RNA_SPLICING | 25 | 1.697429 | 0.037401576 | 0.0843278 |
| GOBP | MITOCHONDRIAL_CALCIUM_ION_HOMEOSTASIS | 26 | 1.697348 | 0.009009009 | 0.0841876 |
| GOBP | NUCLEAR_ENVELOPE_REASSEMBLY | 18 | 1.696228 | 0.037328094 | 0.0847113 |
| GOBP | POSITIVE_REGULATION_OF_CELL_CYCLE_PHASE_TRANSITION | 105 | 1.695787 | 0.033333335 | 0.0847873 |
| GOBP | INTRINSIC_APOPTOTIC_SIGNALING_PATHWAY_IN_RESPONSE_TO_DNA_DAMAGE_BY_P53_CLASS_MEDIATOR | 44 | 1.694375 | 0.030737706 | 0.085507 |
| GOBP | N_TERMINAL_PROTEIN_AMINO_ACID_ACETYLATION | 17 | 1.691973 | 0.029850746 | 0.0869439 |
| GOBP | POSITIVE_REGULATION_OF_RESPONSE_TO_DNA_DAMAGE_STIMULUS | 107 | 1.691843 | 0.03526971 | 0.0868505 |
| GOBP | POSITIVE_REGULATION_OF_INTERFERON_BETA_PRODUCTION | 33 | 1.691412 | 0.020242915 | 0.0869206 |
| GOBP | PROTEIN_LOCALIZATION_TO_NUCLEUS | 275 | 1.691055 | 0.023109244 | 0.0869402 |
| GOBP | REGULATION_OF_GENE_SILENCING | 141 | 1.689938 | 0.036796536 | 0.087501 |
| GOBP | POSITIVE_REGULATION_OF_ESTABLISHMENT_OF_PROTEIN_LOCALIZATION | 327 | 1.6895 | 0 | 0.0876054 |
| GOBP | ALPHA_AMINO_ACID_BIOSYNTHETIC_PROCESS | 67 | 1.68851 | 0.022633744 | 0.0879663 |
| GOBP | CELLULAR_RESPONSE_TO_VIRUS | 66 | 1.688157 | 0.02004008 | 0.08801 |
| GOBP | RNA_SPLICING | 477 | 1.688139 | 0.046653144 | 0.0878397 |
| GOBP | MITOTIC_CYTOKINETIC_PROCESS | 23 | 1.687794 | 0.026052104 | 0.0878674 |
| GOBP | MRNA_METHYLATION | 16 | 1.687614 | 0.02742616 | 0.0877901 |
| GOBP | RNA_CAPPING | 34 | 1.687287 | 0.04048583 | 0.0878147 |
| GOBP | POSITIVE_REGULATION_OF_DOUBLE_STRAND_BREAK_REPAIR_VIA_NONHOMOLOGOUS_END_JOINING | 16 | 1.687007 | 0.025586354 | 0.0878377 |
| GOBP | REGULATION_OF_OXIDATIVE_STRESS_INDUCED_NEURON_DEATH | 22 | 1.686189 | 0.027956989 | 0.0881979 |
| GOBP | REGULATION_OF_TRANSCRIPTION_FROM_RNA_POLYMERASE_II_PROMOTER_IN_RESPONSE_TO_HYPOXIA | 77 | 1.683459 | 0.0498008 | 0.0897202 |
| GOBP | CELLULAR_RESPONSE_TO_HEAT | 119 | 1.678628 | 0.03125 | 0.092445 |
| GOBP | NEGATIVE_REGULATION_OF_PROTEIN_ACETYLATION | 23 | 1.678499 | 0.028747434 | 0.0923358 |
| GOBP | VIRAL_LIFE_CYCLE | 348 | 1.677907 | 0.02020202 | 0.0925708 |
| GOBP | CELLULAR_RESPONSE_TO_REACTIVE_NITROGEN_SPECIES | 17 | 1.677375 | 0.013215859 | 0.0927343 |
| GOBP | RNA_DESTABILIZATION | 36 | 1.674175 | 0.03726708 | 0.094647 |
| GOBP | NEGATIVE_REGULATION_OF_DNA_RECOMBINATION | 44 | 1.674098 | 0.04496788 | 0.0944885 |
| GOBP | RESPONSE_TO_ENDOPLASMIC_RETICULUM_STRESS | 295 | 1.673534 | 0.020876827 | 0.094682 |
| GOBP | NUCLEAR_TRANSCRIBED_MRNA_CATABOLIC_PROCESS_DEADENYLATION_DEPENDENT_DECAY | 77 | 1.672845 | 0.03411514 | 0.094971 |
| GOBP | ALPHA_AMINO_ACID_METABOLIC_PROCESS | 190 | 1.670923 | 0.010799136 | 0.0960829 |
| GOBP | RESPONSE_TO_LEUKEMIA_INHIBITORY_FACTOR | 80 | 1.669957 | 0.01927195 | 0.0962933 |
| GOBP | REGULATION_OF_SMALL_MOLECULE_METABOLIC_PROCESS | 434 | 1.668382 | 0 | 0.097171 |
| GOBP | VESICLE_BUDDING_FROM_MEMBRANE | 109 | 1.668125 | 0.03206413 | 0.0971896 |
| GOBP | ESTABLISHMENT_OF_PROTEIN_LOCALIZATION_TO_PLASMA_MEMBRANE | 58 | 1.667785 | 0.012448133 | 0.097222 |
| GOBP | 2_OXOGLUTARATE_METABOLIC_PROCESS | 16 | 1.667563 | 0.026639344 | 0.0972058 |
| GOBP | REGULATION_OF_MRNA_3_END_PROCESSING | 28 | 1.667341 | 0.045548655 | 0.0971379 |
| GOBP | METHIONINE_METABOLIC_PROCESS | 16 | 1.667193 | 0.01826484 | 0.0970274 |
| GOBP | FATTY_ACID_BETA_OXIDATION | 75 | 1.667134 | 0.03420523 | 0.0968698 |
| GOBP | MITOTIC_G2_M_TRANSITION_CHECKPOINT | 36 | 1.666758 | 0.037656903 | 0.0969429 |
| GOBP | POSITIVE_REGULATION_OF_MRNA_PROCESSING | 33 | 1.666177 | 0.046121594 | 0.0971653 |
| GOBP | REGULATION_OF_CELL_CYCLE_ARREST | 107 | 1.665874 | 0.024691358 | 0.097143 |
| GOBP | DNA_METHYLATION | 72 | 1.665405 | 0.027600849 | 0.0973294 |
| GOBP | NEGATIVE_REGULATION_OF_PEPTIDYL_LYSINE_ACETYLATION | 19 | 1.665375 | 0.022540983 | 0.0971518 |
| GOBP | NEGATIVE_REGULATION_OF_RNA_CATABOLIC_PROCESS | 70 | 1.665124 | 0.022123894 | 0.0971372 |
| GOBP | PROTEIN_N_LINKED_GLYCOSYLATION | 74 | 1.661256 | 0.045738045 | 0.0995256 |
| GOBP | SMALL_MOLECULE_CATABOLIC_PROCESS | 429 | 1.660696 | 0.010526316 | 0.0997585 |
| GOBP | MAINTENANCE_OF_PROTEIN_LOCATION_IN_CELL | 64 | 1.660429 | 0.014314928 | 0.0997662 |
| GOBP | STEROL_BIOSYNTHETIC_PROCESS | 78 | 1.659878 | 0.032822758 | 0.099931 |
| GOBP | DEOXYRIBONUCLEOTIDE_BIOSYNTHETIC_PROCESS | 15 | 1.659833 | 0.042944785 | 0.0997533 |
| GOBP | CELLULAR_RESPONSE_TO_HYDROGEN_PEROXIDE | 84 | 1.659127 | 0.016701462 | 0.1000262 |
| GOBP | PEPTIDYL_LYSINE_DIMETHYLATION | 22 | 1.658989 | 0.044585988 | 0.0999481 |
| GOBP | RIBOSE_PHOSPHATE_BIOSYNTHETIC_PROCESS | 194 | 1.658537 | 0.012371134 | 0.1000778 |
| GOBP | CELLULAR_RESPONSE_TO_REACTIVE_OXYGEN_SPECIES | 152 | 1.658483 | 0.008333334 | 0.0999211 |
| GOBP | INTERLEUKIN_1_MEDIATED_SIGNALING_PATHWAY | 101 | 1.657624 | 0.046277665 | 0.1003633 |
| GOBP | THIOESTER_BIOSYNTHETIC_PROCESS | 53 | 1.657221 | 0.031578947 | 0.1004172 |
| GOBP | REGULATION_OF_DNA_TEMPLATED_TRANSCRIPTION_ELONGATION | 51 | 1.657077 | 0.041493777 | 0.10033 |
| GOBP | RESPONSE_TO_ARSENIC_CONTAINING_SUBSTANCE | 30 | 1.656448 | 0.024793388 | 0.1005963 |
| GOBP | ONE_CARBON_METABOLIC_PROCESS | 40 | 1.654824 | 0.033898305 | 0.1015163 |
| GOBP | MITOTIC_DNA_REPLICATION | 16 | 1.65345 | 0.0373444 | 0.1020654 |
| GOBP | RNA_INTERFERENCE | 17 | 1.653362 | 0.032467533 | 0.1019218 |
| GOBP | CD40_SIGNALING_PATHWAY | 15 | 1.652739 | 0.048262548 | 0.1022152 |
| GOBP | SPHINGOMYELIN_METABOLIC_PROCESS | 15 | 1.652297 | 0.03285421 | 0.1022986 |
| GOBP | POSITIVE_REGULATION_OF_MEIOTIC_CELL_CYCLE | 22 | 1.650901 | 0.038548753 | 0.1030628 |
| GOBP | GLOBAL_GENOME_NUCLEOTIDE_EXCISION_REPAIR | 26 | 1.650858 | 0.04032258 | 0.1028891 |
| GOBP | TRANSCRIPTION_BY_RNA_POLYMERASE_III | 47 | 1.650709 | 0.042918455 | 0.1027875 |
| GOBP | MITOCHONDRIAL_FISSION | 42 | 1.650591 | 0.022727273 | 0.1026787 |
| GOBP | NEUROTRANSMITTER_RECEPTOR_TRANSPORT_TO_PLASMA_MEMBRANE | 15 | 1.649936 | 0.029106028 | 0.1029096 |
| GOBP | VESICLE_TARGETING | 94 | 1.649684 | 0.027196653 | 0.1028509 |
| GOBP | PEPTIDYL_THREONINE_DEPHOSPHORYLATION | 22 | 1.649416 | 0.023454158 | 0.1026768 |
| GOBP | SCF_DEPENDENT_PROTEASOMAL_UBIQUITIN_DEPENDENT_PROTEIN_CATABOLIC_PROCESS | 93 | 1.648881 | 0.047131147 | 0.1028226 |
| GOBP | NUCLEAR_ENVELOPE_ORGANIZATION | 52 | 1.648356 | 0.046 | 0.1029759 |
| GOBP | MICROVILLUS_ORGANIZATION | 24 | 1.646994 | 0.044088177 | 0.1034451 |
| GOBP | POSITIVE_REGULATION_OF_ESTABLISHMENT_OF_PROTEIN_LOCALIZATION_TO_MITOCHONDRION | 58 | 1.646053 | 0.026422765 | 0.1038217 |
| GOBP | DNA_MODIFICATION | 118 | 1.645232 | 0.027139874 | 0.1041971 |
| GOBP | MISMATCH_REPAIR | 38 | 1.64515 | 0.042462844 | 0.1040633 |
| GOBP | VESICLE_DOCKING | 65 | 1.644973 | 0.016632017 | 0.1040228 |
| GOBP | NEGATIVE_REGULATION_OF_DNA_BIOSYNTHETIC_PROCESS | 39 | 1.644861 | 0.020408163 | 0.1039077 |
| GOBP | NUCLEOTIDE_SUGAR_METABOLIC_PROCESS | 36 | 1.644632 | 0.04040404 | 0.1038767 |
| GOBP | ENDOPLASMIC_RETICULUM_MANNOSE_TRIMMING | 16 | 1.643549 | 0.039915968 | 0.1044292 |
| GOBP | POSITIVE_REGULATION_OF_MRNA_SPLICING_VIA_SPLICEOSOME | 23 | 1.642529 | 0.049792532 | 0.1049702 |
| GOBP | PROTEIN_CONTAINING_COMPLEX_DISASSEMBLY | 328 | 1.640872 | 0.022916667 | 0.1059115 |
| GOBP | ENAMEL_MINERALIZATION | 16 | 1.637911 | 0.02 | 0.1075955 |
| GOBP | NUCLEAR_MEMBRANE_ORGANIZATION | 17 | 1.635521 | 0.03941909 | 0.1087134 |
| GOBP | REGULATION_OF_STEROID_BIOSYNTHETIC_PROCESS | 91 | 1.634788 | 0.02631579 | 0.1088019 |
| GOBP | VESICLE_CARGO_LOADING | 27 | 1.634736 | 0.046908315 | 0.1086558 |
| GOBP | MONOCARBOXYLIC_ACID_CATABOLIC_PROCESS | 131 | 1.63403 | 0.03726708 | 0.1089873 |
| GOBP | IMPORT_INTO_NUCLEUS | 163 | 1.633774 | 0.030172413 | 0.108939 |
| GOBP | POSITIVE_REGULATION_OF_INTRACELLULAR_PROTEIN_TRANSPORT | 178 | 1.633703 | 0.014256619 | 0.1087992 |
| GOBP | POSITIVE_REGULATION_OF_NUCLEOCYTOPLASMIC_TRANSPORT | 61 | 1.632258 | 0.023060797 | 0.10968 |
| GOBP | MRNA_CLEAVAGE | 21 | 1.632094 | 0.035051547 | 0.1096132 |
| GOBP | REGULATION_OF_HISTONE_H3_K4_METHYLATION | 28 | 1.631177 | 0.039045554 | 0.110076 |
| GOBP | CORTICAL_ACTIN_CYTOSKELETON_ORGANIZATION | 38 | 1.627292 | 0.03757829 | 0.1124071 |
| GOBP | ASPARTATE_FAMILY_AMINO_ACID_BIOSYNTHETIC_PROCESS | 21 | 1.626779 | 0.042016808 | 0.1125444 |
| GOBP | REGULATION_OF_T_CELL_CHEMOTAXIS | 16 | 1.626759 | 0.040650405 | 0.1123594 |
| GOBP | REGULATION_OF_INTRACELLULAR_PROTEIN_TRANSPORT | 245 | 1.626754 | 0.012552301 | 0.1121678 |
| GOBP | RESPONSE_TO_NITRIC_OXIDE | 19 | 1.62317 | 0.031380754 | 0.1146241 |
| GOBP | CELLULAR_AMINO_ACID_BIOSYNTHETIC_PROCESS | 77 | 1.621528 | 0.023861172 | 0.1155491 |
| GOBP | NEGATIVE_REGULATION_OF_FIBROBLAST_PROLIFERATION | 31 | 1.62079 | 0.029473685 | 0.1158711 |
| GOBP | COPII_COATED_VESICLE_CARGO_LOADING | 15 | 1.619909 | 0.04347826 | 0.1163415 |
| GOBP | B_CELL_APOPTOTIC_PROCESS | 25 | 1.619856 | 0.03448276 | 0.1161655 |
| GOBP | REGULATION_OF_INTRINSIC_APOPTOTIC_SIGNALING_PATHWAY_IN_RESPONSE_TO_DNA_DAMAGE_BY_P53_CLASS_MEDIATOR | 17 | 1.619849 | 0.04660194 | 0.1159638 |
| GOBP | CHEMOKINE_C_X_C_MOTIF_LIGAND_2_PRODUCTION | 16 | 1.619312 | 0.043032788 | 0.1161788 |
| GOBP | INTRINSIC_APOPTOTIC_SIGNALING_PATHWAY | 280 | 1.619254 | 0.02020202 | 0.1160258 |
| GOBP | PROTEIN_INSERTION_INTO_MEMBRANE | 77 | 1.618583 | 0.047244094 | 0.1161 |
| GOBP | PROTEIN_TARGETING | 437 | 1.616912 | 0.014285714 | 0.1171868 |
| GOBP | ESTABLISHMENT_OF_PROTEIN_LOCALIZATION_TO_MITOCHONDRIAL_MEMBRANE | 53 | 1.616203 | 0.04950495 | 0.1174765 |
| GOBP | NEGATIVE_REGULATION_OF_DNA_REPLICATION | 34 | 1.614585 | 0.044210527 | 0.1181861 |
| GOBP | GLUTAMINE_FAMILY_AMINO_ACID_BIOSYNTHETIC_PROCESS | 17 | 1.613754 | 0.036809817 | 0.1185957 |
| GOBP | SULFUR_AMINO_ACID_BIOSYNTHETIC_PROCESS | 17 | 1.6117 | 0.04680851 | 0.1196905 |
| GOBP | AUTOPHAGY_OF_MITOCHONDRION | 75 | 1.609453 | 0.034136545 | 0.1209838 |
| GOBP | ANTIGEN_PROCESSING_AND_PRESENTATION_OF_PEPTIDE_OR_POLYSACCHARIDE_ANTIGEN_VIA_MHC_CLASS_II | 102 | 1.608786 | 0.046421662 | 0.1208397 |
| GOBP | DNA_METHYLATION_OR_DEMETHYLATION | 96 | 1.607356 | 0.02631579 | 0.1216492 |
| GOBP | POSITIVE_REGULATION_OF_INTRACELLULAR_TRANSPORT | 218 | 1.606543 | 0.018947368 | 0.1220615 |
| GOBP | SERINE_FAMILY_AMINO_ACID_BIOSYNTHETIC_PROCESS | 21 | 1.60569 | 0.03877551 | 0.1224639 |
| GOBP | PEPTIDYL_PROLINE_MODIFICATION | 59 | 1.60478 | 0.016260162 | 0.1227899 |
| GOBP | POLYOL_BIOSYNTHETIC_PROCESS | 63 | 1.604708 | 0.025974026 | 0.1226519 |
| GOBP | NEGATIVE_REGULATION_OF_UBIQUITIN_PROTEIN_TRANSFERASE_ACTIVITY | 16 | 1.603624 | 0.041152265 | 0.1233575 |
| GOBP | CELLULAR_LIPID_CATABOLIC_PROCESS | 229 | 1.603081 | 0.016842104 | 0.1235757 |
| GOBP | PHOSPHATIDYLGLYCEROL_METABOLIC_PROCESS | 37 | 1.601859 | 0.011389522 | 0.1240189 |
| GOBP | REGULATION_OF_MITOCHONDRION_ORGANIZATION | 150 | 1.601005 | 0.015841585 | 0.1242323 |
| GOBP | RESPONSE_TO_OXIDATIVE_STRESS | 434 | 1.598786 | 0.00212766 | 0.1251162 |
| GOBP | LIPID_OXIDATION | 110 | 1.598629 | 0.027777778 | 0.1250258 |
| GOBP | REGULATION_OF_SPHINGOLIPID_BIOSYNTHETIC_PROCESS | 18 | 1.598628 | 0.041753653 | 0.1248208 |
| GOBP | GLYCOSYL_COMPOUND_CATABOLIC_PROCESS | 44 | 1.598021 | 0.027027028 | 0.1250664 |
| GOBP | CELLULAR_RESPONSE_TO_TOPOLOGICALLY_INCORRECT_PROTEIN | 168 | 1.597874 | 0.039832287 | 0.1249846 |
| GOBP | LONG_CHAIN_FATTY_ACYL_COA_METABOLIC_PROCESS | 25 | 1.595482 | 0.041036718 | 0.1263019 |
| GOBP | REGULATION_OF_ERAD_PATHWAY | 19 | 1.5946 | 0.040816326 | 0.1268502 |
| GOBP | POSITIVE_REGULATION_OF_AMINO_ACID_TRANSPORT | 16 | 1.591148 | 0.02690583 | 0.1292191 |
| GOBP | POLYOL_CATABOLIC_PROCESS | 25 | 1.590913 | 0.031512607 | 0.1291978 |
| GOBP | POSITIVE_REGULATION_OF_CELLULAR_PROTEIN_LOCALIZATION | 294 | 1.590191 | 0.018367346 | 0.1290631 |
| GOBP | REGULATION_OF_INTRACELLULAR_TRANSPORT | 346 | 1.589579 | 0.016736401 | 0.1291854 |
| GOBP | NEGATIVE_REGULATION_OF_ERBB_SIGNALING_PATHWAY | 52 | 1.585397 | 0.044088177 | 0.1312906 |
| GOBP | NUCLEOSIDE_TRIPHOSPHATE_METABOLIC_PROCESS | 108 | 1.582546 | 0.03846154 | 0.1328616 |
| GOBP | ESTABLISHMENT_OF_PROTEIN_LOCALIZATION_TO_MEMBRANE | 349 | 1.58217 | 0.04 | 0.1329906 |
| GOBP | MITOCHONDRIAL_TRANSCRIPTION | 17 | 1.581619 | 0.04733728 | 0.1332203 |
| GOBP | REGULATION_OF_CELLULAR_KETONE_METABOLIC_PROCESS | 184 | 1.580279 | 0.021881837 | 0.133807 |
| GOBP | RESPONSE_TO_TOPOLOGICALLY_INCORRECT_PROTEIN | 205 | 1.579509 | 0.0392562 | 0.1342182 |
| GOBP | ORGANIC_ACID_CATABOLIC_PROCESS | 257 | 1.578908 | 0.034907598 | 0.13449 |
| GOBP | RESPONSE_TO_HYDROGEN_PEROXIDE | 129 | 1.577484 | 0.014227643 | 0.1352447 |
| GOBP | ENDOPLASMIC_RETICULUM_UNFOLDED_PROTEIN_RESPONSE | 127 | 1.575671 | 0.04077253 | 0.1362701 |
| GOBP | ZINC_ION_TRANSPORT | 27 | 1.575448 | 0.03470716 | 0.1362499 |
| GOBP | ANIMAL_ORGAN_REGENERATION | 73 | 1.574016 | 0.026639344 | 0.1371458 |
| GOBP | REGULATION_OF_OXIDATIVE_PHOSPHORYLATION | 25 | 1.573562 | 0.041322313 | 0.1373027 |
| GOBP | NEGATIVE_REGULATION_OF_APOPTOTIC_SIGNALING_PATHWAY | 222 | 1.572891 | 0.008639309 | 0.1376196 |
| GOBP | POSITIVE_REGULATION_OF_SMOOTH_MUSCLE_CELL_APOPTOTIC_PROCESS | 16 | 1.572192 | 0.035639413 | 0.1380609 |
| GOBP | REGULATION_OF_OXIDATIVE_STRESS_INDUCED_INTRINSIC_APOPTOTIC_SIGNALING_PATHWAY | 28 | 1.56863 | 0.03966597 | 0.1398893 |
| GOBP | PHOSPHOLIPID_BIOSYNTHETIC_PROCESS | 269 | 1.566993 | 0.027310925 | 0.1405633 |
| GOBP | REGULATION_OF_ALCOHOL_BIOSYNTHETIC_PROCESS | 75 | 1.566384 | 0.03982301 | 0.1408528 |
| GOBP | ORGANIC_HYDROXY_COMPOUND_BIOSYNTHETIC_PROCESS | 251 | 1.564364 | 0.015486726 | 0.1421717 |
| GOBP | GLUTAMINE_FAMILY_AMINO_ACID_METABOLIC_PROCESS | 74 | 1.563116 | 0.021929825 | 0.1429782 |
| GOBP | POSITIVE_REGULATION_OF_AMYLOID_PRECURSOR_PROTEIN_CATABOLIC_PROCESS | 25 | 1.557246 | 0.04535637 | 0.1460853 |
| GOBP | RESPONSE_TO_INTERLEUKIN_7 | 38 | 1.553775 | 0.046 | 0.1483024 |
| GOBP | POSITIVE_REGULATION_OF_MITOTIC_CELL_CYCLE | 116 | 1.550827 | 0.04793028 | 0.1506784 |
| GOBP | RESPONSE_TO_IMMOBILIZATION_STRESS | 25 | 1.549761 | 0.017738359 | 0.1508251 |
| GOBP | REGULATION_OF_PROTEIN_TARGETING_TO_MITOCHONDRION | 44 | 1.549602 | 0.041237112 | 0.1505227 |
| GOBP | CELLULAR_RESPONSE_TO_UV | 88 | 1.543726 | 0.049689442 | 0.1542175 |
| GOBP | CELLULAR_IRON_ION_HOMEOSTASIS | 70 | 1.54361 | 0.025210084 | 0.1541192 |
| GOBP | REGULATION_OF_PROTEASOMAL_UBIQUITIN_DEPENDENT_PROTEIN_CATABOLIC_PROCESS | 131 | 1.542992 | 0.029106028 | 0.1542649 |
| GOBP | CELL_AGING | 108 | 1.541718 | 0.0491453 | 0.1551217 |
| GOBP | POSITIVE_REGULATION_OF_PEPTIDASE_ACTIVITY | 194 | 1.540467 | 0.030107526 | 0.1560309 |
| GOBP | RESPONSE_TO_REACTIVE_OXYGEN_SPECIES | 217 | 1.539555 | 0.01871102 | 0.1563277 |
| GOBP | ASPARTATE_FAMILY_AMINO_ACID_CATABOLIC_PROCESS | 23 | 1.539124 | 0.038 | 0.1565217 |
| GOBP | CELLULAR_CARBOHYDRATE_CATABOLIC_PROCESS | 47 | 1.536927 | 0.030864198 | 0.1581388 |
| GOBP | REGULATION_OF_ESTABLISHMENT_OF_PROTEIN_LOCALIZATION_TO_MITOCHONDRION | 72 | 1.536654 | 0.04106776 | 0.1579275 |
| GOBP | GOLGI_TO_PLASMA_MEMBRANE_PROTEIN_TRANSPORT | 39 | 1.536385 | 0.03601695 | 0.1579291 |
| GOBP | VESICLE_MEDIATED_TRANSPORT_TO_THE_PLASMA_MEMBRANE | 92 | 1.53205 | 0.04661017 | 0.1603466 |
| GOBP | PINOCYTOSIS | 22 | 1.53039 | 0.04133858 | 0.1614568 |
| GOBP | POLYOL_METABOLIC_PROCESS | 129 | 1.529374 | 0.025316456 | 0.1621432 |
| GOBP | ANATOMICAL_STRUCTURE_HOMEOSTASIS | 466 | 1.526404 | 0.023913043 | 0.1641338 |
| GOBP | REGULATION_OF_MEIOTIC_NUCLEAR_DIVISION | 30 | 1.525326 | 0.046153847 | 0.1644364 |
| GOBP | NIK_NF_KAPPAB_SIGNALING | 181 | 1.524737 | 0.048484847 | 0.1644708 |
| GOBP | REGULATION_OF_UBIQUITIN_DEPENDENT_PROTEIN_CATABOLIC_PROCESS | 160 | 1.521982 | 0.035490606 | 0.1664552 |
| GOBP | POSITIVE_REGULATION_OF_ATPASE_ACTIVITY | 58 | 1.52183 | 0.04524887 | 0.1663534 |
| GOBP | RESPONSE_TO_HEAT | 160 | 1.52088 | 0.04893617 | 0.1668248 |
| GOBP | GLYCOSYL_COMPOUND_METABOLIC_PROCESS | 129 | 1.520465 | 0.0480167 | 0.1669771 |
| GOBP | APOPTOTIC_MITOCHONDRIAL_CHANGES | 114 | 1.51524 | 0.040899795 | 0.1691416 |
| GOBP | ACTIVATION_OF_CYSTEINE_TYPE_ENDOPEPTIDASE_ACTIVITY_INVOLVED_IN_APOPTOTIC_PROCESS | 86 | 1.51438 | 0.044680852 | 0.169721 |
| GOBP | PHOSPHOLIPID_METABOLIC_PROCESS | 418 | 1.513647 | 0.018828452 | 0.1699408 |
| GOBP | CELLULAR_KETONE_METABOLIC_PROCESS | 253 | 1.51047 | 0.031982943 | 0.1725064 |
| GOBP | REGULATION_OF_PROTEIN_LOCALIZATION_TO_NUCLEUS | 130 | 1.503477 | 0.049568966 | 0.1782216 |
| GOBP | RESPONSE_TO_TEMPERATURE_STIMULUS | 227 | 1.50313 | 0.036796536 | 0.1783357 |
| GOBP | REGULATION_OF_CARBOHYDRATE_METABOLIC_PROCESS | 206 | 1.502602 | 0.042918455 | 0.1783708 |
| GOBP | NUCLEOSIDE_BISPHOSPHATE_METABOLIC_PROCESS | 139 | 1.50242 | 0.042944785 | 0.1782945 |
| GOBP | NEGATIVE_REGULATION_OF_CELLULAR_PROTEIN_CATABOLIC_PROCESS | 76 | 1.502362 | 0.045553144 | 0.1781083 |
| GOBP | REGULATION_OF_RESPONSE_TO_OXIDATIVE_STRESS | 83 | 1.500702 | 0.04793028 | 0.1784928 |
| GOBP | PYRIDINE_CONTAINING_COMPOUND_METABOLIC_PROCESS | 41 | 1.49824 | 0.030303031 | 0.1796892 |
| GOBP | RESPONSE_TO_RADIATION | 446 | 1.497931 | 0.046153847 | 0.1797384 |
| GOBP | ERYTHROCYTE_HOMEOSTASIS | 120 | 1.490879 | 0.033898305 | 0.1844772 |
| GOBP | PROTEIN_TETRAMERIZATION | 83 | 1.48798 | 0.017977528 | 0.18628 |
| GOBP | POSITIVE_REGULATION_OF_PROTEOLYSIS | 361 | 1.485895 | 0.038854804 | 0.1873894 |
| GOBP | REGULATION_OF_APOPTOTIC_SIGNALING_PATHWAY | 344 | 1.485857 | 0.027777778 | 0.1871753 |
| GOBP | BIOLOGICAL_PROCESS_INVOLVED_IN_INTERACTION_WITH_SYMBIONT | 92 | 1.478792 | 0.037209302 | 0.1911978 |
| GOBP | CELLULAR_RESPONSE_TO_OXYGEN_LEVELS | 225 | 1.478731 | 0.03797468 | 0.1910117 |
| GOBP | AGING | 296 | 1.473943 | 0.045454547 | 0.1926669 |
| GOBP | MONOSACCHARIDE_METABOLIC_PROCESS | 272 | 1.470521 | 0.041484717 | 0.1944991 |
| GOBP | CELLULAR_TRANSITION_METAL_ION_HOMEOSTASIS | 117 | 1.469462 | 0.032894738 | 0.1948173 |
| GOBP | RESPONSE_TO_FOOD | 38 | 1.466284 | 0.031026253 | 0.1965392 |
| GOBP | CELLULAR_MODIFIED_AMINO_ACID_METABOLIC_PROCESS | 197 | 1.445305 | 0.0397351 | 0.2099097 |
| GOBP | LIPID_CATABOLIC_PROCESS | 339 | 1.442523 | 0.032894738 | 0.2106212 |
| GOBP | CELLULAR_CARBOHYDRATE_METABOLIC_PROCESS | 287 | 1.441888 | 0.040084388 | 0.2110236 |
| GOBP | RESPONSE_TO_OXYGEN_LEVELS | 379 | 1.434112 | 0.038793102 | 0.2158409 |
| GOBP | REGULATION_OF_MONOOXYGENASE_ACTIVITY | 64 | 1.427753 | 0.043290045 | 0.2207619 |

| Terms | SIZE | NES | NOM p value | FDR q value |
| --- | --- | --- | --- | --- |
| HALLMARK_MTORC1_SIGNALING | 200 | 2.228139 | 0 | 0.003216556 |
| HALLMARK_E2F_TARGETS | 200 | 2.183801 | 0 | 0.001608278 |
| HALLMARK_MYC_TARGETS_V1 | 200 | 2.17931 | 0.003731343 | 0.001417303 |
| HALLMARK_G2M_CHECKPOINT | 200 | 2.134515 | 0 | 0.002425844 |
| HALLMARK_MYC_TARGETS_V2 | 58 | 2.110589 | 0 | 0.003020693 |
| HALLMARK_GLYCOLYSIS | 199 | 2.023218 | 0 | 0.011572464 |
| HALLMARK_UNFOLDED_PROTEIN_RESPONSE | 113 | 2.000778 | 0.003809524 | 0.012422876 |
| HALLMARK_OXIDATIVE_PHOSPHORYLATION | 200 | 1.904829 | 0.041044775 | 0.0259437 |
| HALLMARK_DNA_REPAIR | 149 | 1.902381 | 0.017374517 | 0.023655128 |
| HALLMARK_FATTY_ACID_METABOLISM | 158 | 1.850786 | 0.006355932 | 0.032858066 |
| HALLMARK_PROTEIN_SECRETION | 96 | 1.830424 | 0.023904383 | 0.03511622 |
| HALLMARK_MITOTIC_SPINDLE | 199 | 1.794706 | 0.038240917 | 0.041661806 |
| HALLMARK_CHOLESTEROL_HOMEOSTASIS | 74 | 1.767611 | 0.006329114 | 0.0462935 |
| HALLMARK_PEROXISOME | 104 | 1.715797 | 0.006276151 | 0.05942851 |
| HALLMARK_UV_RESPONSE_UP | 158 | 1.64163 | 0.022821577 | 0.08654617 |
| HALLMARK_SPERMATOGENESIS | 135 | 1.632411 | 0.02027027 | 0.08590548 |
| HALLMARK_ADIPOGENESIS | 200 | 1.536961 | 0.049407113 | 0.1186785 |

| Terms | SIZE | NES | NOM p value | FDR q value |
| --- | --- | --- | --- | --- |
| KEGG_CYSTEINE_AND_METHIONINE_METABOLISM | 34 | 2.148859 | 0 | 0.028429 |
| KEGG_AMINOACYL_TRNA_BIOSYNTHESIS | 41 | 2.118604 | 0.00182482 | 0.024426 |
| KEGG_CELL_CYCLE | 124 | 2.094303 | 0 | 0.021771 |
| KEGG_GLYOXYLATE_AND_DICARBOXYLATE_METABOLISM | 16 | 2.041368 | 0 | 0.034133 |
| KEGG_TERPENOID_BACKBONE_BIOSYNTHESIS | 15 | 2.030953 | 0 | 0.031549 |
| KEGG_VALINE_LEUCINE_AND_ISOLEUCINE_DEGRADATION | 44 | 2.015663 | 0 | 0.031739 |
| KEGG_RNA_DEGRADATION | 59 | 1.994736 | 0.00192308 | 0.034467 |
| KEGG_PYRIMIDINE_METABOLISM | 97 | 1.970561 | 0.0040404 | 0.037902 |
| KEGG_ONE_CARBON_POOL_BY_FOLATE | 17 | 1.967717 | 0 | 0.034702 |
| KEGG_FRUCTOSE_AND_MANNOSE_METABOLISM | 33 | 1.951168 | 0.00804829 | 0.03637 |
| KEGG_SPLICEOSOME | 127 | 1.9298 | 0.01584159 | 0.038859 |
| KEGG_BASE_EXCISION_REPAIR | 35 | 1.919418 | 0.00793651 | 0.038675 |
| KEGG_DNA_REPLICATION | 36 | 1.895502 | 0.00775194 | 0.04345 |
| KEGG_STEROID_BIOSYNTHESIS | 17 | 1.880109 | 0.004 | 0.047262 |
| KEGG_NUCLEOTIDE_EXCISION_REPAIR | 44 | 1.874498 | 0.01375246 | 0.047064 |
| KEGG_GALACTOSE_METABOLISM | 26 | 1.869926 | 0.00819672 | 0.046247 |
| KEGG_HOMOLOGOUS_RECOMBINATION | 28 | 1.833941 | 0.02794411 | 0.057872 |
| KEGG_OOCYTE_MEIOSIS | 112 | 1.832634 | 0.00387597 | 0.055547 |
| KEGG_SELENOAMINO_ACID_METABOLISM | 26 | 1.819924 | 0.00795229 | 0.058055 |
| KEGG_CITRATE_CYCLE_TCA_CYCLE | 31 | 1.815355 | 0.02251407 | 0.057734 |
| KEGG_PROPANOATE_METABOLISM | 33 | 1.760104 | 0.01308411 | 0.082588 |
| KEGG_P53_SIGNALING_PATHWAY | 68 | 1.746961 | 0.01377953 | 0.087031 |
| KEGG_OTHER_GLYCAN_DEGRADATION | 16 | 1.744489 | 0.01612903 | 0.084888 |
| KEGG_RNA_POLYMERASE | 28 | 1.734411 | 0.02620968 | 0.087548 |
| KEGG_LYSINE_DEGRADATION | 44 | 1.734044 | 0.01369863 | 0.084305 |
| KEGG_GLYCOLYSIS_GLUCONEOGENESIS | 62 | 1.711579 | 0.02083333 | 0.094683 |
| KEGG_PYRUVATE_METABOLISM | 40 | 1.70919 | 0.0212766 | 0.092272 |
| KEGG_AMINO_SUGAR_AND_NUCLEOTIDE_SUGAR_METABOLISM | 43 | 1.688736 | 0.03769841 | 0.10219 |
| KEGG_PURINE_METABOLISM | 158 | 1.687186 | 0.00801603 | 0.099814 |
| KEGG_PROGESTERONE_MEDIATED_OOCYTE_MATURATION | 85 | 1.673689 | 0.02840909 | 0.106513 |
| KEGG_UBIQUITIN_MEDIATED_PROTEOLYSIS | 134 | 1.669482 | 0.04743083 | 0.105098 |
| KEGG_BUTANOATE_METABOLISM | 34 | 1.656874 | 0.03294574 | 0.109765 |
| KEGG_PANTOTHENATE_AND_COA_BIOSYNTHESIS | 16 | 1.571907 | 0.04731183 | 0.159556 |

GO: Gene Ontology; KEGG: Kyoto Encyclopedia of Genes and Genomes; GSEA: gene set enrichment analysis.
